# Sensors and Artificial Intelligence Methods and Algorithms for Human–Computer Intelligent Interaction: A Systematic Mapping Study

**DOI:** 10.3390/s22010020

**Published:** 2021-12-21

**Authors:** Boštjan Šumak, Saša Brdnik, Maja Pušnik

**Affiliations:** Faculty of Electrical Engineering and Computer Science, University of Maribor, 2000 Maribor, Slovenia; sasa.brdnik@um.si (S.B.); maja.pusnik@um.si (M.P.)

**Keywords:** human–computer intelligent interaction, intelligent user interfaces, IUI, sensors, artificial intelligence

## Abstract

To equip computers with human communication skills and to enable natural interaction between the computer and a human, intelligent solutions are required based on artificial intelligence (AI) methods, algorithms, and sensor technology. This study aimed at identifying and analyzing the state-of-the-art AI methods and algorithms and sensors technology in existing human–computer intelligent interaction (HCII) research to explore trends in HCII research, categorize existing evidence, and identify potential directions for future research. We conduct a systematic mapping study of the HCII body of research. Four hundred fifty-four studies published in various journals and conferences between 2010 and 2021 were identified and analyzed. Studies in the HCII and IUI fields have primarily been focused on intelligent recognition of emotion, gestures, and facial expressions using sensors technology, such as the camera, EEG, Kinect, wearable sensors, eye tracker, gyroscope, and others. Researchers most often apply deep-learning and instance-based AI methods and algorithms. The support sector machine (SVM) is the most widely used algorithm for various kinds of recognition, primarily an emotion, facial expression, and gesture. The convolutional neural network (CNN) is the often-used deep-learning algorithm for emotion recognition, facial recognition, and gesture recognition solutions.

## 1. Introduction

Human–computer interaction (HCI) is a research area related to people’s design and use of technology and computers [1]. With the rapid development of modern information and communication technologies the role and purpose of computers has significantly changed. Today, computers have become indispensable for everyday learning, job, social, and life activities. Regardless of the type of device (e.g., a desktop computer, a laptop, a smartphone, etc.), the computer programs need to provide user interfaces (UIs) with an efficient HCI, enabling efficient completion of various tasks, such as typing a document, driving a car, controlling a robot arm, searching information online, listening to music, and others. By the massive infusion of technologies in today’s society and their ubiquitous usage in our daily-life activities, the HCI has become one of the most exciting research topics in recent years [2]. The appeal for useful HCI technologies has led numerous researchers to develop innovative and intelligent methods to make HCI attractive to users [3].

In recent years, we have been witnessing a growing need for intelligent user interfaces (IUIs) that can meet user criteria where the user’s expectation towards the technology increases day-by-day [4]. Next-generation computing has made it possible to develop anticipatory human-centered UIs built for humans on naturally occurring multimodal human communication, which is able to understand and emulate human communicative intentions as expressed through behavioral cues, such as effective and social signals [5]. The vision of developing IUI emerged with the creation of the first computers [6] and has been evolving ever since, with the development of UI enabling different types of interaction with software systems, as well as with the evolution of artificial intelligence (AI) and adaptation mechanisms [7]. IUI provides many benefits to the users, including adaptivity, context-sensitivity, task assistance, and enhanced user-interaction through comprehended input, generated multimodal presentation, semi- or fully automated completion of tasks, and managed interaction by representing, reasoning, and exploiting models of user, domain, task, and context [6]. The rapid development of Internet of Things (IoT) technologies has spurred the development of devices and sensors that surround the users and provide valuable information about users’ actions, behavior, interests, and preferences. AI machine-learning methods and algorithms can leverage such information to develop IUIs, providing increased productivity, improved efficiency, effectiveness, and naturalness of interaction.

The IUI field is multi-disciplinary, interchanging the ideas from different areas, such as HCI, ergonomics, cognitive science (CS), and AI, with its subfields such as vision, speech and language processing, knowledge representation and reasoning, machine learning/knowledge discovery, planning and agent-modeling, and user and discourse modeling [6,8]. The need for intelligence in UIs has also driven the research and development of new AI methods and algorithms, enabling the development of human–computer intelligent interactions (HCII) and IUIs. The data generated during the user’s interaction with the computer through intelligent sensing technology can be processed with AI to recognize HCI patterns that can be used to adjust the UI according to individual needs, preferences, and styles of using the UI. The main objective of this study is to identify and systematically map the research in the field of HCII and IUI. The insights might provide an overview of trends in which HCII and IUI are studied concerning the ongoing discussion about the use of sensing technology and AI methods and algorithms used in developing HCII and IUI. In existing HCI- and IUI-related research, systematic literature reviews and systematic mapping studies have investigated, for example, design approaches in human-centric IT systems [9], the meaning of the term “intelligent” in the HCI community [10], understanding of research status of interaction design in HCI [11], the thematic evolution of the HCI and related fields in CHI conference community [12], the design trends of IUIs in the context of contemporary software systems [7], applications IUIs with emphasis on ambient-assisted living technologies [13], affective computing methods and techniques [14], deep-learning methods for EMG-based human–machine interaction [15], emotion recognition using EEG signals [16], applications of AI in smart home solutions [17], deep-learning techniques for speech emotion recognition [18], and mobile sensing and emotion recognition via smartphone sensors [19,20,21]. However, to our best knowledge, this study is one of the first to provide a systematic review and mapping of existing research published in the last decade in the field of HCII, focusing on sensing technology and AI methods and algorithms to develop IUI. This paper can enable both HCI researchers and AI researchers gain wider and deeper insight into HCII research in the last decade, facilitating relevant researchers to have a general understanding of novel sensing technology and AI methods and algorithms used in HCII development and find future research directions.

## 2. Backgrounds and Related Work

### 2.1. Human–Computer Intelligent Interaction (HCII)

HCI is “a discipline concerned with the design, evaluation, and implementation of interactive computing systems for human use and with the study of major phenomena surrounding them” [22]. HCI is an interdisciplinary discipline grounded initially on computer science, psychology, and ergonomics and disciplines that joined later, for example, social science, cognitive science, etc. [23]. In the HCI, the user’s activity includes the following aspects [24]: (1) physical, which determines the mechanics of the interaction between human and computer; (2) cognitive, which deals with the way that users can understand the system and interact with it; and (3) affective, which tries to make the interaction pleasurable to the user as well as to affect the user in a way that makes the user continue to use the machine by changing attitudes and emotions.

HCI is a two-way communication: (1) computer to user, and (2) user to computer. In computer to user communication, the main challenge is how to present the information efficiently. Modern UIs with innovative interface technology (e.g., virtual reality, 3D displays, etc.) have enabled new ways of information delivery to the user. A virtual agent or avatar that can mimic human behavior is another example of innovative solutions for delivering information to users. In the user to computer communication, the main challenge is enabling the user to command the computer to do things naturally. By combining human-centered design with leading-edge technologies, UIs move from keyboards, mouses, and touchscreens to IUIs that use different modalities for computer commands, including voice recognition, computer vision, and others. Novel HCII systems equipped with AI methods and techniques can respond to verbal commands (e.g., speech-based systems, such as Alexa from Amazon [25]), and non-verbal commands (e.g., Soli from Google [26]).

**Figure 1:**
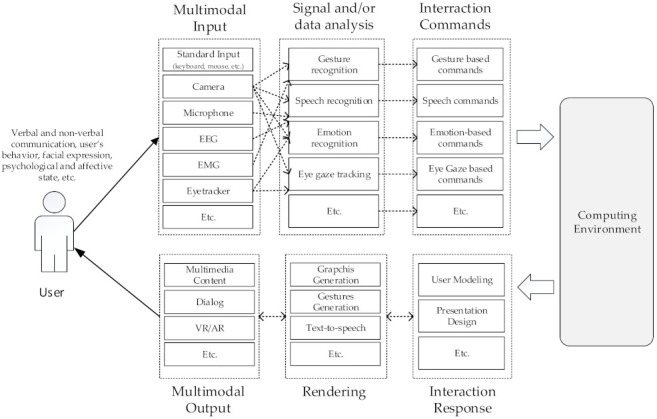
A general architecture of a multimodal HCII system (adapted from [6]).

Figure 1 presents an example of a multimodal HCII system architecture that provides multimodal input/output capabilities for intelligent interaction with the user. HCII systems support multimodal input/output capabilities that, compared to standard HCI systems based on a keyboard and a mouse, provide more flexible and expressively powerful interaction with a computer. The HCII system usually provides a multimodal input enabling user input and processing of two or more modalities (e.g., touch, gaze, body movement, virtual keyboard, etc.). The user input can be based on standard simple-input input devices (e.g., keyboard, mouse, touch, etc.), recognition-based technologies (e.g., speech, gesture, emotion, etc.), or sensor-based technologies (e.g., acceleration, pressure, brain signal, etc.) [1]. HCII systems also support multimodal or multimedia output involving two or more types of information received as feedback by a user during HCI. Multimedia output can provide different media types within one modality, such as vision (e.g., still images, virtual reality, video images, etc.) or multimodal output, such as visual, auditory, and tactile feedback to the user [1].

In the last decade, there has been a rapid increase in existing literature in the HCII field. HCII aims to provide natural ways for humans to use computers and technology in all aspects of future peoples’ life and is relevant in applications, such as smart homes, smart offices, virtual reality, education, and call centers [27,28]. For an effective HCII, computers must have the communication skills of humans [29] to be able to interact with the users naturally [30] by enabling interactions that are able to mimic human–human interactions [27]. HCII solutions must implement at least some kind of intelligence in perception from and/or in response to the users [24].

A natural human–human interaction consists of a mix of verbal signals (e.g., speech, intonation, etc.) and non-verbal signals (e.g., gestures, facial expression, eye motions, body language, etc.). Nonverbal information can be used for predicting and understanding a user’s inner (cognitive and affective) state of the mind [31]. In order to provide genuinely intuitive communication, computers need to have their sense of verbal and non-verbal signals in order to understand the message and the context of the message [32]. A robust, efficient, and effective HCII system must therefore be able to activate different channels (e.g., auditory channels which carry speech and vocal intonation, a visual channel that carries facial expression and gestures, etc.) and modalities (e.g., sense of sight, hearing, etc.) that enable effective detection, recognition, interpretation, and analysis of various human physiological and behavioral characteristics during the interaction [33,34].

The research and developments in both hardware and software have enabled the use of speech, gestures, body posture, different tracking technology, tactile/force feedback devices, eye-gaze, and biosensors to develop new generations of HCI systems and application [33]. HCI systems that use only one type of input/output or modality enabling the HCI are called unimodal systems. Multimodal HCI systems, on the other hand, use many input or output modalities to communicate with the user, exhibiting some form of intelligent behavior in a particular domain [24,33]. Based on the way of information transfer from user to the computer, UIs can be divided into [35]: (1) contact-based interfaces (e.g., keyboard and mouse based interfaces, touch screen interfaces, etc.), (2) speech-based interfaces (e.g., spontaneous speech, continuous speech, acoustic nonspeech sounds, etc.), (3) gesture-based interfaces (e.g., finger pointing, spatial hand gestures, sign language, head gestures, user behavior, etc.), (4) facial expression-based interfaces (e.g., facial expressions, including those reflecting emotions, articulation of lips, gaze direction, eye winking, etc.), (5) textual and hand-writing interfaces (e.g., handwritten continuous text, typed text, etc.), (6) tactile and myo interfaces (e.g., sensor gloves and body-worn sensors, EMG sensors, etc.), and (7) neural computer interfaces (e.g., EEG signal, evoked potential, etc.).

Based on the nature of the modalities used, the HCIs can be divided into (1) visual-based HCI, which use various visual information about human’s response while interacting with the machine, (2) audio-based HCI that use information acquired by different audio signals, and (3) sensor-based HCI that combine a variety of areas with at least one physical sensor used between user and machine to provide the interaction [24]. The visual-based HCI research deals with the development of solutions for an efficient understanding of various humans’ responses from visual signals, including facial expression recognition, gesture recognition, gaze detection, and other areas. The audio-based HCI research includes speech recognition, speaker recognition, audio-based emotion recognition, and others. In sensor-based HCI, the solutions are being built using various sensors, which can be very primitive (e.g., a pen, a mouse, etc.) or sophisticated (e.g., motion-tracking sensors, EMG sensors, EEG devices, etc.).

Application fields of HCII are heterogeneous, and the creation of intelligent user interfaces aim to [36]: (1) change the way information is displayed based on users’ habits in a particular operating environment, (2) improve human–computer interaction by processing natural language, (3) enabling HCI for users with limitations to interact with technological devices (e.g., improvement of accessibility of interfaces for blind users, using different sensors for acquiring data about user movements and translation of movements into commands sent to a wheelchair, interfaces for cognitive impaired users, etc.). More natural and efficient intelligent interaction paradigms, such as gesture interaction, voice interaction, and face recognition, are widely being implemented in new HCI applications (e.g., smart home solutions, autonomous cars, etc.) [37]. In contrast to the conventional mechanisms of passive manipulation, HCII integrates versatile tools, such as perceptual recognition, AI, affective computing, and emotion cognition, to enhance the ways humans interact with computers [38,39]. 

Novel IUIs are not necessarily being built for replacing traditional interfaces that use input devices, such as a mouse and a keyboard, but are, rather, complementing when needed or appropriate. Solutions that enable users using speech and hand gestures to control the computer are useful especially in virtual environments, because, for example, in a 3D environment a keyboard and a mouse as an input device are not much useful. Speech recognition solutions that can recognize speech through visual signal (e.g., reading from lips) can complement speech recognition from audio signals in noisy environments where the recognition from audio signal cannot perform well. Intelligent HCI systems can also be used for enabling efficient human-to-human interaction when this is not possible due to the limitations of end-users. For example, an intelligent HCI system that combines AI with wearable devices (e.g., data gloves) can solve communication problems between a hard-of-hearing and a non-disabled person [40]. Mobile IUI solutions today make use of the plethora of advanced sensors available in smartphones, such as camera, microphone, keyboard, touchscreen, depth sensors, accelerometer, gyroscope, geolocation sensor, barometer, compass, ambient light sensor, proximity sensor, etc., which allow the combination of inputs and enrichment of HCII interactions [21]

As discussed above, the essential functions of HCII are based on a clear signal of emotional state to infer a person’s emotional state [30]. Emotions are complex processes comprised of numerous components, including feelings, body changes, cognitive reactions, behavior, and thoughts [41]. Emotion is a psycho-physiological process triggered by the conscious and unconscious perception of a situation or an object and is often associated with mood, temperament, personality, disposition, and motivation [42]. Intelligent systems providing HCII must, for example, through emotion recognition, be able to perceive the user’s emotions, produce the ability of empathy, and respond appropriately [30,43]. By understanding emotions in natural interactions, HCII systems can make smarter decisions and provide better interactive experiences [28]. Emotional interaction makes the human–computer interaction more intelligent—it makes the interaction natural, cordial, vivid, emotional [43]. Because automatic emotion recognition has many applications also in HCII, it has attracted the recent hype of AI-empowered HCI research [44]. Another essential and challenging task related to emotion recognition in HCII is speech emotion recognition [45], which has become the heart of most HCI applications in the modern world [46]. For many years, eye-tracking technology has been used for usability testing and implementation of various solutions for controlling the user interface. Eye-tracking-based UIs are, for example, various assistive technology solutions for people with severe disabilities (e.g., [47]) that cannot use arms and standard input devices. However, eye-based cues (e.g., eye gaze) are another field of increasing interest to the research community for automatic emotion classification and affect prediction [48]. 

Facial expression is a powerful, natural, and direct way humans communicate and understand each other’s affective states and intentions [29]. Facial expression is considered a significant gesture of social interaction and one of the most significant nonverbal behaviors, through which HCI systems may recognize human emotions’ internal or affective state [49]. The clues for understanding facial expressions lie not in global facial appearance but also in informative local dynamics among different but confusing expressions [38]. Automatic facial expression recognition plays a vital role in HCII, as it can help non-intrusively apprehend a person’s psychopathology [50]. Motivated by this significant characteristic of instantly conveying nonverbal communication, facial expression recognition plays an intrinsic role in developing the HCII and social computing fields [51] and is becoming a necessary condition for HCII [50]. With many applications in day-to-day developments and other areas, such as interactive video, virtual reality, videoconferencing, user profiling, games, intelligent automobile systems, entertainment industries, etc., facial expression has an essential role in HCII [52].

Human gesture recognition has also become a pillar of today’s HCII, as it typically provides more comfortable and ubiquitous interaction [2]. Human gestures include static postures (e.g., hand posture, head pose, and body posture) and dynamic gestures (e.g., hand gestures, head gestures like shaking and nodding, facial action like raising the eyebrows, and body gestures) [53]. Hand gestures, for example, have been widely acknowledged as a promising HCI method [54]. Information about head gestures obtained from head motion is valuable in various applications, such as autonomous driving solutions or assistive tools for disabled users [55]. 

Furthermore, in existing research various data sources, sensors, and advanced AI methods and algorithms for innovative solutions for HCII systems have been proposed, such as the user’s activity recognition (e.g., [56,57]), depression recognition (e.g., [58,59,60,61]), affection recognition (e.g., [62]), speech recognition (e.g., [63]), user’s intention recognition (e.g., [64,65]), and others.

### 2.2. Sensors Technology for HCII

As stated in the previous section, the user input can be based on standard input devices, recognition-based technologies, or sensor-based technologies. Recognition-based technologies can be implemented using invasive methods and non-invasive methods. Invasive recognition-based technologies use sensors attached to a person (e.g., accelerometer sensor attached to the chest, waist, or different body parts). In contrast, non-invasive recognition-based technologies use non-attached sensors, e.g., vision-based sensors, such as a camera, thermal infrared sensor, depth sensor, smart vision sensor, etc. [66]. Sensor-based HCI technologies are built using various sensors, which can be very primitive (e.g., a pen, a mouse, etc.) or very sophisticated (e.g., motion tracking sensors, EMG sensors, EEG sensors, etc.).

HCI devices with sensor capabilities can be divided into [67]: standard input/output devices (e.g., mouse, keyboard, touch screen, etc.), wearables (e.g., smartwatch, smartphone, band, glove, smart glasses, etc.), non-wearables (e.g., camera, microphone, environmental sensors, etc.). Wearable devices with different kinds of built-in sensors (mechanical, physiological, bioimpedance, and biochemical) can provide data about the physical and mental state of the user [68]. Wearable sensors, for example, are increasingly being used for measuring, in particular, biological signals, such as heart rate or skin conductance [69].

Sensors can be divided into unimodal sensors, providing data about one single signal (e.g., accelerometer), and multimodal sensors (e.g., Body Area Sensor Network [70], Kinect, RespiBAN [71], Empatica E4 [71], etc.). An accelerometer sensor is a device that captures vibrations and orientation of systems that move or rotate. The accelerometer sensor has been used to study activity recognition and physical exercise trackings, such as aerobic exercises [72], gesture recognition [53], human activity recognition [73], and others. The Kinect sensor consists of an RGB camera, a depth sensor, an infrared sensor, and a microphone array. The depth sensor measures the three-dimensional positions of objects in its space [72]. Sensory used for activity recognition are typically classified as ambient and wearable sensors, where ambient sensors are attached to objects in the environment with which users interact [74].

With the development of AI, new types of sensors and interactive devices emerged, enabling new ways for interaction, such as biometrics-based interaction that includes face recognition, fingerprint recognition, attitude recognition, and so on [37]. It is sometimes argued that facial expression and tone of voice are also biological signals [69]. Multimodal HCII systems usually enable technologies for processing active input mode with recognition-based and sensor-based input technologies and technologies for processing passive input using data from sensors (e.g., biosensors, ambient sensors, etc.) [5].

In the HCII literature, various wearables have enabled the development of different recognition-based input modalities, such as hand-gesture recognition (e.g., wrist contour sensor device [75], a wearable band with 6-axis [76]), gesture-recognition in the ambient environment (e.g., haptic feedback + camera [77]), head gesture recognition (e.g., MPU-6050 inertial sensor placed on audio headset [55]), human body-posture recognition (e.g., accelerometer sensor attached to the chest, waist, or several body parts [66]), stress-detection (e.g., Empatica E4 wristband [71,78], skin conductance sensor mounted on finger [79], etc.), human–motion recognition (e.g., hierarchical helical yarn (HHY) sensor attached to different positions of the human body [80], RespiBAN (chest-worn) [71]). Hand gesture recognition can also be implemented using sensors technology, such as leap-motion sensor [81,82,83], accelerometer [76], RadSense (an end-to-end and unobtrusive system that uses Doppler radar-sensing) [84], surface electromyogram (sEMG) [54,85], and so on.

Biometric sensors provide essential data that can be used to implement various solutions for recognizing users’ physiological and psychological states during the interaction, which can be used in various HCI scenarios. Emotion-detection can be implemented, for example, by processing data from electroencephalography (ECG) sensor [86], galvanic skin response (GSR) sensor [86], electromyographic (EMG) sensor [86], photoplethysmography (PPG) sensor [86,87,88], multi-biological sensor (e.g., PolyG-I (LAXTHA Inc., Daejeon, Korea) [30], BIOPAC MP150 [89]) providing different physical signals including EEG, ECG, EMG, PPG, GSR, and respiration (RESP). Biometric sensors were also successfully applied for implementing hand gesture-recognition solutions based on the eEMG sensor (e.g., [54,85]) and solutions for human-health monitoring [70].

Audio and visual-based input modalities implemented using sensors were developed as well, such as speech recognition based on the audio signal acquired with a microphone (e.g., [63,90,91]), facial expression recognition based on processing visual data from the camera [92], human body posture recognition using data from the conventional gray level or color camera, thermal infrared sensor, depth sensor, smart vision sensor [66], user-movement recognition using Kinect [72], gesture recognition based on data from depth sensor [93] and USB camera on a helmet [94], emotion recognition with a laptop camera [91], and so on. The Kinect can also be used to implement a solution for contact-free stress recognition, where the Kinect can provide respiration signals under different breathing patterns [95]. Eyetracker was used for implementing various HCII solutions, such as cognitive-load assessment during the interaction [96], contactless measurement of heart rate variability from pupillary fluctuations [97], assistant virtual keyboard [98], adaptive UIs [96], autism spectrum disorder prediction [99], etc. 

In HCII literature, several solutions for stress recognition were proposed by processing data from various sensors. The physiological response reflects the sympathetic nervous system that can be measured by an ECG sensor, a respiration band sensor, and electrodermal activity (EDA) sensor [79]. In [100] the authors proposed a solution for stress recognition based on keystroke dynamics. Several stress-recognition solutions have combined multi sensors, e.g., visual images from a laptop camera and speech from a laptop microphone [91], a wrist sensor (accelerometer and skin conductance sensor) [101], multimodal wearable sensor (EEG, camera, GPS) [78], a RespiBAN (chest-worn) and Empatica E4 (wrist-worn) sensor (ECG, EDA, EMG, Respiration and Temperature) [71], webcam, Kinect, EDA, and GPS sensor [102], BIOPAC MP150 (ECG from wrist, EMG from corrugator muscle, GSR from fingertips) and video from camera for offline analysis [89], etc.

To enhance the quality of the communication and maximize the user’s well being during his or her interaction with the computer, the machine must understand the user’s state and automatically respond intelligently. For this, various data about the user’s behavior and state related to the interaction must be collected and analyzed. For example, understanding users’ emotions can be achieved through various measures, such as subjective self-reports, face tracking, voice analysis, gaze-tracking, and the analysis of autonomic and central neurophysiological measurements [103]. 

Humans’ emotional reactions during HCI can trigger physiological changes that can be recognized using various modalities such as facial expressions, facial blood flow, speech, behavior (gesture/posture), and physiological signals. In existing HCII research the behavioral modeling and recognition uses various physiological signals, including the electrocardiogram (ECG), electromyogram (EMG), electroencephalogram (EEG), galvanic skin response (GSR), blood volume pressure (BVP), heart rate (HR) or heart rate variability (HRV), temperature (T), and respiration rate (RR) [41]. The physiological signals respond to the human body’s central nervous system and automatic nervous system, which are voluntary reactions and are more objective [104].

Emotions, for example, can trigger some minor changes in facial blood flow with an impact on skin temperature [42] and speech [105]. EEG-based emotion recognition, for example, has become crucial in enabling the HCII [44] and has been globally accepted in many applications, such as intelligent thinking, decision-making, social communication, feeling detection, affective computing, etc. [106]. Facial expression recognition can involve using sensors, such as cameras, eye-tracker, ECG, EMG, and EEG [107]. The emotion recognition process often includes sensors for detecting physiological signals, which are not visible in the human eye and immediately reflect the emotional changes [44]. Some of the current approaches to emotion recognition based on EEG mostly rely on various handcrafted features extracted over relatively long-time windows of EEG during the participants’ exposure to appropriate affective stimuli [103]. 

One of the main challenges in HCII is the measurement of physiological signals (or biosignals), where the collection process uses invasive sensors that need to be in contact with the human body while recording. However, the ongoing research has enabled the use of non-invasive sensors as well. For example, innovative sensors, such as eye-trackers, enable the development of IUIs able to extract valuable and usable patterns of the users’ habits and ways of interaction [64]. The non-invasive sEMG signal can be used to analyze the active state of the muscles and neural activities and performs well in artificial control, clinical diagnosis, motion detection, and neurological rehabilitation [54]. Hand gesture recognition can be implemented by using a vision camera or wearable sensors [108]. Wearable sensors and gesture recognition techniques have been used to develop wearable motion sensors for the hearing- and speech-impaired and wearable gesture-based gadgets for interaction with mobile devices [109]. Recently, depth-based gesture recognition has received much intention in HCII as well [110]. With the rapid development of IoT technologies, many intelligent sensing applications have emerged, which realize contactless sensing [111].

### 2.3. Artificial Intelligence (AI) Methods and Algorithms for HCII

Artificial Intelligence (AI) is one of the most crucial components in the development of HCII and has already significantly impacted how users use and perceive contemporary IUI. The introduction of affective factors to HCIs resulted in developing an interdisciplinary research field, often called affective computing, which attempts to develop human-aware AI that can perceive, understand, and regulate emotions [112]. Once computers understand humans’ emotions, AI will rise to a new level [113]. In the HCSII research field, there is an increasing focus on developing emotional AI in HCI since emotion recognition using AI is a fundamental prerequisite to improve HCI [106]. 

Machine-learning (ML) algorithms and methods can be categorized according to the learning style or similarity in form or function. When categorizing based on the learning style, ML approaches can be divided to following three categories [114,115]:

1. Supervised learning (SL) algorithms including classification, support vector machine (SVM), discriminant analysis, naïve Bayes (NB), k-Nearest Neighbor—k-NN, regression, linear regression (LR), ensemble algorithms, decision trees (DT), artificial neural network (ANN), extreme machine learning (ELM), relevance vector machine (RVM), Gaussian processes (GP), combined algorithms, etc.,
2. Unsupervised learning (UL) algorithms that include clustering, hierarchical ML, unsupervised Gaussian mixture (UGM), hidden Markov model—HMM, K-means, fuzzy c-means, neural networks (NN), etc.,
3. Reinforcement learning (RL) algorithms that include model-based RL, model-free RL, and RL-based adaptive controllers.

ML algorithms can also be categorized into single method-based algorithms and hybrid method-based ML algorithms [116]. Single method-based algorithms include fuzzy logic (FL), ANN (e.g., perceptron, multilayer perceptrons—MLP, etc.), deep learning algorithms (DLA) (e.g., convolutional neural network—CNN, recurrent neural networks—RNNs, long short-term memory networks—LSTMs, etc.), Bayesian network (BN), genetic algorithm (GA), kernel method (e.g., SVM), logistic regression (LoR), and DT (e.g., J-48graft, random forest—RF). Hybrid method-based algorithms on the other side include fuzzy logic and natural language processing (FL-NLP), Bayesian network and recurrent neural network, long short-term memory and neural networks (LSTM-NN), etc.

Based on the similarity in terms of the algorithm’s’ function, ML algorithms can be divided into *regression algorithms* (e.g., LR, LoR, etc.), *instance-based algorithms* (e.g., k-NN, SVM, etc.), *regularization algorithms* (e.g., elastic net), *decision tree algorithms* (e.g., cClassification and regression tree, C4.5 and C5.0, etc.), *Bayesian algorithms* (e.g., NB, Gaussian NB, etc.), *clustering algorithms* (e.g., k-Means, k-Medians, etc.), *association rule learning algorithms* (e.g., Apriori algorithm), ANN algorithms (e.g., perceptron, MLP, RNN, etc.), *dimensionality reduction algorithms* (e.g., principal component analysis—PCA), *ensemble algorithms* (e.g., Adaboost), *algorithms based on probabilistic models* (e.g., Monte Carlo) and *probabilistic graphical models* (e.g., Bayesian network—BN), *genetic algorithms*, *fuzzy logic*- *based algorithms*, and *other ML algorithms* (e.g., feature selection algorithm, optimization algorithms, etc.). 

In existing HCII- related research, various methods and algorithms have been proposed to accomplish the task of facial expression classification, including SVM, k-NN, NN, rule-based classifiers, and BN [51]. For facial emotion recognition, for example, CNN was recognized as an effective method that can perform feature extraction and classification simultaneously and automatically discover multiple levels of representations in data [50]. Audio-video emotion recognition, for example, is now researched and developed with deep neural network modeling tools [117]. In the speech emotion recognition field, there is considerable attention in digital signal processing research. Researchers have developed different methods and algorithms for analyzing the emotional condition of an individual user with the focus on emotion classification by salient acoustic features of speech. Most researchers in speech emotion recognition have applied handcrafted features and machine learning techniques in recognizing speech emotion [46]. In existing speech emotion recognition research, classical ML classifiers were used, such as the Markov model (MM), Gaussian mixed model (GMM), and SVM [118]. Existing research has demonstrated that DLA effectively extract robust and salient features in the dataset [46]. After the ANN breakthrough, especially CNN, the neural approach has become the main one for creating intelligent computer vision systems [119]. CNN is currently the most widely used deep-learning model for image recognition [63].

This study is interested in AI methods and techniques for HCII solutions available in the existing literature. We are interested in both HCII solutions validated using data from sensor technology and HCII solutions validated using data from publicly available databases.

### 2.4. Related Studies

In the existing literature in HCI and HCII, several studies of systematic literature reviews (SLR) and systematic mapping studies (SMS) have been conducted in the last ten years. This fact indicates that the large body of work encouraged researchers to create a joint knowledge base in this field. Although we can find some parallels between the existing SLR and SMS studies and our study, as the existing studies also dealt with a specific topic related to HCI and HCII, certain differences make our study the first such study in HCII. 

We see the first difference when reviewing the keywords based on which existing SLR and SMS studies have systematically acquired and analyzed the existing literature. The most common keywords were HCI (in 7 studies), followed by AI (in 3 studies), IoT (in 3 studies), and EMG (in 3 studies). Others were IUI (in 2 studies), robustness (in 2 studies), accuracy (in 2 studies), smart home (in 2 studies), and deep learning (in 2 studies), followed by over 40 different keywords, particular to different domains, indicating that HCI is an important research field and is integrated into various domains. Furthermore, none of the existing studies aimed to analyze HCII and IUI literature in general and provide a standard overview of sensor technology and machine-learning methods and algorithms used for other HCII developments.

In [9], authors investigated how to approach the design of human-centric IT systems and what they represent. A study published ten years later [10], has still been questioning what is actually deemed intelligent, and surprisingly still examining the question that should have been answered a decade ago. In one of the most extensive reviews of literature related to the HCI field [120], authors analyzed 3243 articles, examining publication growth, geographical distribution, citation analysis research productivity, and keywords, and identified the following five clusters: (1) UI for user centric design, (2) HCI, (3) interaction design, (4) intelligent interaction recognition research, and (5) e-health and health information. The main conclusion in [120] was that the research in this field has little consistency, the researchers start addressing newer technologies, and there is no accumulated knowledge. A similar attempt at visualizing popular clusters for a specific decade was also observed in [121].

Regardless of how potentially unorganized research in HCI might be, the benefits of its use are evident in several other research studies. The benefits of HCI solutions are the main focus of various SLR and SMS studies (e.g., [11,12,122]). For example, the authors in [12] investigated wearable devices, arguing that they have brought the highest level of convenience and assistance to people than ever before. The findings are supported by the study results conducted in [11], where authors addressed the benefits of wearable devices for the aging population with chronic diseases, potentially reducing the social and economic burdens. The importance of HCI in healthcare was furthermore analyzed in [13,122], where the research was focused on people with disabilities and related health problems. The role of AI technology for activity recognition, data processing, decision making, image recognition, prediction making, and voice recognition in smart home interactive solutions was also analyzed [17]. Some existing SLR studies are also focused on the general use of HCI solutions, providing support in healthcare [122], smart living [17], or understanding human emotions from speech [18].

The second focus of published SLR and SMS studies are sensors, signals, and the intelligent use of different devices. Authors in [12] investigated types of wearable devices for general users. At the same time, [13] addressed the benefits of ambient-assisted living and IUI for people with special needs, concluding how important it is to design user-friendly interfaces to provide an excellent HCI mechanism that fits the needs of all users. A similar effort was made in [123], only this time the general user population is included, indicating that several “general solutions” do not always have user-friendly interfaces. The level of intelligence is low, and they need to be improved.

The importance of well-designed and well-interpreted HCII can be presented as the third focus of studies. For example, in [37], authors recognized new HCI scenarios, such as smart homes and driverless cars. In [124], augmented reality (AR) and the third generation of AI technology are investigated. However, both studies lack a systematic review (a lower number of literature units indicate limited research space in these specific topics). The risk of misinterpretation of signals and the connecting risks are addressed in [15] (EMG) and in [16] (EEG), both claiming there are several issues in this area. The review of [15] focuses on deep learning in EMG decoding, while [16] is set to find different good practices within existing research. 

Benefits of IoT and HCI collaboration are addressed within the application of IoT systems, emphasizing the influence of human factors while using HCI [125]. The authors in [125] address the advantages of information visualization, cognition, and human trust in intelligent systems. In contrast, the authors in [126] present a unified framework for deriving and analyzing adaptive and scalable network design and resource allocation schemes for IoT.

In the last decade, there was an emphasis on developing solutions for mobile devices. SLRs related to the HCII field on mobile devices are focused on mobile emotion recognition methods, primarily but not exclusively addressing smartphone devices. The authors in [21] deliver a systematic overview of publications from the past ten years addressing smartphone emotion recognition, providing a detailed presentation of 75 studies. Meyer et al. [20] also analyzed the existing research field of mobile emotion measurement and recognition. By conducting a literature review, they were focused on optical emotion recognition or face recognition, acoustic emotion recognition or speech recognition, behavior-based emotion recognition or gesture recognition, and vital-data-based emotion recognition or biofeedback recognition. Research, conducted by Tzafilkou et al. [19] addressed the use of non-intrusive mobile sensing methodologies for emotion recognition in smartphone devices, narrowing the timescale and number of papers even more: 30 articles during the past six years. Similar to the findings of our study, the authors identified a peak of papers published in 2016 and 2017. Based on the results that revealed main research trends and gaps in the field, the authors discussed research challenges and considerations of practical implications for the design of emotion-aware systems within the context of distance education.

## 3. Materials and Methods

**Figure 2:**
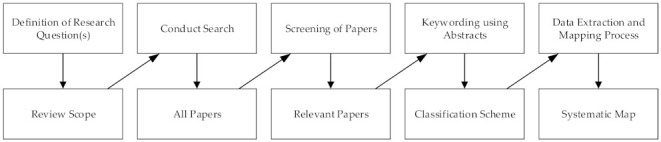
Systematic mapping study process adapted from [127].

We conducted a systematic mapping study (SMS) to identify state-of-the-art research in the HCII area and to observe trends in research and the developments in sensor technology and AI methods and algorithms for HCII. We followed the guidelines prepared by Pettersen et al. [127] (see Figure 2). 

**Figure 3:**
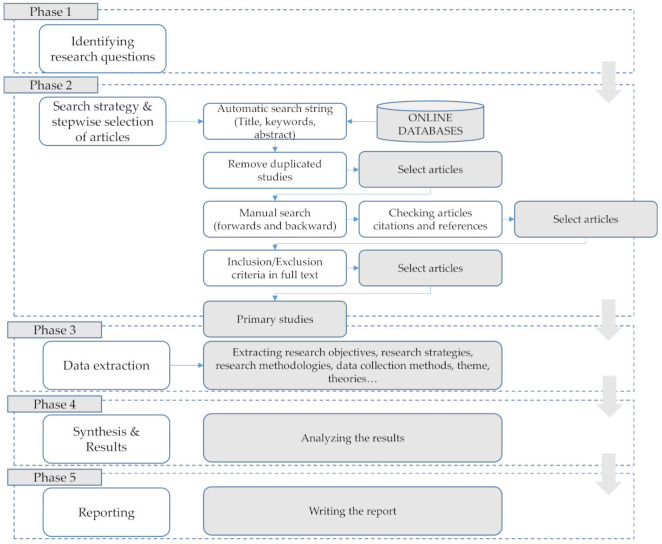
Research process map.

The research activities in this study were divided into five phases (see Figure 3). In the first phase, the research questions presented in Section 3.1 were defined. Next, the scope of the study was reviewed with the preliminary research in the selected databases, presented in Table 2. At this point, we conducted the review of related work and created the first draft of the classification scheme based on our research questions and propositions from related work. The search was conducted following the predefined inclusion and exclusion criteria presented in Table 3. After screening the papers based on abstract in the first step and whole content screening in the next step, the relevant papers were selected, and the draft classification scheme was revised and extended. The final version of the scheme is presented in Table 5. In phase three, data from the selected papers were extracted in accordance with the finalized classification scheme. In phase four, systematic maps presented in Section 4 were created, and the results were analyzed. Predefined steps for SMS were extended in our study, as we defined the draft classification scheme after reviewing the research scope. The research was concluded in phase 5 with writing activities.

### 3.1. Definition of Research Questions

The primary goal of this study is to identify, analyze, and synthesize existing work in the HCII field. The main objective of this study is to (1) systematically review relevant scientific articles to conduct a SMS of the HCII area and (2) to present trends and demographic analysis of the HCII research field. Based on the research goal, we formulated three main research questions—RQ1, RQ2, and RQ3. As the main questions are too general, a set of sub-research questions for all were proposed to provide more complete answers to the formulated research questions, as presented in Table 1. 

### 3.2. Conducting Search and Screening

After identifying the research questions, we defined the appropriate keywords for finding all published articles with topics from HCII and IUI. As we wanted to provide a comprehensive overview of the research area, broad keywords were used. The elementary search query string used for finding published articles was the following:

*“intelligent interaction” OR “intelligent user interface”.*

For finding the relevant literature, we used the following publicly established available digital libraries: ACM, IEEE, ScienceDirect, Scopus, and Web of Science. The first search, conducted using the digital libraries, yielded 5642 articles (see Table 2) that were used as inputs into the next selection process step. Several inclusion and exclusion criteria were applied (see the criteria specification in Table 3). 

**Figure 4:**
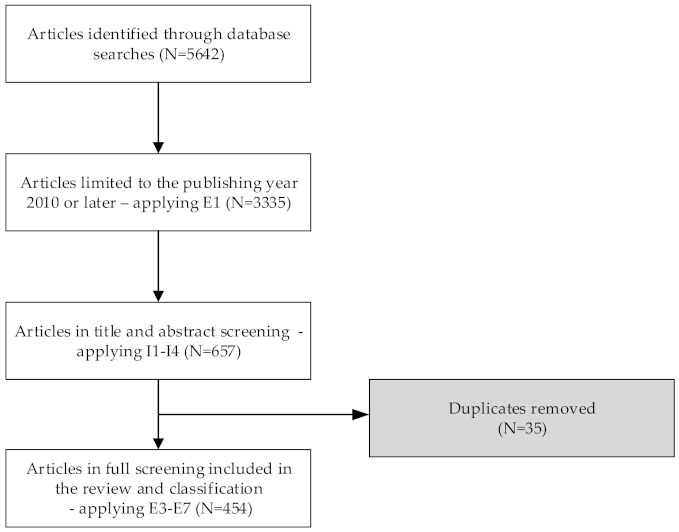
Flow diagram of the database searches and article screening process.

The process of selecting the relevant literature was carried out in several steps (see Table 4 and Figure 4). In the second step, we have limited the set of studies by applying the exclusion criteria E1 to include only studies published in 2010 or later, which resulted in a set of 3335 studies. The titles and abstracts were read to select studies that address intelligent interaction or intelligent user interfaces in step three. In the third step, we also excluded studies that were not published in English and were not accessible electronically. The third step provided 657 articles published in a journal, conference proceeding, or a book as a book section. In the next step, removing 35 duplicate entries resulted in 622 articles used for the last step of the selection process. In the fifth step, the exclusion criteria E3–E7 were applied to exclude studies unrelated to computer science and HCI research areas. In addition, literate reviews or systemic mapping studies were excluded. Short papers with less than four pages and articles not written in English were not selected for the final analysis. Although the language criteria were already applied in step three, some articles we found had titles and abstracts written English; however, the rest of the article was not written in English. The last screening step resulted in 454 articles that were used for the systematic mapping study.

### 3.3. Classification and Data Extraction

To ensure that all studies would be analyzed consistently, rules about coding data about study characteristics and the results were specified. A predefined classification scheme is summarized in the Table 5. Firstly, we noted the article type (EC1) as a journal article, conference paper, or book selection. Proceedings papers were classified as conference papers. In terms of research type (EC2), a study was classified as quantitative when authors used quantitative methods for data analysis, qualitative if qualitative methods were used for data analysis, and mixed for studies where quantitative and qualitative data analysis methods were used. Research method type (EC3) was observed as suggested by [128] as validation research, evaluation research, a solution proposal, a philosophical paper, an opinions paper, or an experience paper. Validation research—a certain novel HCII method/technique/algorithm/tool, which has not yet been implemented in practice and was validated using a method-like case study, and experiments in lab, simulation, prototyping, etc. Evaluation research—a certain HCII method/technique/algorithm/tool, which was implemented and evaluated in practice. It shows how the method/technique/algorithm/tool was implemented in practice and what the benefits and drawbacks of the implementation are, evaluated using techniques, such as industrial case study, controlled experiments with practitioners, action research, etc. Solution proposal—a HCII method/technique/algorithm/tool is proposed, which can be either novel or a significant extension of an existing one. The potential benefits and the applicability of the solution is shown by a small example or a good line of argumentation. However, the proposed solution has not yet been implemented. Philosophical papers—for better understanding of existing things, a new-structured view is provided, by constructing a taxonomy or conceptual framework. Opinion papers—a personal opinion is provided of somebody, about whether a certain HCII method/technique/algorithm/tool is good or bad, or how things should be done. The opinions usually do not rely on related work and/or research methodologies. Experience papers—papers explain the author’s personal experience on what and how HCII method/technique/algorithm/tool has been done in practice. The research strategy was noted with EC4 as a case study, experiment, survey, grounded theory, user study, field study, mixed study, exploratory study, or literature review. As the IUI is a multidisciplinary filed, we noted the predominant research standpoint with EC6 as accessible UI, adaptive UI, artificial intelligence, brain computer interface (BCI), human–computer interaction (HCI), human–machine interaction (HMI), intelligent interaction (II), or intelligent UI. The research standpoint values were gathered from related literature [7] and extended during the screening. The list of values for data source (EC10), sensor type (EC11), and AI methods used (EC12) were defined literature (one source was for example [13]) and extended during the screening process.

## 4. Results

This section presents the results obtained from the analysis of the 454 primary studies. The objective of this study was to identify and systematically map the research in the field of HCII and IUI.

### 4.1. Trends and Demographics of the Literature within the Field of HCII

**Figure 5:**
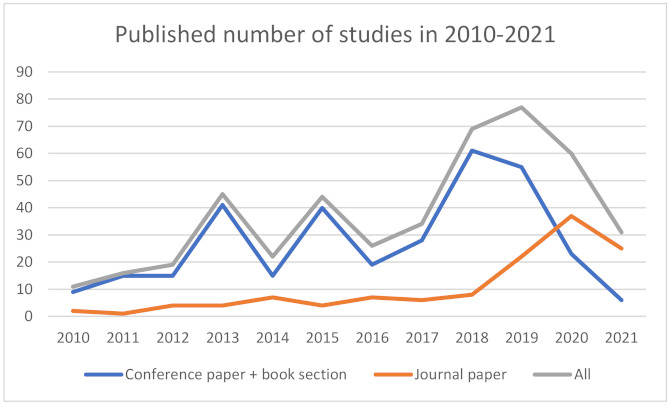
Number of studies published through years 2010–2021 (all = 454).

**Figure 6:**
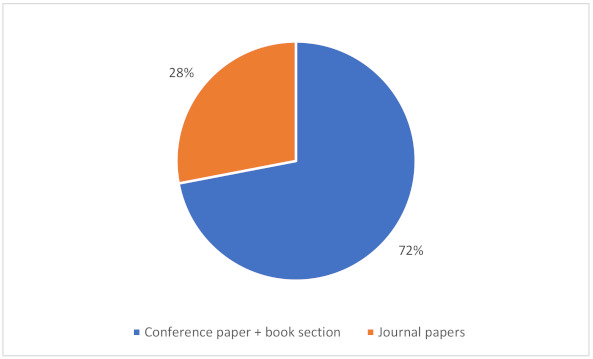
Percentage of papers based on publication type (all = 454).

As illustrated in Figure 5, the number of studies has been increasing in the last decade. The number of primary studies has slightly decreased in 2014 and 2016. Otherwise, a clear trend of increased interest in observed fields can be reported. Along with the increasing number of primary studies in recent years, we also noticed the change in the article types; a positive trend in the number of journal papers compared to other primary research types can be observed since 2017. The data for 2021 is incomplete as the data search was concluded in October 2021. As shown in Figure 6, a majority (72%) of the HCII and IUI articles have been published in conference proceedings or as book sections. 

We ranked the journals and conferences by the number of primary articles published to get a broader view of top venues for the HCII and IUI literature. Results presented in Table 6 indicate that HCII and IUI research articles could be found in a broad spectrum of journals. Most primary studies have been published in *IEEE Access* (21), followed by *Multimedia Tools and Applications* (9), *Expert Systems with Applications* (6), *IEEE Transactions on Affective Computing* (5), and *Procedia Computer Science* (5). The conference with the highest number of proceedings papers on HCII and IUI topics is the International Conference on Affective Computing and Intelligent Interaction (ACII), where 16% of the observed proceeding papers were published. A further 24 proceedings papers were published in Humaine Association Conference on Affective Computing and Intelligent Interaction, and further 15 were published in the Asian Conference on Affective Computing and Intelligent Interaction (ACII Asia).

Table 7 lists the top-cited primary studies in HCII and IUI identified and analyzed in this systematic mapping study. Our sample’s most cited primary research was the journal article “Analysis of EEG Signals” and “Facial Expressions for Continuous Emotion Detection” [129], with 219 citations. Further analysis demonstrated that the five most cited articles focused on emotion or stress recognition from different data sources (EEG signals, wearable sensors, audio, or smartphones). In the last decade, the mean number of citations for observed studies in the HCII and IUI research fields was 8.7. On average, observed conference proceedings papers in our sample were cited 6.1 times, book chapters were cited 11.5 times, while observed journal papers on average have 15.1 citations. Out of the ten top-cited studies, most (N = 7) were journal papers, while the other three were conference proceedings.

Analyzing the impact of a specific study in the existing literature, only from the point of view of the number of all citations, may not be the best, especially if we want to compare studies that have been published ten years apart. Articles published several years ago have probably had a greater chance of being recognized and cited by other authors. Moreover, for studies that have been conducted and published recently, there may not have been enough time for them to be already recognized and cited in the existing literature by other authors. Therefore, to provide an additional view on the most influential research in HCII, we ranked the studies according to the average number of paper citations per year (see Table 8). Based on the average number of citations per year, this time, the study EmotionMeter: A Multimodal Framework for Recognizing Human Emotions [112], performed best with 62,67 citations on average per year. The second best ranked was the study Analysis of EEG Signals and Facial Expressions for Continuous Emotion Detection [129], with 36.5 citations per year. The study ranked third was Deep learning analysis of mobile physiological, environmental, and location sensor data for emotion detection [131], with 34 citations per year on average.

To get a broader view of the contributors in the research field, who scientifically promote HCII and IUI topics, we have observed the country of the author’s affiliation. As shown in Table 9, most of the authors active in HCII and IUI field are primarily located in China (28%), followed by the USA (13%), India (10%), United Kingdom (7%), Germany (5%), Japan (4%) and South Korea (4%). We observe that most of the field research is conducted by researchers from Asia (leading with China, India, Japan, and South Korea), Northern America, Europe (leading with UK, Germany, Italy and followed by Portugal, Spain, France, and Belgium), and Australia.

### 4.2. The Research Space of the Literature within the Field of HCII in the Last Decade

**Figure 7:**
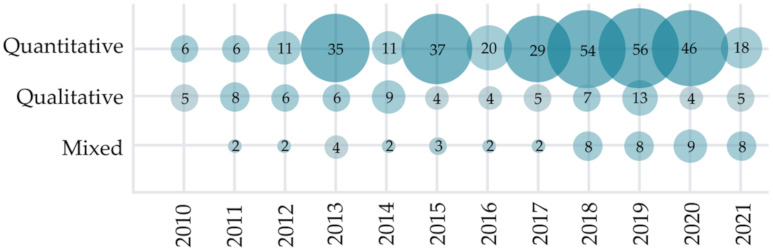
Number of studies by year and research type.

To address the RQ2.1, we categorized the primary literature by the type of research as quantitative, qualitative, or mixed. Qualitative research is broadly accepted in the HCI and IUI field, as the research goal can be focused on understanding the subjectivity, not measuring and manipulating the objective data. Nevertheless, as shown in Figure 7, most of the analyzed research (72% of studies) from HCII and IUI fields is quantitative. Qualitative and mixed research is used less often, in 16% and 11% of analyzed papers, respectively. The trend of prevailing quantitative research type is consistent over the years, with most of the primary research being quantitative in all of the observed years in the last decade. However, we noticed a slight increase in studies using mixed research types in recent years.

**Figure 8:**
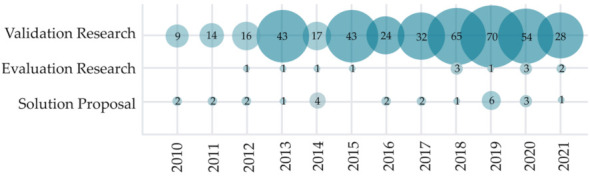
Number of studies by year and research methodology.

Research type classification per publication year is visualized in Figure 8. As illustrated, the vast majority (91.5%) of the observed primary studies were categorized as validation research. Further 5.7% of included studies were categorized as evaluation research proposals, and 2.8% were solution proposals. The results indicate a strong trend of researchers in the HCII and IUI field publishing finalized ideas and solutions.

**Figure 9:**
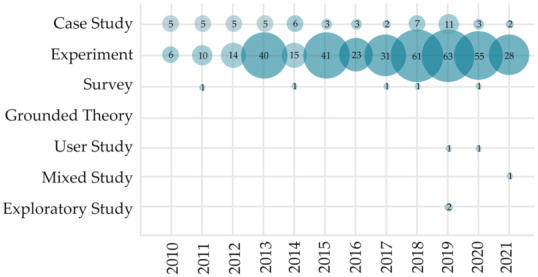
Number of studies by year and research method type.

To further analyze how the research is conducted in the HCII and IUI studies, we noted the methodology used to validate or evaluate the proposed solutions in the primary studies. Results displayed in Figure 9 show that the most used method in observed studies is an experiment (observed in 387 articles), followed by a case study (57 studies). Only a few user studies (N = 2), mixed studies (N = 1), and exploratory studies (N = 2) were found in our sample, all of which have been conducted in the last two years. In the last decade, five survey studies have been performed, evenly spread through the observed years.

**Figure 10:**
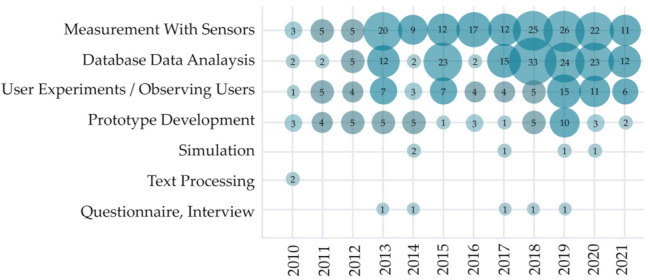
Number of studies by year and data collection method.

To achieve a clear signal of the user’s emotional state, HCII and IUI solutions use different methods for collecting the data. An overview of data collection methods used in observed research is visualized in Figure 10. The most popular methods for data collection in the last decade have been measurement with sensors (167 studies) and database data analysis (155 studies). The prevalent use of these two methods can be noted in most of the observed years, except for 2014 and 2016, when slightly more studies used user experiments and prototype development for data collection. User experiments or observing studies and prototype development have been used in 72 and 47 observed studies, respectively, while other methods have overall been used more seldom; simulation has been used for data collection in six studies, questionnaire, or interview in five studies, and text processing in two studies.

**Figure 11:**
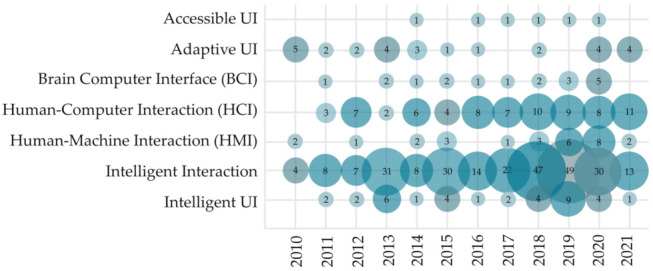
Number of studies by year and research standpoint.

The observed research point is multi-disciplinary, interchanging the ideas from different areas. As visualized in Figure 11, most of the primary research articles (263 articles) were conducted from intelligent interaction, with the trend continuing through the last decade. A further 75 studies were written from a human–computer interaction (HCI) standpoint and a further 36 from an intelligent UI standpoint. HCII and IUI research field was less frequently explored from the standpoint of adaptive UI (28 studies), human–machine interaction (HMI) (28 studies), brain–computer interface (BCI), and accessible UI (6 studies). Nevertheless, we can observe a slight increase of research articles written from the standpoint of human–machine interaction (HMI) and intelligent UI since 2018.

**Figure 12:**
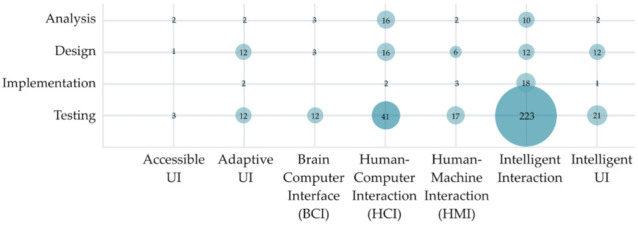
Distribution of HCII solutions’ development phases according to the research standpoint.

Figure 12 shows which research topics (focus area) have been investigated in HCII and IUI research classified into various development phases. Most of the proposed solutions in the HCII and IUI fields are already in the testing phase (329 studies). Further, 26 proposed solutions are in the implementation phase, 62 propositions were shared in the design phase, and 37 solutions were published in the analysis phase. The visualization clearly indicates the trend of publishing finalized and at least partially tested solutions in the field of HCII and IUI.

### 4.3. Sensors Technology and Intelligent Methods in the Development and Evaluation of HCII Solutions

**Figure 13:**
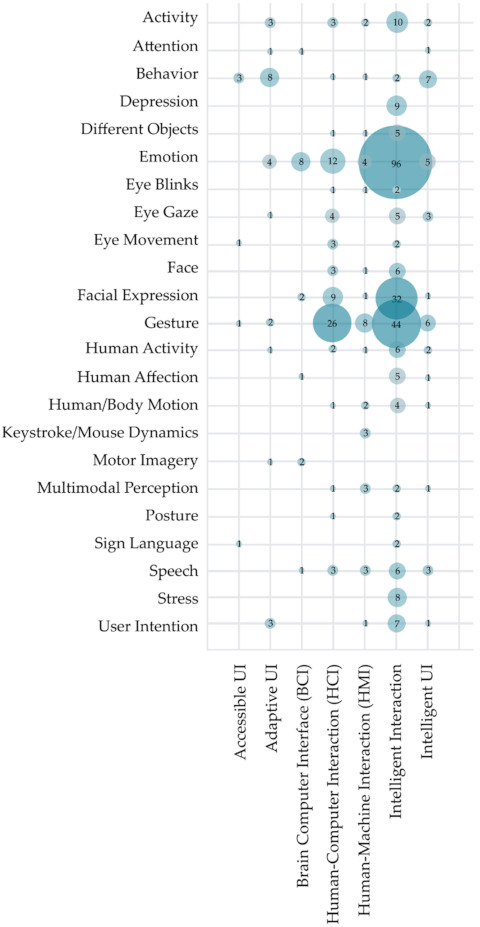
Distribution of HCI recognition solutions according to the research standpoint.

Figure 13 shows the correlation between the main aim of intelligent recognition and the standpoint of the research. As we have already discussed in previous sections, most of the analyzed research has been conducted from an intelligent interaction standpoint. As shown in Figure 13, this research is mostly focused on emotion recognition (96 primary studies), gesture recognition (44 studies), and facial expression recognition (32 studies). A similar trend in recognition focus can be observed in papers from other standpoints as well. Overall, analyzed studies from the HCII and IUI fields have primarily focused on intelligent recognition of emotion (129 studies), gestures (87 studies), and facial expressions (45 studies). A slightly different trend can be visible in the research from an intelligent UI and adaptive UI standpoint, where we can observe a focus on behavior recognition along with the previously mentioned recognition trends.

**Figure 14:**
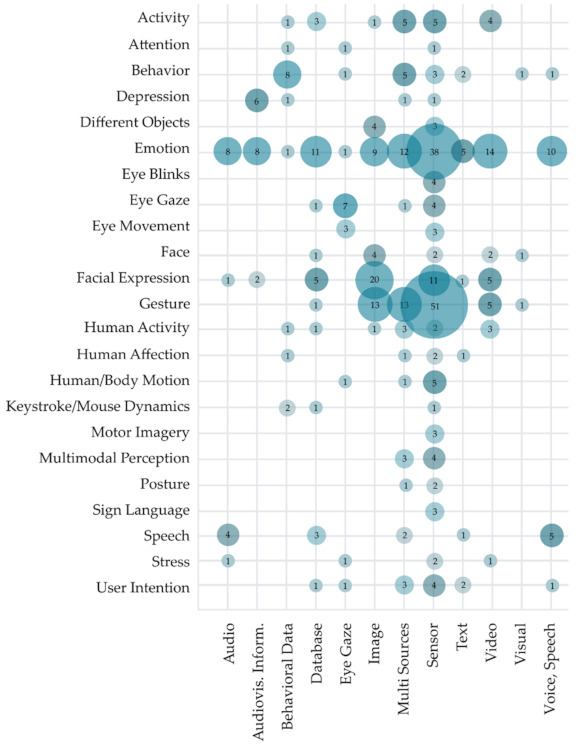
Distribution of data sources in existing HCI recognition solutions.

Visualization of analyzed studies based on the primary data source used for evaluating the proposed solutions of the HCII and the main aim of the intelligent recognition is presented in Figure 14. The use of sensor data (165 studies), images (56 studies), and use of multiple sources (51 studies) are overall the most used data sources. However, we can observe that studies focusing on intelligent speech recognition most commonly use voice and speech (five studies), audio (four studies), or database as data sources. Studies focused on eye movement and gaze recognition most often use eye gaze as the data source, while studies focused on emotion recognition mostly use video sources. The difference in used data sources can also be observed in studies focused on the recognition of depression. They most often use audiovisual information, as the data source and in papers focused on behavior recognition, which most commonly use behavior as the data source. As visualized in Figure 14, the research interest in the HCII solutions is highly focused on emotion recognition based on multi-source or sensor data and gesture recognition based on the same data sources. Although some combination of data sources and aim of intelligent recognition is nonsensical, we still observe some research opportunities, especially in using different data sources to recognize human affection, attention, depression, and sign language.

**Figure 15:**
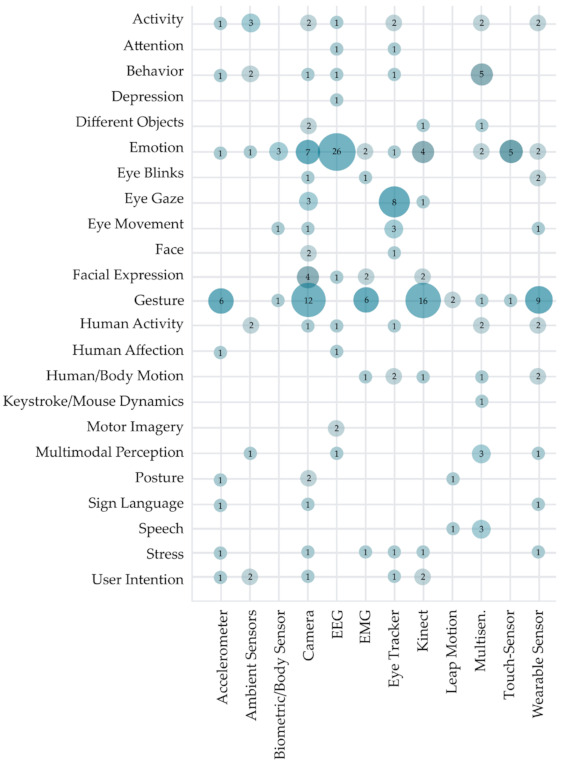
Distribution of sensors in existing HCI recognition solutions.

Figure 15 addresses RQ3.1 and RQ3.3, showing the distribution of primary studies based on intelligent recognition’s aim and used sensor type. Half of the observed studies (52%) used sensors to obtain the data. The most widely used sensor types in the observed primary research were camera (48 studies), EEG sensor (37 studies), and Kinect (used in 30 studies), followed by wearable sensors (23 studies), eye tracker (22 studies), and multisensor (21 studies). Obtained data was in most cases used for the recognition of emotion (54 studies) and gesture recognition (54 studies). We can observe some research possibilities in using biometric/body sensors as data sources. They were only used in five studies (2% of all observed studies used sensors to obtain data). Seldom use of touch-frame/touch screen sensors (six studies) for data gathering was also surprising due to their affordability and overall widespread use. Some combinations of observed sensors and recognition solutions in Figure 15 are illogical (e.g., an accelerometer was not used for eye blinks and movement recognition) and do not indicate research gaps in the observed field.

**Figure 16:**
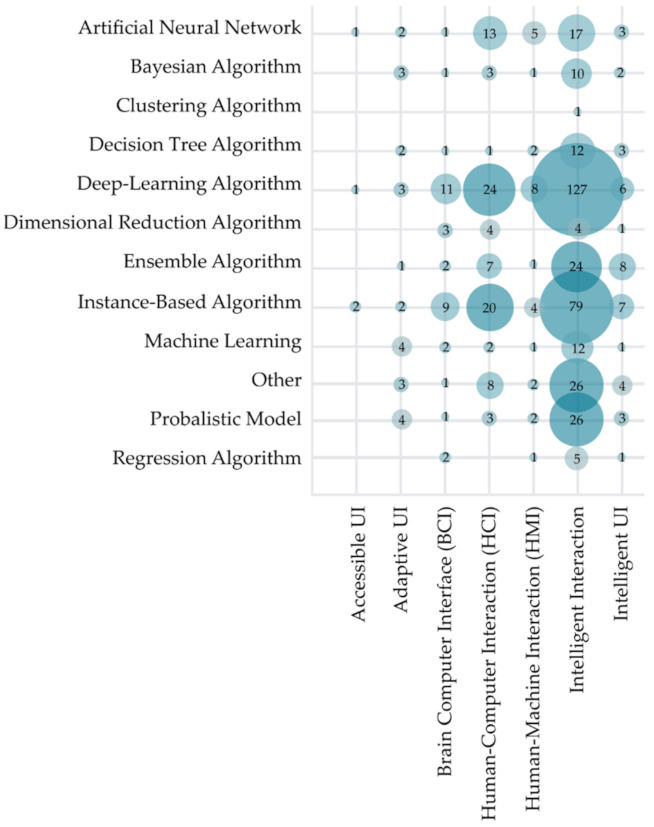
Distribution of AI methods and algorithms according to the research standpoint.

To address RQ3.4, AI methods used in the analyzed research were visualized in Figure 16 regarding the research standpoint. Note that some studies used multiple different methods of artificial intelligence. Therefore, the sum of used methods is larger than the number of included studies (N = 556). It is visible that the most used AI methods are deep-learning algorithms (178 studies) and instance-based algorithms (123 studies). Further observation of methods used concerning research standpoint shows that deep-learning algorithms are most commonly used without regard of the research standpoint. An exception was observed for adaptive UI, where researchers most commonly used the probabilistic model (35% studies from the adaptive UI standpoint), and for intelligent UI, where ensemble algorithm was used in 50% of studies. Widespread use of deep learning could be attributed to its effective extraction of robust and salient features from various datasets.

**Figure 17:**
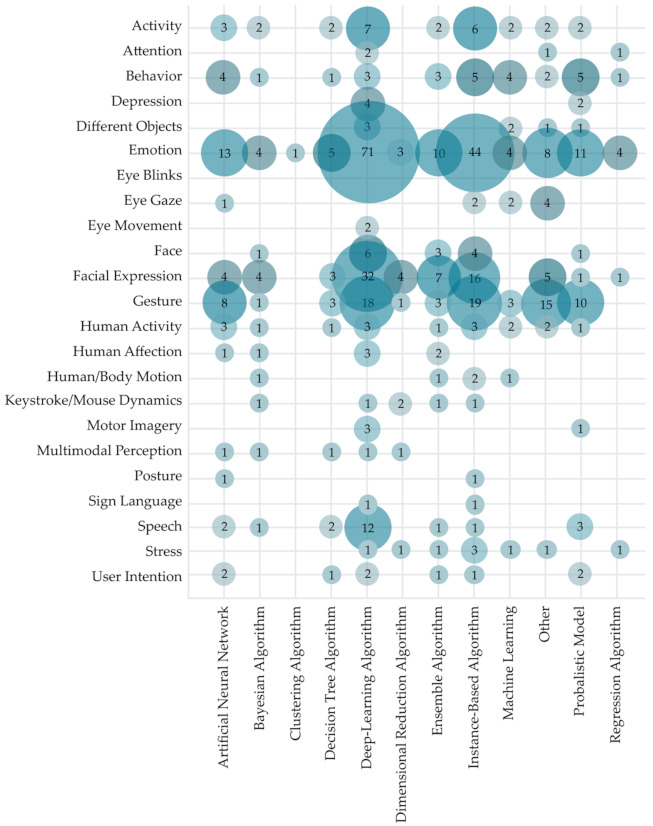
Distribution of AI methods and algorithms for HCI recognition solutions.

To further investigate the use of AI in the HCII and IUI field, an analysis of the used artificial intelligence method concerning the HCI recognition’s aim is visualized in Figure 17. As expected, deep-learning algorithms were widely used in most categories of recognition. However, instance-based algorithms were most widely used with the aim of human/body motion recognition, human activity, gesture, depression, and behavior recognition. The use of artificial neural networks should also be mentioned (included in 47 studies), as it was successfully used in gesture (eight studies) and emotion recognition (13 studies). Further, 41 studies have used probabilistic models for the same aim of recognition.

**Figure 18:**
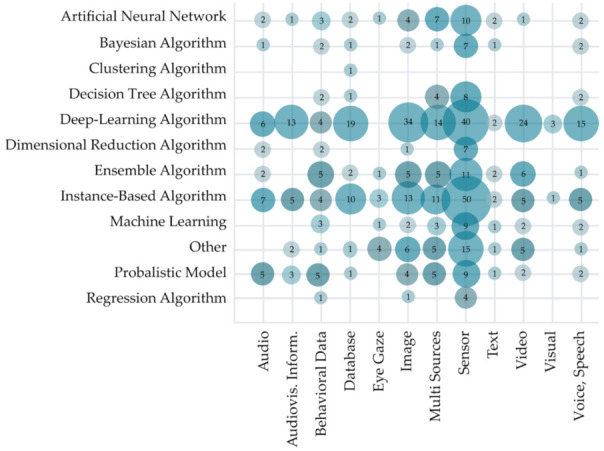
Distribution of data sources used in AI methods and algorithms.

The visualization of used AI method concerning used data type in Figure 18 reveals some interesting practical implications for using different AI methods for various data types. As previously observed, the use of sensor data are most common in the HCII and IUI solutions (used in 174 studies), with the most common AI methods for its processing being instance-based algorithms (used in 50 studies) and deep-learning algorithms (40 studies). Deep-learning algorithms are widely used to process audiovisual information (13 studies) and database data (19 studies). Instance-based algorithms are also commonly used to process audio (seven studies) and image data (13 studies). In contrast to this trend, behavior data were in most cases processed with probabilistic models (six studies) and ensemble algorithm (five studies), while the eye gaze data were most widely analyzed with the help of other AI methods. Note that all the AI methods used in the studies are presented in this visualization. Therefore, the total sum of instances (N = 151) in Figure 18 is higher than the total number of analyzed primary studies, in which this metric could be observed (N = 454). Most of the primary studies (315 studies) used a single AI method, further 97 of studies used two methods of artificial intelligence in their research, 26 studies used three different AI methods, 13 studies used four and a further four of the observed primary studies used six different AI methods.

**Figure 19:**
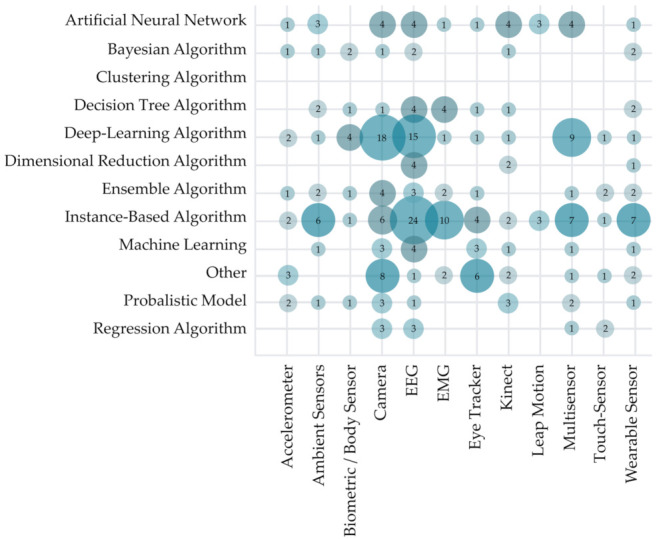
Distribution of sensors used in AI methods and algorithms.

Further analysis of AI method types used in HCII and IUI research concerning the sensors used for data collection is presented in Figure 19. Instance-based algorithms were used in the majority of the studies (N = 73). The method was most widely used in the research using data obtained from EEG sensors (24 studies), EMG sensors (10 studies), ambient sensors (six studies), wearable sensors (seven studies) and Leap motion (three sensors). Deep-learning algorithms were also widely used (54 papers). The method was dominantly used in studies with data acquired using cameras (18 papers), EEG sensor (15 studies), multisensors (nine studies), and biometric/body sensors (four studies). In contrast, an artificial neural network was most commonly used in the studies, using cameras (four studies), EEG (four studies), Kinect (four studies), ambient sensors (three studies), and Leap motion (three studies).

**Figure 20:**
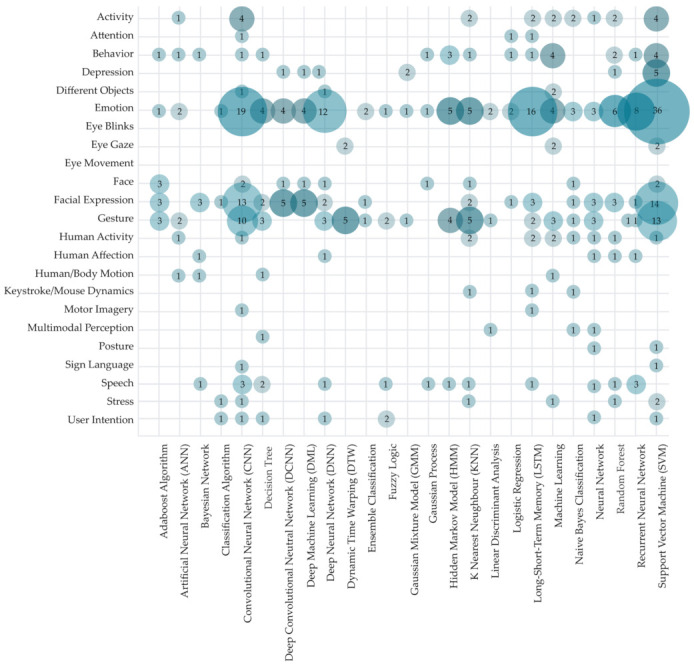
Distribution of AI methods and algorithms HCI recognition used for HCI recognition solutions.

To provide an in-depth overview of the HCII field, a systematic map including the aim of HCI recognition and used AI methods and/or algorithms is presented in Figure 20. Visualization includes only studies where both the aim of HCI recognition and AI methods and/or algorithms could be classified (N = 421). SVM is the most widely used algorithm for various kinds of recognition (used in 87 studies), primarily emotion recognition (36 studies), facial expression recognition (14 studies), and gesture recognition (13 studies). We can also report the widespread use of CNN, which was used in 62 of the observed primary studies, mainly for emotion recognition (19 studies), facial recognition (13 studies), and gesture recognition (10 studies). As mentioned before, emotion recognition was the aim of almost a quarter of observed studies (141 studies), followed by studies working on gesture recognition (65 studies) and facial expression recognition (59 studies). Researchers have used a wide range of AI methods and algorithms for emotion recognition. Of all the observed methods and algorithms, only classification algorithms and dynamic time warping have not been used in the primary research with the aim of emotion recognition. However, the challenges of emotion recognition were most often approached with SVM, CNN, LSTM, and deep neural network (DNN). Studies focused on gesture and facial expression recognition have covered most of the observed AI approaches except for ANN, ensemble classification, FL, GMM, GP, HMM, LDA, and ML. The most used method in studies of facial expression recognition was CNN and SVM. Analyzed HCII research with the aim of gesture recognition has not covered BN, classification algorithm, CNN, deep ML, GP, LoR, and RF. In terms of the mapping results presented in Figure 19, it can be observed that most research, divided by the aim of HCI recognition, cuts across different AI methods and algorithms. For example, HCI research focused on recognizing stress was less researched (seven studies) though analyzed with the classification algorithm, NN, k-NN, ML, RF, and SVM.

## 5. Discussion

While the existing body of research proves there is already a vast plethora of HCI-related research, research in the field of HCII and IUI has continued to grow even more in the last decade. However, there is confusion present among research classification and often misinterpretation of what is an intelligent user interface, what intelligent HCI is, and how to classify them. Therefore, in this paper, we present the results from a systematic mapping study in HCII and intelligent IUI, including a total of 454 articles, aiming to classify current research trends in the field. This paper summarized insights into the state of the art with the aims of (1) understanding the research standpoints present in this multidisciplinary field, (2) highlighting open issues that need to be filled by future research, and (3) identifying the use of AI methods and algorithms for categorized HCII solutions, aiming to achieve recognition from different data sources.

Our study highlights how researchers currently aim to develop innovative contributions in terms of type and phase of research, the aim of the recognition, used data sources and used AI methods and algorithms. Our study identified existing research from the HCII and IUI fields and investigated trends related to sensors technology, AI methods, or algorithms. Based on the analysis of the existing HCII in IUI-related research, this study provided theoretical and practical implications, an overview of the most used methods and algorithms in different HCII solutions, and under-researched topics.

### 5.1. Theoretical and Practical Implications

Based on the data from 454 research papers published between 2010 and 2021, several theoretical and practical implications were identified, affecting the course of our future research, and possibly the study focus of other researchers. Numerous research papers claim that intelligent solutions and sensors are becoming progressively crucial for users in their everyday lives, facilitating a higher level of living in the domain of health, entertainment, economics, and general smart living. Literature suggests that AI and IUIs will be part of our daily lives. Our needs will heavily depend on them, creating a necessity to equip the user interfaces with human communication skills and enable natural interaction between the computer and a human to the highest possible extent. Therefore, future efforts should be invested in improving emotion, gesture, and facial recognition of intelligent interfaces, and in HCI in general.

Theoretical implications interesting for researchers are therefore recognized in research gaps illustrated in this study. Some topics, such as accessible UI and sign language recognition, have received little attention and should be considered for future research possibilities. Topics on the brain–computer interface are comparatively still sparsely covered, though they have been gaining popularity in the last decade, with the number of associated studies rising each year. With the detailed overview of the research standpoints, we have illustrated their overlap in selected topics, which could hopefully spark collaboration initiatives from active researchers with various research standpoints that this interdisciplinary field joins. This mapping study confirms that the HCII and IUI field is an emerging field, as the latest few years have been the most productive in the last decade. Our overview also points out the methodological gap in qualitative research studies, which are otherwise broadly accepted in HCI. There appears to be a lack of focus (partly covered by little experiments) on how the end-users accept the suggested intelligent solutions outside of the controlled environment. Lack of validation proposals can also be observed in the low total number of conducted user studies (n = 2). As great efforts have been made to the technical advances of the intelligent solutions, our study also serves to remind the lack of philosophical studies focused on the ethical limitations and aspects future HCII solutions ought to consider. 

Practical implications of this research result in several recommendations and support the theoretical implications. The most cited papers focused on emotion and stress recognition, creating the central field of study, largely present in other related studies. In addition, intelligent interaction is frequently connected with emotion recognition, followed by gesture recognition. Emotion and gesture recognition is the most comprehensive data source in sensors, mostly retrieved by EEG and Kinect, creating the foreground of HCI research. 

Since quantitative research is more prevalent than qualitative, a lack of research in this field creates a window of opportunity for researchers, especially looking for advancements for users with special needs, focusing on progress inaccessible and adaptive UI. The sensors used in the research mainly were applied to identify gestures and emotion, followed by using existing images to identify and understand facial expressions, again creating the most exciting field of research and directing the course of HCII research in the future. 

In HCI publishing, there is a trend of research presentation in conferences and less in journals, indicating several general presentations of HCI solutions, lacking the in-depth (and time-consuming) analysis typical for journal papers. The decline in the number of research after 2017 is arguably not consistent with fast progress achieved in HCI development, creating an opportunity to conduct additional research in the field of the newest HCI application. Practitioners can benefit from our overview of the used AI methods and algorithms, already associated with various aims of HCI recognition, which are presented in Section 4.3. The findings on the connection of the associated sensor types and HCI recognition and associated sensor types and AI methods are also aimed to provide practical benefit in the development process of future HCII solutions, as they offer an overview of tried and tested combinations.

### 5.2. Limitations

Investigation of the sensor’s technology and AI methods and algorithms for HCII comes with a set of limitations. Although several digital libraries were used and three researchers were involved in the paper analysis, it is still possible that not all articles were identified and consequently included in the group of selected papers after inclusion and exclusion criteria. Furthermore, the used keywords in research papers varied greatly and consequently covered various multi-disciplinary research. Due to the un-uniform set of used keywords, the probability that articles addressing HCI or HCII topics, but not including the keywords used for the literature search, was not included in our analysis. 

The time scope of research was set after the year 2010. Although not much relevant research was conducted before the set period (based on our research), there could possibly exist fundamental papers with significant insight into the beginnings of HCII, but these were not included in our research. 

Articles, which were in the reviewing or publishing process when writing this manuscript, were not yet accessible in the used digital libraries and were therefore not included in the research. There is a possibility that after the conclusion of this paper, new important articles will emerge in 2021. 

A high number of identified potential papers was manually evaluated as appropriate or not appropriate for further use. The time restriction of a few months created the likelihood that not all relevant articles identified through the database searches (N = 5642) met the criteria set in the full screening process and resulting in 454 papers. Few important papers are possible to be mistakenly omitted from the final paper group. 

This study focuses on intelligent HCII and IUI and cannot be generalized to other domains, such as the general HCI. The exclusion of acronyms of the selected terms in the search query (HCII and IUI) could also be recognized as a limitation. This study represents an overview of the observed field and does not aim to contribute to the debate of what solutions are deemed intelligent in the HCII and IUI fields.

### 5.3. Threats to Validity

It is possible that not all relevant primary studies were covered in this study. Our work was guided by inclusion and exclusion criteria within the scope of the reported search string; therefore, it is possible that some articles were automatically excluded in the process. Some relevant studies were published after our literature search phase and are not included in our review. There is a possibility that the research gaps with low publication coverage we have identified have been covered in other sources, and our work is not a relevant summary of the whole population of relevant studies. However, we have examined similar studies and have explored all relevant databases to lessen these threats to gather a good and unbiased population representation. We believe the sample size is adequate and assures reasonable validity of the results.

Data extraction and classification of primary studies have been challenging due to the interdisciplinary characteristics of the field. Some level of subjectivity is implied in classification; therefore, it is possible that if the study is repeated, minor variations could be expected. For this reason, we have attached the classification of studies for selected variables in the Supplementary Materials. To minimize the threats to validity, primary studies were classified by one researcher. This might inherently introduce some bias, but we felt that the resulting classification would be more consistent this way. A predefined classification scheme was used to lower the risks to internal validity related to the data classification phase, and the authors decided to discuss the problematic papers.

## 6. Conclusions

This study contributes to the body of research in the multi-disciplinary field of intelligent methods development in HCI and HCII, CS, and AI fields. Our work presents a systematic mapping study of 454 papers, retrieved from several digital libraries and application of the inclusion and exclusion criteria, in order to fulfill the objectives of the study: (1) what have been the trends and demographics of the literature within the field of HCII, (2) what has been the research space of the literature within the field of HCII, and (3) what sensors technology and intelligent methods have been used in the development and evaluation of solutions for HCII. 

Our analysis revealed the following results. HCI and HCII solutions are becoming more and more popular and bring several conveniences in people’s daily lives. There is an extensively large body of research, with the peak reached in 2017.

The observed research point is multi-disciplinary, interchanging the ideas from different areas. Most of the analyzed research is quantitative. The most used method in observed studies is an experiment. The most popular methods for data collection are measurement with sensors and database data analysis. The most widely used sensor types in the observed primary research were a camera, EEG sensor, and Kinect, followed by wearable sensors, eye trackers, and others. The most used AI methods are deep-learning algorithms (widely used in various types of recognition) and instance-based algorithms, commonly used with the aim of human/body motion recognition, human activity, gesture, depression, and behavior recognition. The use of artificial neural networks was also identified, successfully used in gesture and emotion recognition.

In the future, we envision the following opportunities: There is a lack of philosophical papers dealing with the ethical aspect of intelligent recognition from various data sources, which is currently technically solved. The additional future work opportunity is increasing the inclusion of research in accessibility and using several different data sources to enable effective and efficient HCI for users with disabilities. We observed a lack in use-case studies and qualitative research, which for the HCII field present opportunity for future research, especially for studies evaluating the usability of proposed solutions and the user’s experience.

## Figures and Tables

**Table 1:** Research questions.

	Research Question
RQ1	What have been trends and demographics of the literature within the field of HCII? For the first main research question, the following set of research sub-questions were formulated:
RQ1.1	What is the annual number of publications in HCII field? (Publication count by year).
RQ1.2	Which studies in the HCII are most cited? (Top-cited studies).
RQ1.3	Which countries are contributing the most to the HCII field, based on the affiliations of the researchers? (Active countries).
RQ1.4	Which venues (i.e., journals, conferences) are the main targets of articles in the HCII field, measured by the number of published articles? (Top venues).
RQ2	What has been research space of the literature within the field of HCII in the last decade? For the second main research question, following sub-questions were formulated:
RQ2.1	What type of research is conducted in the HCII field? (Research type, e.g., quantitative, qualitative, and mixed).
RQ2.2	What type of research methods have been conducted in the HCII studies? (research method type, e.g., [127,128]: validation research, evaluation research, solution proposal, philosophical papers, opinion papers, and experience papers).
RQ 2.3	What research methodology is used for validating or evaluating the proposed HCII solution? (Research methodology, e.g., experiment, case study, etc.).
RQ 2.4	What are the common data collection methods in the HCII studies? (Data collection method, e.g., measurement with sensors, questionnaires, observing users, image processing, etc.).
RG2.5	What is the main standpoint of the research studies? *(Research standpoint, e.g.,* *HCII, IUI, II, Adaptive UI, etc.).*
RQ2.6	What phase of the HCII development and evaluation are presented in existing studies (analysis, design, implementation, and testing) (HCII development phase).
RQ3	What sensors technology and intelligent methods and algorithms have been used in the development and evaluation of solutions for HCII? For the third main research question, following sub-questions were formulated:
RQ3.1	What is the main aim of the intelligent recognition? (Recognition-of, e.g., emotion, gesture, etc.).
RQ3.2	What is the main data source for the evaluation of the proposed solution of the HCII? (Data source, e.g., audio signal, audiovisual information, sensor, etc.).
RQ3.3	What type of the sensor was used in the studies? (Sensor-type, e.g., camera, Kinect, etc.).
RQ3.4	What AI method and algorithms were used? (AI-methods and algorithms used, e.g., ANN, CNN, etc.).

**Table 2:** Articles retrieved from the selected digital libraries using the specified search string.

Database	Nr. of Articles
ACM	889
IEEE	1488
MDPI	46
Science Direct	1449
Scopus	1395
Web of Science	421
Together	5642

**Table 3:** Inclusion and exclusion criteria.

	Criteria	Description
I1	Field	Include studies addressing intelligent interaction or intelligent user interfaces.
I2	Language	The article must be written in English.
I3	Availability	The article must be accessible electronically.
I4	Literature type	Include articles published in peer-reviewed journals, conference proceedings, or a book (e.g., lecture notes).
E1	Year	Exclude literature, published before the year 2010.
E2	Duplicates	Exclude any duplicated studies found in multiple databases.
E3	Research area	Exclude non-computer science or non-human–computer interaction literature.
E4	Methodology type	Exclude articles that report results of a systematic literature review or systematic mapping study.
E5	Language	Exclude articles not written in English.
E6	Field	Exclude studies outside of the scope of HCII or II.
E7		Exclude articles less than four pages long that do not provide enough information about the study conducted.

**Table 4:** Steps in screening and selection of the relevant literature.

Step	Activity	Nr. of Articles
I	Automatic search in digital libraries	5642
II	Applying E1	3335
III	Screening by title and abstract (applying I1-I4)	657
IV	Applying E6 (removing the duplicates)	622
V	Screening with fast reading the manuscript (applying E3-E7)	454

**Table 5:** Coding and classification scheme.

	Variable	Description
EC1	Article type	Journal article, conference paper, book section
EC2	Research type	Quantitative, qualitative, mixed
EC3	Research method type	Validation research, evaluation research, solution proposal, Philosophical paper, opinion paper, experience paper
EC4	Research strategy	Case study, experiment, survey, grounded theory, user Study, field study, mixed study, exploratory study, literature review
EC5	Data collection method	Interview, meta-analysis, observing users, prototype development, questionnaire, systematic literature review, systematic mapping study, usability test, user experience evaluation, Wizard of Oz, measurement with sensors, simulation, existing database data analysis
EC6	Research standpoint	Accessible UI, adaptive UI, artificial intelligence, brain computer interface (BCI), human–computer interaction (HCI), human–machine interaction (HMI), intelligent interaction (II), intelligent UI
EC7	HCII development phase	Analysis, design, implementation, testing
EC8	Study environment	Laboratory setting, real-world setting
EC9	Recognition of	3D Gaze, activity, attention, behavior, body motion, etc.
EC10	Data source	Audio, audiovisual information, camera, ECG, EDA, EEG, EMG, EOG, eye gaze, etc.
EC11	Sensor type	Accelerometer, ambient sensors, biometric sensor, blood volume pulse sensor (BVP), camera, EEG sensor, eye tracker, etc.
EC12	AI methods used	Adaboost algorithm, ANN, back propagation neural network (BPNN), bag-of-features (BOF), Bayesian deep-learning network (BDLN), BN, bidirectional long short-term memory recurrent neural network (BLSTM), C4.5, Combinatorial fusion analysis (CFA), etc.

**Table 6:** Top ten journals and conferences regarding the number of published articles.

Journal	Nr. of Articles
*IEEE Access*	21
*Multimedia Tools and Applications*	9
*Expert Systems with Applications*	6
*IEEE Transactions on Affective Computing*	5
*Procedia Computer Science*	5
*IEEE Sensors Journal*	4
*IEEE Internet of Things Journal*	4
*International Journal of Human-Computer Studies*	3
*Sensors*	3
*Computers & Electrical Engineering*	3
Conference	**Nr. of articles**
International Conference on Affective Computing and Intelligent Interaction (ACII)	56
Humaine Association Conference on Affective Computing and Intelligent Interaction	24
Asian Conference on Affective Computing and Intelligent Interaction (ACII Asia)	15
International Conference on Intelligent Computing and Control Systems (ICICCS)	8
International Conference on Affective Computing and Intelligent Interaction Workshops and Demos (ACIIW)	7
IEEE International Conference on Cyber Technology in Automation, Control, and Intelligent Systems (CYBER)	5
International Conference on Intelligent Environments	5
International Conference on Intelligent Human-Machine Systems and Cybernetics	5
International conference on Intelligent User Interfaces	4
Lecture Notes in Computer Science (including subseries Lecture Notes in Artificial Intelligence and Lecture Notes in Bioinformatics)	4

**Table 7:** Top ten most cited studies by the number of all paper citations.

Title	Year	Journal/Conference	Nr. of Citations
Analysis of EEG Signals and Facial Expressions for Continuous Emotion Detection [129]	2016	*IEEE Transactions on Affective Computing*	219
Stress recognition using wearable sensors and mobile phones [101]	2013	Humaine Association Conference on Affective Computing and Intelligent Interaction	214
EmotionMeter: A Multimodal Framework for Recognizing Human Emotions [112]	2019	*IEEE Transactions on Cybernetics*	188
Sparse Autoencoder-Based Feature Transfer Learning for Speech Emotion Recognition [130]	2013	2013 Humaine Association Conference on Affective Computing and Intelligent Interaction	152
Deep-learning analysis of mobile physiological, environmental and location sensor data for emotion detection [131]	2019	*Information Fusion*	102
EEG-Based Mobile Robot Control Through an Adaptive Brain–Robot Interface [132]	2014	*IEEE Transactions on Systems, Man, and Cybernetics: Systems*	96
Gender-Driven Emotion Recognition Through Speech Signals For Ambient Intelligence Applications [27]	2013	*IEEE Transactions on Emerging Topics in Computing*	79
From Activity Recognition to Intention Recognition for Assisted Living Within Smart Homes [133]	2017	*IEEE Transactions on Human–Machine Systems*	78
Error weighted semi-coupled hidden markov model for audio-visual emotion recognition [134]	2012	*IEEE Transactions on Multimedia*	77
Detecting Naturalistic Expressions of Nonbasic Affect Using Physiological Signals [89]	2012	*IEEE Transactions on Affective Computing*	68

**Table 8:** Top ten most cited studies by the average number of paper citations per Year.

Title	Year	Journal/Conference	Average Citations per Year
EmotionMeter: A Multimodal Framework for Recognizing Human Emotions [112]	2019	*IEEE Transactions on Cybernetics*	62.67
Analysis of EEG Signals and Facial Expressions for Continuous Emotion Detection [129]	2016	*IEEE Transactions on Affective Computing*	36.5
Deep-learning analysis of mobile physiological, environmental and location sensor data for emotion detection [131]	2019	*Information Fusion*	34
Stress recognition using wearable sensors and mobile phones [101]	2013	Humaine Association Conference on Affective Computing and Intelligent Interaction	23.78
Identifying Stable Patterns over Time for Emotion Recognition from EEG [135]	2019	*IEEE Transactions on Affective Computing*	19
Sparse Autoencoder-Based Feature Transfer Learning for Speech Emotion Recognition [130]	2013	2013 Humaine Association Conference on Affective Computing and Intelligent Interaction	16.89
From Activity Recognition to Intention Recognition for Assisted Living Within Smart Homes [133]	2017	*IEEE Transactions on Human-Machine Systems*	15.6
FER-net: facial expression recognition using deep neural net [136]	2021	*Neural Computing and Applications*	15
MultiD-CNN: A multi-dimensional feature learning approach based on deep convolutional networks for gesture recognition in RGB-D image sequences [2]	2020	*Expert Systems with Applications*	13.5
EEG-Based Mobile Robot Control Through an Adaptive Brain–Robot Interface [132]	2014	*IEEE Transactions on Systems, Man, and Cybernetics: Systems*	12

**Table 9:** Top 10 countries regarding the contribution to the literature.

Country	Nr. of Articles	%
China	128	28
USA	57	13
India	47	10
United Kingdom	33	7
Germany	24	5
Japan	19	4
South Korea	18	4
Italy	13	3
Australia	13	3
Canada	12	3

## Data Availability

Not applicable.

## Supplementary Materials

The following are available online at https://www.mdpi.com/article/10.3390/s22010020/s1, Studies, included in the SMS.
