# Supplementary material for: Sensors and Artificial Intelligence Methods and Algorithms for Human–Computer Intelligent Interaction: A Systematic Mapping Study
Source: Sensors (Basel). 2021 Dec 21;22(1):20. doi: 10.3390/s22010020 (PMC8747169; doi:10.3390/s22010020)
Supplement: Supplementary file 1 [file sensors-22-00020-s001.zip › sensors-1488303-supplementary.pdf]

Studies, included in the SMS

[S1] C. Liang *et al.*, “Auth+Track: Enabling Authentication Free Interaction on Smartphone by Continuous User Tracking,” in *Proceedings of the 2021 CHI Conference on Human Factors in Computing Systems*, 2021, pp. 1–16.

[S2] Y. Huang and H. Zhao, “Chinese Pinyin Aided IME, Input What You Have Not Keystroked Yet,” in *Proceedings of the 2018 Conference on Empirical Methods in Natural Language Processing*, 2018, pp. 2923–2929.

[S3] N. Malsattar, T. Kihara, and E. Giaccardi, “Designing and Prototyping from the Perspective of AI in the Wild,” in *Proceedings of the 2019 on Designing Interactive Systems Conference*, 2019, pp. 1083–1088.

[S4] K. Katsuragawa, A. Kamal, and E. Lank, “Effect of Motion-Gesture Recognizer Error Pattern on User Workload and Behavior,” in *Proceedings of the 22nd International Conference on Intelligent User Interfaces*, 2017, pp. 439–449.

[S5] Y. Zhang, B. Coecke, and M. Chen, “MI3: Machine-initiated Intelligent Interaction for Interactive Classification and Data Reconstruction,” *ACM Trans. Interact. Intell. Syst.*, vol. 11, no. 3–4, pp. 1–34, Dec. 2021.

[S6] O. J. Romero *et al.*, “A Long-Term Evaluation of Adaptive Interface Design for Mobile Transit Information,” in *22nd International Conference on Human-Computer Interaction with Mobile Devices and Services*, 2020, pp. 1–11.

[S7] K. Todi, G. Bailly, L. Leiva, and A. Oulasvirta, “Adapting User Interfaces with Model-based Reinforcement Learning,” in *Proceedings of the 2021 CHI Conference on Human Factors in Computing Systems*, 2021, pp. 1–13.

[S8] M. Constantinides, J. Dowell, D. Johnson, and S. Malacria, “Exploring mobile news reading interactions for news app personalisation,” in *Proceedings of the 17th International Conference on Human-Computer Interaction with Mobile Devices and Services*, 2015, no. July, pp. 457–462.

[S9] J. Zhou *et al.*, “Measurable Decision Making with GSR and Pupillary Analysis for Intelligent User Interface,” *ACM Trans. Comput. Interact.*, vol. 21, no. 6, pp. 1–23, Jan. 2015.

[S10] B. Williford, M. Runyon, and T. Hammond, “Recognizing perspective accuracy,” in *Proceedings of the 25th International Conference on Intelligent User Interfaces*, 2020, pp. 231–242.

[S11] M. Sousa, J. Vieira, D. Medeiros, A. Arsenio, and J. Jorge, “SleeveAR: Augmented Reality for Rehabilitation using Realtime Feedback,” in *Proceedings of the 21st International Conference on Intelligent User Interfaces*, 2016, vol. 07-10-Marc, no. October 2017, pp. 175–185.

[S12] P. Tchankue, J. Wesson, and D. Vogts, “The impact of an adaptive user interface on reducing driver distraction,” in *Proceedings of the 3rd International Conference on Automotive User Interfaces and Interactive Vehicular Applications - AutomotiveUI ’11*, 2011, no. November, p. 87.

[S13] H. Alqahtani, C. Z. Liu, M. Kavakli-Thorne, and Y. Kang, “An Agent-Based Intelligent HCI Information System in Mixed Reality,” in *28TH INTERNATIONAL CONFERENCE ON INFORMATION SYSTEMS DEVELOPMENT*, 2019.

[S14] R. A. Antunes, L. B. Palma, H. Duarte-Ramos, and P. Gil, “Intelligent HCI Device for Assistive Technology,” vol. 553, no. April, L. M. Camarinha-Matos, R. Almeida, and J. Oliveira, Eds. Cham: Springer International Publishing, 2019, pp. 157–168.

[S15] S. M. Ashfaque and V. Bhosale, “Ostentatious Adoption of Hand Gestures for Intelligent HCI,” in *2015 International Conference on Computational Intelligence and Communication Networks (CICN)*, 2015, pp. 643–648.

[S16] J. Wu, J. Cheng, and W. Feng, “3D dynamic gesture recognition based on improved HMMs with entropy,” in *2014 IEEE International Conference on Information and Automation (ICIA)*, 2014, no. July, pp. 213–218.

[S17] M. Liu, Y. Fu Li, and H. Liu, “3D Gaze Estimation for Head-Mounted Devices based on Visual Saliency,” in *2020 IEEE/RSJ International Conference on Intelligent Robots and Systems (IROS)*, 2020, pp. 10611–10616.

[S18] B. Singh and J. Panda, “3D skeletal gesture recognition through discriminative coding: Human activity recognition using time warping invariant Riemannian trajectories,” in *2021 5th International Conference on Intelligent Computing and Control Systems (ICICCS)*, 2021, no. Iciccs, pp. 1379–1386.

[S19] J. Iskander, I. Hettiarachchi, S. Hanoun, M. Hossny, S. Nahavandi, and A. Bhatti, “A classifier approach to multi-screen switching based on low cost eye-trackers,” in *2018 Annual IEEE International Systems Conference (SysCon)*, 2018, pp. 1–6.

[S20] F. M. Garcia-Moreno, M. Bermudez-Edo, M. J. Rodriguez-Fortiz, and J. L. Garrido, “A CNN-LSTM Deep Learning Classifier for Motor Imagery EEG Detection Using a Low-invasive and Low-Cost BCI Headband,” in *2020 16th International Conference on Intelligent Environments (IE)*, 2020, pp. 84–91.

[S21] O. M. Al-Omair and S. Huang, “A Comparative Study of Algorithms and Methods for Facial Expression Recognition,” in *2019 IEEE International Systems Conference (SysCon)*, 2019, pp. 1–6.

[S22] F. Wang, J. Zhou, J. Lin, H. Wang, W. Wang, and J. Yang, “A comparative study on sign recognition using sEMG and inertial sensors,” in *2016 IEEE International Conference on Cyber Technology in Automation, Control, and Intelligent Systems (CYBER)*, 2016, pp. 290–295.

[S23] K. Wang, B. Xiao, J. Xia, and D. Li, “A Dynamic Hand Gesture Recognition Algorithm Using Codebook Model and Spatial Moments,” in *2015 7th International Conference on Intelligent Human-Machine Systems and Cybernetics*, 2015, vol. 1, pp. 130–133.

[S24] K. M. Pathak, S. Yadav, P. Jain, P. Tanwar, and B. Kumar, “A Facial Expression Recognition System To Predict Emotions,” in *2020 International Conference on Intelligent Engineering and Management (ICIEM)*, 2020, pp. 414–419.

[S25] Sudha V, Viswanath G, Balasubramanian A, Chiranjeevi P, Basant KP, and Pratibha M, “A fast and robust emotion recognition system for real-world mobile phone data,” in *2015 IEEE International Conference on Multimedia & Expo Workshops (ICMEW)*, 2015, pp. 1–6.

[S26] S. Ramkumar, K. S. Kumar, and G. Emayavaramban, “A feasibility study on eye movements using electrooculogram based HCI,” in *2017 International Conference on Intelligent Sustainable Systems (ICISS)*, 2017, pp. 380–383.

[S27] U. Afreen Farzana, S. Abirami, and M. Srivani, “A Framework For Captioning The Human Interactions,” in *2019 11th International Conference on Advanced Computing (ICoAC)*, 2019, pp. 13–17.

[S28] Jiyan Sun, Anhong Guo, and Shihan Xiao, “A general HCI system based on monocular camera for intelligent terminals,” in *2011 International Conference on Advanced Intelligence and Awareness Internet (AIAI 2011)*, 2011, vol. 2011, no. 588 CP, pp. 354–358.

[S29] N. Song, H. Yang, and P. Wu, “A Gesture-to-Emotional Speech Conversion by Combining Gesture Recognition and Facial Expression Recognition,” in *2018 First Asian Conference on Affective Computing and Intelligent Interaction (ACII Asia)*, 2018, pp. 1–6.

[S30] H. Cecotti, Y. K. Meena, and G. Prasad, “A multimodal virtual keyboard using eye-tracking and hand gesture detection,” in *2018 40th Annual International Conference of the IEEE Engineering in Medicine and Biology Society (EMBC)*, 2018, vol. 2018-July, pp. 3330–3333.

[S31] H. Cecotti, Y. K. Meena, B. Bhushan, A. Dutta, and G. Prasad, “A multiscript gaze-based assistive virtual keyboard,” in *2019 41st Annual International Conference of the IEEE Engineering in Medicine and Biology Society (EMBC)*, 2019, pp. 1306–1309.

[S32] N. Rossol, I. Cheng, and A. Basu, “A Multisensor Technique for Gesture Recognition Through Intelligent Skeletal Pose Analysis,” *IEEE Trans. Human-Machine Syst.*, vol. 46, no. 3, pp. 350–359, Jun. 2016.

[S33] J. Zeng, Y. Sun, and F. Wang, “A Natural Hand Gesture System for Intelligent Human-Computer Interaction and Medical Assistance,” in *2012 Third Global Congress on Intelligent Systems*, 2012, pp. 382–385.

[S34] B. Jiang, K. Jia, and Q. Wu, “A Novel Algorithm of Facial Expression Recognition Based on Discriminative Component Analysis,” in *2012 Eighth International Conference on Intelligent Information Hiding and Multimedia Signal Processing*, 2012, pp. 399–402.

[S35] M. Shi, L. Xu, and X. Chen, “A Novel Facial Expression Intelligent Recognition Method Using Improved Convolutional Neural Network,” *IEEE Access*, vol. 8, pp. 57606–57614, 2020.

[S36] Z. Xu, X. Qiu, and J. He, “A novel multimedia human-computer interaction (HCI) system based on Kinect and depth image understanding,” in *2016 International Conference on Inventive Computation Technologies (ICICT)*, 2016, pp. 1–6.

[S37] M. Gaudina, S. Schiappacasse, L. Lagomarsino, E. Bellanti, and G. Vercelli, “A Novel Multimedia Interactive System for Public Show and Presentations,” in *2014 Eighth International Conference on Complex, Intelligent and Software Intensive Systems*, 2014, pp. 543–546.

[S38] Y. K. Meena, H. Cecotti, K. Wong-Lin, and G. Prasad, “A novel multimodal gaze-controlled Hindi virtual keyboard for disabled users,” in *2016 IEEE International Conference on Systems, Man, and Cybernetics (SMC)*, 2016, pp. 003688–003693.

[S39] G. Shao, M. Che, B. Zhang, K. Cen, and W. Gao, “A Novel Simple 2D Model of Eye Gaze Estimation,” in *2010 Second International Conference on Intelligent Human-Machine Systems and Cybernetics*, 2010, vol. 1, pp. 300–304.

[S40] F. Manuri and G. Piumatti, “A Preliminary Study of a Hybrid User Interface for Augmented Reality Applications,” in *Proceedings of the 7th International Conference on Intelligent Technologies for Interactive Entertainment*, 2015, vol. 17, pp. 37–41.

[S41] S. Chen, H. Ma, and C. Han, “A Real-time Hand Postures Estimation Method,” in *2017 IEEE 7th Annual International Conference on CYBER Technology in Automation, Control, and Intelligent Systems (CYBER)*, 2017, pp. 1059–1064.

[S42] S. You, X. Li, and W. Chen, “A semantic mechanism for Internet-of-Things (IoT) to implement intelligent interactions,” in *2018 16th International Symposium on Modeling and Optimization in Mobile, Ad Hoc, and Wireless Networks (WiOpt)*, 2018, pp. 1–6.

[S43] M. Li, F. Wang, K. Jia, S. Zhao, and C. Li, “A Sign Language Interactive System based on Multi-feature Fusion,” in *2019 IEEE 9th Annual International Conference on CYBER Technology in Automation, Control, and Intelligent Systems (CYBER)*, 2019, pp. 237–241.

[S44] K. Ono and J.-H. Lee, “A smart phone based interaction in intelligent space using object recognition and facing direction of human,” in *Proceedings of the 2013 IEEE/SICE International Symposium on System Integration*, 2013, pp. 216–221.

[S45] C. Sirithunge, A. G. B. P. Jayasekara, and D. P. Chandima, “A Space-Filler Mechanism to Recognize Body Language of Hands,” in *2019 14th Conference on Industrial and Information Systems (ICIIS)*, 2019, pp. 338–343.

[S46] H. Elleuch, A. Wali, A. Samet, and A. M. Alimi, “A static hand gesture recognition system for real time mobile device monitoring,” in *2015 15th International Conference on Intelligent Systems Design and Applications (ISDA)*, 2015, vol. 2016-June, pp. 195–200.

[S47] M. Savargiv, M. R. Keyvanpour, and S. MehrMolaei, “A structural method based on fuzzy approach at producing emotional data,” in *2018 6th Iranian Joint Congress on Fuzzy and Intelligent Systems (CFIS)*, 2018, vol. 2018-Janua, pp. 180–184.

[S48] J. J. Bird, L. J. Manso, E. P. Ribeiro, A. Ekart, and D. R. Faria, “A Study on Mental State Classification using EEG-based Brain-Machine Interface,” in *2018 International Conference on Intelligent Systems (IS)*, 2018, pp. 795–800.

[S49] C. Liu, D. Yu, J. Zhang, and S. Xie, “A Utility Human Machine Interface Using Low Cost EEG Cap and Eye Tracker,” in *2021 9th International Winter Conference on Brain-Computer Interface (BCI)*, 2021, pp. 1–5.

[S50] S. Prasad, P. Kumar, and K. P. Sinha, “A wireless dynamic gesture user interface for HCI using hand data glove,” in *2014 Seventh International Conference on Contemporary Computing (IC3)*, 2014, pp. 62–67.

[S51] M. Abdelwahab and C. Busso, “Active Learning for Speech Emotion Recognition Using Deep Neural Network,” in *2019 8th International Conference on Affective Computing and Intelligent Interaction (ACII)*, 2019, pp. 1–7.

[S52] B. Marcheix, B. Gardiner, and S. Coleman, “Adaptive Gesture Recognition System for Robotic Control using Surface EMG Sensors,” in *2019 IEEE International Symposium on Signal Processing and Information Technology (ISSPIT)*, 2019, pp. 1–6.

[S53] D. Moazen, S. A. Sajjadi, and A. Nahapetian, "AirDraw: Leveraging smart watch motion sensors for mobile human computer interactions," in *2016 13th IEEE Annual Consumer Communications & Networking Conference (CCNC)*, 2016, pp. 442–446.

[S54] M. Garcia-Constantino, A. Konios, M. A. Mustafa, C. Nugent, and G. Morrison, "Ambient and Wearable Sensor Fusion for Abnormal Behaviour Detection in Activities of Daily Living," in *2020 IEEE International Conference on Pervasive Computing and Communications Workshops (PerCom Workshops)*, 2020, pp. 1–6.

[S55] V. Kartsch, M. Guermandi, S. Benatti, F. Montagna, and L. Benini, "An Energy-Efficient IoT node for HMI applications based on an ultra-low power Multicore Processor," in *2019 IEEE Sensors Applications Symposium (SAS)*, 2019, pp. 1–6.

[S56] R. Zhang *et al.*, "An EOG-Based Human–Machine Interface to Control a Smart Home Environment for Patients With Severe Spinal Cord Injuries," *IEEE Trans. Biomed. Eng.*, vol. 66, no. 1, pp. 89–100, Jan. 2019.

[S57] C. Chellaswamy, J. J. Durgadevi, and S. Srinivasan, "An intelligent hand gesture recognition system using fuzzy logic," in *IET Chennai Fourth International Conference on Sustainable Energy and Intelligent Systems (SEISCON 2013)*, 2013, vol. 2013, no. 8, pp. 326–332.

[S58] Z. Meng, K. Xie, and L. Yang, "An intelligent ordering control system based on hand gesture recognition," in *2020 International Conference on High Performance Big Data and Intelligent Systems, HPBD and IS 2020*, 2020, pp. 7–11.

[S59] H. Cao, "An Intelligent Speech Interaction Model for Mobile Teaching," in *2019 International Conference on Intelligent Transportation, Big Data & Smart City (ICITBS)*, 2019, pp. 170–173.

[S60] D. Yang, L. Ma, and F. Liao, "An Intelligent Voice Interaction System Based on Raspberry Pi," in *2019 11th International Conference on Intelligent Human-Machine Systems and Cybernetics (IHMSC)*, 2019, vol. 1, pp. 237–240.

[S61] F. Gonzalez, F. Gosselin, and W. Bachta, "Analysis of Hand Contact Areas and Interaction Capabilities During Manipulation and Exploration," *IEEE Trans. Haptics*, vol. 7, no. 4, pp. 415–429, Oct. 2014.

[S62] P. Wang, X. Lu, H. Sun, and W. Lv, "Application of speech recognition technology in IoT smart home," in *2019 IEEE 3rd Advanced Information Management, Communicates, Electronic and Automation Control Conference (IMCEC)*, 2019, no. Imcec, pp. 1264–1267.

[S63] H. Huang, X. Wang, M. Hu, and Y. Tao, "Applied to Mobile Multimedia Intelligent Speech System Interactive Topic Guiding Model," *IEEE Access*, vol. 7, pp. 182348–182356, 2019.

[S64] M. ElBadawy, A. S. Elons, H. A. Shedeed, and M. F. Tolba, "Arabic sign language recognition with 3D convolutional neural networks," in *2017 Eighth International Conference on Intelligent Computing and Information Systems (ICICIS)*, 2017, vol. 2018-Janua, no. Icicis, pp. 66–71.

[S65] M. Wang, Y. Maeda, K. Naruki, and Y. Takahashi, "Attention prediction system based on eye tracking and saliency map by fuzzy neural network," in *2014 Joint 7th International Conference on Soft Computing and Intelligent Systems (SCIS) and 15th International Symposium on Advanced Intelligent Systems (ISIS)*, 2014, pp. 339–342.

[S66] Q. Cheng, S. Zhang, S. Bo, D. Chen, and H. Zhang, "Augmented Reality Dynamic Image Recognition Technology Based on Deep Learning Algorithm," *IEEE Access*, vol. 8, pp. 137370–137384, 2020.

[S67] J. Deng, C. Leung, and Y. Li, "Beyond Big Data of Human Behaviors: Modeling Human Behaviors and Deep Emotions," in *2018 IEEE Conference on Multimedia Information Processing and Retrieval (MIPR)*, 2018, pp. 282–286.

[S68] M. Majewski and W. Kacalak, "Building Innovative Speech Interfaces Using Patterns and Antipatterns of Commands for Controlling Loader Cranes," in *2016 International Conference on Computational Science and Computational Intelligence (CSCI)*, 2016, pp. 525–530.

[S69] Y. Gu, W. Liu, and J. Song, "Can Activities of Human Daily Life be Recognized and Predicted?," in *2015 IEEE/WIC/ACM International Conference on Web Intelligence and Intelligent Agent Technology (WI-IAT)*, 2015, vol. 1, pp. 127–132.

[S70] K. Qian, T. Koike, K. Yoshiuchi, B. W. Schuller, and Y. Yamamoto, "Can Appliances Understand the Behavior of Elderly Via Machine Learning? A Feasibility Study," *IEEE Internet Things J.*, vol. 8, no. 10, pp. 8343–8355, May 2021.

[S71] S. Manal, S. OV, and S. KG, "Catch Gesture: An Ultimate Action Recognition Technology Using IoT Device," in *2020 4th International Conference on Intelligent Computing and Control Systems (ICICCS)*, 2020, no. Iciccs, pp. 363–367.

[S72] J. Li, F. Ren, S. Nishide, and X. Kang, "Character action recognition based on deep convolutional neural network and action sequence," in *2019 IEEE 6th International Conference on Cloud Computing and Intelligence Systems (CCIS)*, 2019, pp. 149–153.

[S73] N. Borges, L. Lindblom, B. Clarke, A. Gander, and R. Lowe, "Classifying Confusion: Autodetection of Communicative Misunderstandings using Facial Action Units," in *2019 8th International Conference on Affective Computing and Intelligent Interaction Workshops and Demos (ACIIW)*, 2019, pp. 401–406.

[S74] S. Khan, L. Chen, and H. Yan, "Co-Clustering to Reveal Salient Facial Features for Expression Recognition," *IEEE Trans. Affect. Comput.*, vol. 11, no. 2, pp. 348–360, Apr. 2020.

[S75] P. D. S. H. Gunawardane and N. T. Medagedara, "Comparison of hand gesture inputs of leap motion controller & data glove in to a soft finger," in *2017 IEEE International Symposium on Robotics and Intelligent Sensors (IRIS)*, 2017, vol. 2018-Janua, pp. 62–68.

[S76] S. Lokesh, G. Balakrishnan, S. Malathy, and K. Murugan, "Computer interaction to human through photorealistic facial model for inter-process communication," in *2010 2nd International Conference on Computing, Communication and Networking Technologies, ICCCNT 2010*, 2010.

[S77] Yuhai Lan, Jing Li, and Zhaojie Ju, "Data fusion-based real-time hand gesture recognition with Kinect V2," in *2016 9th International Conference on Human System Interactions (HSI)*, 2016, pp. 307–310.

[S78] L. Yang, D. Jiang, W. Han, and H. Sahli, "DCNN and DNN based multi-modal depression recognition," in *2017 Seventh International Conference on Affective Computing and Intelligent Interaction (ACII)*, 2017, vol. 2018-Janua, pp. 484–489.

[S79] A. D. Rasamoelina, F. Adjailia, and P. Sincak, "Deep Convolutional Neural Network for Robust Facial Emotion Recognition," in *2019 IEEE International Symposium on INnovations in Intelligent SysTems and Applications (INISTA)*, 2019, pp. 1–6.

[S80] N. Kalischek, P. Thiam, P. Bellmann, and F. Schwenker, "Deep Domain Adaptation for Facial Expression Analysis," in *2019 8th International Conference on Affective Computing and Intelligent Interaction Workshops and Demos (ACIIW)*, 2019, pp. 317–323.

[S81] K. G. Lore, N. Sweet, K. Kumar, N. Ahmed, and S. Sarkar, "Deep Value of Information Estimators for Collaborative Human-Machine Information Gathering," 2015.

[S82] T. Xu, Z. Feng, W. Zhang, X. Yang, and P. Yu, "Depth based Hand Gesture Recognition for Smart Teaching," in *2018 International Conference on Security, Pattern Analysis, and Cybernetics (SPAC)*, 2018, pp. 387–390.

[S83] Y. Ni and Y. Wang, "Design of a Smart Storytelling Toy Based on Voice Interaction," in *2019 2nd World Conference on Mechanical Engineering and Intelligent Manufacturing (WCMEIM)*, 2019, pp. 229–233.

[S84] K.-Y. Wang, Y.-L. Ho, Y.-D. Huang, and W.-C. Fang, "Design of Intelligent EEG System for Human Emotion Recognition with Convolutional Neural Network," in *2019 IEEE International Conference on Artificial Intelligence Circuits and Systems (AICAS)*, 2019, pp. 142–145.

[S85] S. Deb and S. Deb, "Designing an intelligent blink analyzer tool for effective human computer interaction through eye," in *2012 4th International Conference on Intelligent Human Computer Interaction (IHCI)*, 2012, pp. 1–5.

[S86] V. K. Sharma, "Designing of Face Recognition System," in *2019 International Conference on Intelligent Computing and Control Systems (ICCS)*, 2019, no. Iciccs, pp. 459–461.

[S87] G. Caridakis, S. Asteriadis, K. Karpouzis, and S. Kollias, "Detecting human behavior emotional cues in Natural Interaction," in *2011 17th International Conference on Digital Signal Processing (DSP)*, 2011, pp. 1–6.

[S88] O. AlZoubi, S. K. D'Mello, and R. A. Calvo, "Detecting Naturalistic Expressions of Nonbasic Affect Using Physiological Signals," *IEEE Trans. Affect. Comput.*, vol. 3, no. 3, pp. 298–310, Jul. 2012.

[S89] A. Murata, T. Doi, K. Kageyama, and W. Karwowski, "Development of an Eye-Gaze Input System With High Speed and Accuracy through Target Prediction Based on Homing Eye Movements," *IEEE Access*, vol. 9, pp. 22688–22697, 2021.

[S90] Y. Houyi, L. Yunxiang, and F. Linjun, "Development principles of gesture interaction in smart space," in *Proceedings 2014 IEEE International Conference on Security, Pattern Analysis, and Cybernetics (SPAC)*, 2014, pp. 146–151.

[S91] J. Wang, L. Zhang, C. Wang, X. Ma, Q. Gao, and B. Lin, "Device-Free Human Gesture Recognition With Generative Adversarial Networks," *IEEE Internet Things J.*, vol. 7, no. 8, pp. 7678–7688, Aug. 2020.

[S92] J. Li *et al.*, "Direct Speech-to-Image Translation," *IEEE J. Sel. Top. Signal Process.*, vol. 14, no. 3, pp. 517–529, Mar. 2020.

[S93] A. Ghasemi, S. Denman, S. Sridharan, and C. Fookes, "Discovery of facial motions using deep machine perception," in *2016 IEEE Winter Conference on Applications of Computer Vision (WACV)*, 2016, pp. 1–7.

[S94] H. Wang *et al.*, "Diverse Feature Blend Based on Filter-Bank Common Spatial Pattern and Brain Functional Connectivity for Multiple Motor Imagery Detection," *IEEE Access*, vol. 8, pp. 155590–155601, 2020.

[S95] A. Dapogny, K. Bailly, and S. Dubuisson, "Dynamic facial expression recognition by joint static and multi-time gap transition classification," in *2015 11th IEEE International Conference and Workshops on Automatic Face and Gesture Recognition (FG)*, 2015, pp. 1–6.

[S96] J. Fan, E. Bekele, Z. Warren, and N. Sarkar, "EEG analysis of facial affect recognition process of individuals with ASD performance prediction leveraging social context," in *2017 Seventh International Conference on Affective Computing and Intelligent Interaction Workshops and Demos (ACIIW)*, 2017, vol. 2018-Janua, pp. 38–43.

[S97] L. Yang and J. Liu, "EEG-Based Emotion Recognition Using Temporal Convolutional Network," in *2019 IEEE 8th Data Driven Control and Learning Systems Conference (DDCLS)*, 2019, pp. 437–442.

[S98] A. Baghdadi and Y. Aribi, "Effectiveness of dominance for Anxiety vs. Anger detection," in *2019 Fifth International Conference on Advances in Biomedical Engineering (ICABME)*, 2019, vol. 2019-Octob, pp. 1–4.

[S99] N. Anil and S. . Sreeletha, "EMG Based Gesture Recognition Using Machine Learning," in *2018 Second International Conference on Intelligent Computing and Control Systems (ICICCS)*, 2018, no. Iciccs, pp. 1560–1564.

[S100] Q. Zhang, C. Zheng, and C. Xiong, "EMG-based estimation of shoulder and elbow joint angles for intuitive myoelectric control," in *2015 IEEE International Conference on Cyber Technology in Automation, Control, and Intelligent Systems (CYBER)*, 2015, pp. 1912–1916.

[S101] A. Ghandeharioun, D. McDuff, M. Czerwinski, and K. Rowan, "EMMA: An Emotion-Aware Wellbeing Chatbot," in *2019 8th International Conference on Affective Computing and Intelligent Interaction (ACII)*, 2019, pp. 1–7.

[S102] M. S. Sinith, E. Aswathi, T. M. Deepa, C. P. Shameema, and S. Rajan, "Emotion recognition from audio signals using Support Vector Machine," in *2015 IEEE Recent Advances in Intelligent Computational Systems (RAICS)*, 2015, no. December, pp. 139–144.

[S103] C. A. Kothe, S. Makeig, and J. A. Onton, "Emotion Recognition from EEG during Self-Paced Emotional Imagery," in *2013 Humaine Association Conference on Affective Computing and Intelligent Interaction*, 2013, pp. 855–858.

[S104] J. Hakura, R. Domon, and H. Fujita, "Emotion recognition method using facial expressions and situation," in *2013 IEEE 12th International Conference on Intelligent Software Methodologies, Tools and Techniques (SoMeT)*, 2013, pp. 257–263.

[S105] F. Xu and Z. Wang, "Emotion Recognition Research Based on Integration of Facial Expression and Voice," in *2018 11th International Congress on Image and Signal Processing, BioMedical Engineering and Informatics (CISP-BMEI)*, 2018, pp. 1–6.

[S106] J. Zhao, S. Chen, S. Wang, and Q. Jin, "Emotion Recognition using Multimodal Features," in *2018 First Asian Conference on Affective Computing and Intelligent Interaction (ACII Asia)*, 2018, pp. 1–6.

[S107] L. Y. Mano, "Emotional condition in the Health Smart Homes environment: emotion recognition using ensemble of classifiers," in *2018 Innovations in Intelligent Systems and Applications (INISTA)*, 2018, pp. 1–8.

[S108] J. Fan and H. Touyama, “Emotional Face Retrieval with P300 Signals of Multiple Subjects,” in *2016 Joint 8th International Conference on Soft Computing and Intelligent Systems (SCIS) and 17th International Symposium on Advanced Intelligent Systems (ISIS)*, 2016, pp. 490–495.

[S109] L. Stanciu and F. Blidariu, “Emotional states recognition by interpreting facial features,” *2017 E-Health Bioeng. Conf. EHB 2017*, pp. 273–276, 2017.

[S110] R. Bock *et al.*, “Intraindividual and interindividual multimodal emotion analyses in Human-Machine-Interaction,” in *2012 IEEE International Multi-Disciplinary Conference on Cognitive Methods in Situation Awareness and Decision Support*, 2012, pp. 59–64.

[S111] T. Matlovic, P. Gaspar, R. Moro, J. Simko, and M. Bielikova, “Emotions detection using facial expressions recognition and EEG,” in *2016 11th International Workshop on Semantic and Social Media Adaptation and Personalization (SMAP)*, 2016, pp. 18–23.

[S112] M. Z. Iqbal, E. Mangina, and A. G. Campbell, “Exploring the Real-Time Touchless Hand Interaction and Intelligent Agents in Augmented Reality Learning Applications,” in *2021 7th International Conference of the Immersive Learning Research Network (iLRN)*, 2021, pp. 1–8.

[S113] F. Wang and L. Shen, “Expression Recognition Using Region Features And Facial Action Units,” in *2019 15th International Conference on Intelligent Environments (IE)*, 2019, pp. 9–15.

[S114] G. Yang *et al.*, “Face Mask Recognition System with YOLOV5 Based on Image Recognition,” in *2020 IEEE 6th International Conference on Computer and Communications (ICCC)*, 2020, vol. 1, no. January 2020, pp. 1398–1404.

[S115] R. S. Deshmukh, V. Jagtap, and S. Paygude, “Facial emotion recognition system through machine learning approach,” in *2017 International Conference on Intelligent Computing and Control Systems (ICICCS)*, 2017, pp. 272–277.

[S116] F. Khemakhem and H. Ltifi, “Facial Expression Recognition using Convolution Neural Network Enhancing with Pre-Processing Stages,” in *2019 IEEE/ACS 16th International Conference on Computer Systems and Applications (AICCSA)*, 2019, vol. 2019-Novem, pp. 1–7.

[S117] S. He, S. Wang, W. Lan, H. Fu, and Q. Ji, “Facial Expression Recognition Using Deep Boltzmann Machine from Thermal Infrared Images,” in *2013 Humaine Association Conference on Affective Computing and Intelligent Interaction*, 2013, pp. 239–244.

[S118] L.-C. Wang, M. Liu, X. Lu, and H.-J. Lin, “Facial Expression recognition via neurons partially activated discriminated ELM,” in *2018 13th World Congress on Intelligent Control and Automation (WCICA)*, 2018, vol. 2018-July, pp. 710–715.

[S119] L. Shao, “Facial Movements Recognition Using Multichannel EMG Signals,” in *2019 IEEE Fourth International Conference on Data Science in Cyberspace (DSC)*, 2019, pp. 561–566.

[S120] S. Mikhaylevskiy, V. Chernyavskiy, V. Pavlishen, I. Romanova, and R. Solovyev, “Fast Emotion Recognition Neural Network for IoT Devices,” in *2021 International Seminar on Electron Devices Design and Production (SED)*, 2021, pp. 1–6.

[S121] J. Y. Oh, J.-H. Park, and J.-M. Park, “FingerTouch: Touch Interaction Using a Fingernail-Mounted Sensor on a Head-Mounted Display for Augmented Reality,” *IEEE Access*, vol. 8, pp. 101192–101208, 2020.

[S122] P. S. Madanayake, W. A. D. K. Wickramasinghe, H. P. Liyanarachchi, H. M. D. M. Herath, A. Karunasena, and T. D. Perera, “Fitness Mate: Intelligent workout assistant using motion detection,” in *2016 IEEE International Conference on Information and Automation for Sustainability (ICIAfS)*, 2016, pp. 1–5.

[S123] D. Freeman, R. Vennelakanti, and S. Madhvanath, “Freehand pose-based Gestural Interaction: Studies and implications for interface design,” in *2012 4th International Conference on Intelligent Human Computer Interaction (IHCI)*, 2012, pp. 1–6.

[S124] J. Rafferty, C. D. Nugent, J. Liu, and L. Chen, “From Activity Recognition to Intention Recognition for Assisted Living Within Smart Homes,” *IEEE Trans. Human-Machine Syst.*, vol. 47, no. 3, pp. 368–379, Jun. 2017.

[S125] Z. Ni and Q. Li, “Fusion Learning Model for Mobile Face Safe Detection and Facial Gesture Analysis,” *IEEE Access*, vol. 8, pp. 61043–61050, 2020.

[S126] M. A. Uddin, M. S. Hossain, R. K. Pathan, and M. Biswas, “Gender Recognition from Human Voice using Multi-Layer Architecture,” in *2020 International Conference on INnovations in Intelligent SysTems and Applications (INISTA)*, 2020, pp. 1–7.

[S127] I. Bisio, A. Delfino, F. Lavagetto, M. Marchese, and A. Sciarrone, “Gender-Driven Emotion Recognition Through Speech Signals For Ambient Intelligence Applications,” *IEEE Trans. Emerg. Top. Comput.*, vol. 1, no. 2, pp. 244–257, Dec. 2013.

[S128] W. Xu, X. Gu, and G. Chen, “Generating Emotional Controllable Response Based on Multi-Task and Dual Attention Framework,” *IEEE Access*, vol. 7, pp. 93734–93741, 2019.

[S129] Y. Liu, M. Dong, S. Bi, Dakui Gao, Y. Jing, and L. Li, “Gesture recognition based on Kinect,” in *2016 IEEE International Conference on Cyber Technology in Automation, Control, and Intelligent Systems (CYBER)*, 2016, pp. 343–347.

[S130] Y. Zhao, Y. Liu, M. Dong, and S. Bi, “Multi-feature gesture recognition based on Kinect,” in *2016 IEEE International Conference on Cyber Technology in Automation, Control, and Intelligent Systems (CYBER)*, 2016, pp. 392–396.

[S131] H. Yokoyama, P. Schmalenberg, M. Farooq, and E. M. Dede, “Gesture Recognition by Machine Learning Combined with Geometric Calculation,” in *2020 IEEE 23rd International Conference on Intelligent Transportation Systems (ITSC)*, 2020, pp. 1–6.

[S132] B. Wang, Z. Chen, and J. Chen, “Gesture Recognition by Using Kinect Skeleton Tracking System,” in *2013 5th International Conference on Intelligent Human-Machine Systems and Cybernetics*, 2013, vol. 1, pp. 418–422.

[S133] S. Yang, S. Lee, and Y. Byun, “Gesture Recognition for Home Automation Using Transfer Learning,” in *2018 International Conference on Intelligent Informatics and Biomedical Sciences (ICIIBMS)*, 2018, pp. 136–138.

[S134] M.-C. Giuroiu and T. Marita, “Gesture recognition toolkit using a Kinect sensor,” in *2015 IEEE International Conference on Intelligent Computer Communication and Processing (ICCP)*, 2015, pp. 317–324.

[S135] S. Carrino, E. Mugellini, O. A. Khaled, and R. Ingold, “Gesture-based hybrid approach for HCI in ambient intelligent

environmments," in *2011 IEEE International Conference on Fuzzy Systems (FUZZ-IEEE 2011)*, 2011, pp. 86–93.

[S136] H. Kolkhorst, M. Tangermann, and W. Burgard, "Guess What I Attend: Interface-Free Object Selection Using Brain Signals," in *2018 IEEE/RSJ International Conference on Intelligent Robots and Systems (IROS)*, 2018, pp. 7111–7116.

[S137] R. Fukui, N. Hayakawa, M. Watanabe, H. Azumi, and M. Nakao, "Hand gesture interface for content browse using wearable wrist contour measuring device," in *2015 IEEE/RSJ International Conference on Intelligent Robots and Systems (IROS)*, 2015, vol. 2015-Decem, pp. 1222–1229.

[S138] A. K. Das, V. Laxmi, and S. Kumar, "Hand Gesture Recognition and Classification Technique in Real-Time," in *2019 International Conference on Vision Towards Emerging Trends in Communication and Networking (ViTECoN)*, 2019, pp. 1–5.

[S139] Y. Li *et al.*, "Hand Gesture Recognition and Real-time Game Control Based on A Wearable Band with 6-axis Sensors," in *2018 International Joint Conference on Neural Networks (IJCNN)*, 2018, vol. 2018-July, pp. 1–6.

[S140] M. Gudavalli and C. K. Mohan, "Hand Gesture Recognition Based on Cascading of Multiple Features," in *2018 International Conference on Intelligent Autonomous Systems (ICoIAS)*, 2018, pp. 28–34.

[S141] Y. Liu, Y. Yin, and S. Zhang, "Hand Gesture Recognition Based on HU Moments in Interaction of Virtual Reality," in *2012 4th International Conference on Intelligent Human-Machine Systems and Cybernetics*, 2012, vol. 1, pp. 145–148.

[S142] R. Zhang, Y. Ming, and J. Sun, "Hand gesture recognition with SURF-BOF based on Gray threshold segmentation," in *2016 IEEE 13th International Conference on Signal Processing (ICSP)*, 2016, vol. 0, pp. 118–122.

[S143] N. Otberdout, L. Ballihi, and D. Aboutajdine, "Hand pose estimation based on deep learning depth map for hand gesture recognition," in *2017 Intelligent Systems and Computer Vision (ISCV)*, 2017, pp. 1–8.

[S144] A. Suharsono and D. Liang, "Hand Stability Based Features for Touch Behavior Smartphone Authentication," in *2020 3rd IEEE International Conference on Knowledge Innovation and Invention (ICKII)*, 2020, pp. 167–170.

[S145] I.-C. Severin and D.-M. Dobrea, "Head Gesture Recognition based on 6DOF Inertial sensor using Artificial Neural Network," in *2020 International Symposium on Electronics and Telecommunications (ISETC)*, 2020, pp. 1–4.

[S146] R. T. Bankar and S. S. Salankar, "Head Gesture Recognition System Using Adaboost Algorithm with Obstacle Detection," in *2015 7th International Conference on Emerging Trends in Engineering & Technology (ICETET)*, 2015, vol. 2016-March, pp. 46–50.

[S147] S. Saha, R. Lahiri, A. Konar, B. Banerjee, and A. K. Nagar, "HMM-based gesture recognition system using kinect sensor for improvised human-computer interaction," in *2017 International Joint Conference on Neural Networks (IJCNN)*, 2017, vol. 2017-May, pp. 2776–2783.

[S148] A. Nadeem, A. Jalal, and K. Kim, "Human Actions Tracking and Recognition Based on Body Parts Detection via Artificial Neural Network," in *2020 3rd International Conference on Advancements in Computational Sciences (ICACS)*, 2020, pp. 1–6.

[S149] R. Vargic, M. Chlebo, and J. Kacur, "Human computer interaction using BCI based on sensorimotor rhythm," in *2015 IEEE 19th International Conference on Intelligent Engineering Systems (INES)*, 2015, pp. 91–95.

[S150] L. Dan, H. K. Ekenel, and O. Jun, "Human Gesture Analysis Using Multimodal Features," in *2012 IEEE International Conference on Multimedia and Expo Workshops*, 2012, pp. 471–476.

[S151] Y. Tao, Z. Yao, and B. Liang, "Human-Computer Interaction Using Fingertip Based on Kinect," in *2018 IEEE 4th Information Technology and Mechatronics Engineering Conference (ITOEC)*, 2018, no. Itoec, pp. 888–893.

[S152] E. K. Kalunga, S. Chevallier, O. Rabreau, and E. Monacelli, "Hybrid interface: Integrating BCI in multimodal human-machine interfaces," in *2014 IEEE/ASME International Conference on Advanced Intelligent Mechatronics*, 2014, pp. 530–535.

[S153] W.-L. Zheng, J.-Y. Zhu, and B.-L. Lu, "Identifying Stable Patterns over Time for Emotion Recognition from EEG," *IEEE Trans. Affect. Comput.*, vol. 10, no. 3, pp. 417–429, Jul. 2019.

[S154] S. Boucenna, P. Gaussier, P. Andry, and L. Hafemeister, "Imitation as a communication tool for online facial expression learning and recognition," in *2010 IEEE/RSJ International Conference on Intelligent Robots and Systems*, 2010, pp. 5323–5328.

[S155] S. Sumpeno, I. G. A. Dharmayasa, S. M. S. Nugroho, and D. Purwitasari, "Immersive Hand Gesture for Virtual Museum using Leap Motion Sensor Based on K-Nearest Neighbor," in *2019 International Conference on Computer Engineering, Network, and Intelligent Multimedia (CENIM)*, 2019, vol. 2019-Novem, pp. 1–6.

[S156] M. A. Qureshi, A. Aziz, M. A. Saeed, M. Hayat, and J. S. Rasool, "Implementation of an efficient algorithm for Human hand gesture identification," in *2011 Saudi International Electronics, Communications and Photonics Conference (SIECPC)*, 2011, pp. 1–5.

[S157] M. Admiraal, S. Wilson, and R. Vaidyanathan, "Improved formulation of the IMU and MARG orientation gradient descent algorithm for motion tracking in human-machine interfaces," in *2017 IEEE International Conference on Multisensor Fusion and Integration for Intelligent Systems (MFI)*, 2017, vol. 2017-Novem, no. Mfi, pp. 403–410.

[S158] S. Panev and A. Manolova, "Improved multi-camera 3D Eye Tracking for human-computer interface," in *2015 IEEE 8th International Conference on Intelligent Data Acquisition and Advanced Computing Systems: Technology and Applications (IDAACS)*, 2015, vol. 1, no. September, pp. 276–281.

[S159] L.-V. Nguyen-Dinh, D. Roggen, A. Calatroni, and G. Troster, "Improving online gesture recognition with template matching methods in accelerometer data," in *2012 12th International Conference on Intelligent Systems Design and Applications (ISDA)*, 2012, pp. 831–836.

[S160] S. R. Gunarathne, J. De Silva, E. M. C. P. Ekanayake, I. Samaradiwakara, P. S. Haddela, and P. A. Fernando, "Intellemo: A mobile instant messaging application with intelligent emotion identification," in *2013 IEEE 8th International Conference on Industrial and Information Systems*, 2013, pp. 627–632.

[S161] X. Lv, M. Fu, Y. Li, Y. Yang, F. Pu, and Y. Li, "Intelligent combination projection system," in *2014 International Conference on Multisensor Fusion and Information Integration for Intelligent Systems (MFI)*, 2014, pp. 1–6.

[S162] S. Chaudhary, S. Singh, V. K. Tukka, V. Parwal, and S. Sinha, "Intelligent Device Disambiguation for Smart Home

Control," in *2019 7th International Conference on Future Internet of Things and Cloud (FiCloud)*, 2019, pp. 303–307.

[S163] A. Bin Dris, A. Alsalman, A. Al-Wabil, and M. Aldosari, "Intelligent Gaze-Based Screening System for Autism," in *2019 2nd International Conference on Computer Applications & Information Security (ICCAIS)*, 2019, pp. 1–5.

[S164] J. Qi, G. Jiang, G. Li, Y. Sun, and B. Tao, "Intelligent Human-Computer Interaction Based on Surface EMG Gesture Recognition," *IEEE Access*, vol. 7, pp. 61378–61387, 2019.

[S165] R. A. Antunes, L. B. Palma, F. V. Coito, H. Duarteramos, and P. Gil, "Intelligent human-computer interface for improving pointing device usability and performance," in *2016 12th IEEE International Conference on Control and Automation (ICCA)*, 2016, vol. 2016-July, pp. 714–719.

[S166] S. Oniga, J. Vegh, and I. Orha, "Intelligent human-machine interface using hand gestures recognition," in *Proceedings of 2012 IEEE International Conference on Automation, Quality and Testing, Robotics*, 2012, pp. 559–563.

[S167] S. Noh, S. B. Lee, and J. Park, "Intelligent Interaction System Capable of Autonomous Mobility for At-Home Workouts," in *ICTC 2019 - 10th International Conference on ICT Convergence: ICT Convergence Leading the Autonomous Future*, 2019, pp. 653–657.

[S168] K.-J. Wang *et al.*, "Intelligent Wearable Virtual Reality (VR) Gaming Controller for People with Motor Disabilities," in *2018 IEEE International Conference on Artificial Intelligence and Virtual Reality (AIVR)*, 2018, pp. 161–164.

[S169] G. Dafoulas, A. Tsiakara, J. Samuels-Clarke, C. C. Maia, D. Neilson, and A. A. Ali, "Investigating patterns of emotion and expressions using smart learning spaces," in *2019 10th International Conference on Information and Communication Systems (ICICS)*, 2019, pp. 238–244.

[S170] E. V. Polyakov, M. S. Mazhanov, A. Y. Rolich, L. S. Voskov, M. V. Kachalova, and S. V. Polyakov, "Investigation and development of the intelligent voice assistant for the Internet of Things using machine learning," in *2018 Moscow Workshop on Electronic and Networking Technologies (MWENT)*, 2018, vol. 2018-March, pp. 1–5.

[S171] H. Liu, J. Tu, M. Liu, and R. Ding, "Learning Explicit Shape and Motion Evolution Maps for Skeleton-Based Human Action Recognition," in *2018 IEEE International Conference on Acoustics, Speech and Signal Processing (ICASSP)*, 2018, vol. 2018-April, no. 2, pp. 1333–1337.

[S172] A. Kannan, A. Ramesh, L. Srinivasan, and V. Vijayaraghavan, "Low-cost static gesture recognition system using MEMS accelerometers," in *2017 Global Internet of Things Summit (GIoTS)*, 2017, pp. 1–6.

[S173] M. Benmoussa and A. Mahmoudi, "Machine learning for hand gesture recognition using bag-of-words," in *2018 International Conference on Intelligent Systems and Computer Vision (ISCV)*, 2018, vol. 2018-May, pp. 1–7.

[S174] S. Feng, Z. Feng, and L. Cao, "Many-to-One Gesture-to-Command Flexible Mapping Approach for Smart Teaching Interface Interaction," *IEEE Access*, vol. 7, pp. 179517–179531, 2019.

[S175] S. Casaccia *et al.*, "Measurement of Users' Well-Being Through Domotic Sensors and Machine Learning Algorithms," *IEEE Sens. J.*, vol. 20, no. 14, pp. 8029–8038, Jul. 2020.

[S176] W.-J. Chang, L.-B. Chen, C.-H. Hsu, J.-H. Chen, T.-C. Yang, and C.-P. Lin, "MedGlasses: A Wearable Smart-Glasses-Based Drug Pill Recognition System Using Deep Learning for Visually Impaired Chronic Patients," *IEEE Access*, vol. 8, pp. 17013–17024, 2020.

[S177] R. G. Lupu, F. Ungureanu, and R. G. Bozomitu, "Mobile embedded system for human computer communication in assistive technology," in *2012 IEEE 8th International Conference on Intelligent Computer Communication and Processing*, 2012, pp. 209–212.

[S178] H. Xue and S. Qin, "Mobile motion gesture design for deaf people," in *Proceedings of 2011 17th International Conference on Automation and Computing, ICAC 2011*, 2011, no. September, pp. 46–50.

[S179] Y.-H. Chen, M.-J. Tsai, L.-C. Fu, C.-H. Chen, C.-L. Wu, and Y.-C. Zeng, "Monitoring Elder's Living Activity Using Ambient and Body Sensor Network in Smart Home," in *2015 IEEE International Conference on Systems, Man, and Cybernetics*, 2015, pp. 2962–2967.

[S180] R. Raynova, A. Aleksieva-Petrova, and M. Lazarova, "Multi-agent Multimodal Human Emotion Recognition Architecture," in *2020 28th National Conference with International Participation (TELECOM)*, 2020, pp. 37–40.

[S181] Q. Shi, Z. Zhang, and C. Lee, "Multi-Functional Human-Machine Interface (HMI) Using Flexible Wearable Triboelectric Nanogenerator for Diversified Interacting Applications," in *2019 19th International Conference on Micro and Nanotechnology for Power Generation and Energy Conversion Applications (PowerMEMS)*, 2019, no. c, pp. 1–4.

[S182] C. Saitis and K. Kalimeri, "Multimodal Classification of Stressful Environments in Visually Impaired Mobility Using EEG and Peripheral Biosignals," *IEEE Trans. Affect. Comput.*, vol. 12, no. 1, pp. 203–214, Jan. 2021.

[S183] T.-Y. Pan, C.-Y. Chang, W.-L. Tsai, and M.-C. Hu, "Multisensor-Based 3D Gesture Recognition for a Decision-Making Training System," *IEEE Sens. J.*, vol. 21, no. 1, pp. 706–716, Jan. 2021.

[S184] A. Hajlaoui, M. Chetouani, and S. Essid, "Multi-task Feature Learning for EEG-based Emotion Recognition Using Group Nonnegative Matrix Factorization," in *2018 26th European Signal Processing Conference (EUSIPCO)*, 2018, vol. 2018-Septe, pp. 91–95.

[S185] A. Saeed, A. Al-Hamadi, and R. Niese, "Neutral-independent geometric features for facial expression recognition," in *2012 12th International Conference on Intelligent Systems Design and Applications (ISDA)*, 2012, pp. 842–846.

[S186] G. Du, S. Long, and H. Yuan, "Non-Contact Emotion Recognition Combining Heart Rate and Facial Expression for Interactive Gaming Environments," *IEEE Access*, vol. 8, pp. 11896–11906, 2020.

[S187] A. Ghandeharioun *et al.*, "Objective assessment of depressive symptoms with machine learning and wearable sensors data," in *2017 Seventh International Conference on Affective Computing and Intelligent Interaction (ACII)*, 2017, vol. 2018-Janua, pp. 325–332.

[S188] D. Sonntag and T. Toyama, "On-Body IE: A Head-Mounted Multimodal Augmented Reality System for Learning and Recalling Faces," in *2013 9th International Conference on Intelligent Environments*, 2013, pp. 151–156.

[S189] M. Reale, T. Hung, and L. Yin, "Pointing with the eyes: Gaze estimation using a static/active camera system and 3D iris disk model," in *2010 IEEE International Conference on Multimedia and Expo*, 2010, pp. 280–285.

[S190] J. Hernandez-Aceituno and I. Sanchez-Berriel, "Programming natural interfaces through the combination of smart phone sensors," in *2018 XIII Technologies Applied to Electronics Teaching Conference (TAEE)*, 2018, pp. 1–7.

[S191] A. Panigrahi, J. P. Mohanty, A. K. Swain, and K. Mahapatra, "Real-Time Efficient Detection in Vision Based Static Hand Gesture Recognition," in *2018 IEEE International Symposium on Smart Electronic Systems (iSES) (Formerly iNiS)*, 2018, pp. 265–268.

[S192] S. Chaman, D. D'souza, B. D'mello, K. Bhavsar, and J. D'souza, "Real-Time Hand Gesture Communication System in Hindi for Speech and Hearing Impaired," in *2018 Second International Conference on Intelligent Computing and Control Systems (ICICCS)*, 2018, no. Iciccs, pp. 1954–1958.

[S193] W. Wu, M. Shi, T. Wu, D. Zhao, S. Zhang, and J. Li, "Real-time Hand Gesture Recognition Based on Deep Learning in Complex Environments," in *2019 Chinese Control And Decision Conference (CCDC)*, 2019, pp. 5950–5955.

[S194] H.-S. Park and K.-H. Jo, "Real-time hand gesture recognition for augmented screen using average background and camshift," in *The 19th Korea-Japan Joint Workshop on Frontiers of Computer Vision*, 2013, pp. 18–21.

[S195] Y. Zhu and B. Yuan, "Real-time hand gesture recognition with Kinect for playing racing video games," in *2014 International Joint Conference on Neural Networks (IJCNN)*, 2014, pp. 3240–3246.

[S196] N. Weiskirchen, R. Bock, and A. Wendemuth, "Recognition of emotional speech with convolutional neural networks by means of spectral estimates," in *2017 Seventh International Conference on Affective Computing and Intelligent Interaction Workshops and Demos (ACIIW)*, 2017, vol. 2018-Janua, pp. 50–55.

[S197] R. Bin Hossain, M. Sadat, and H. Mahmud, "Recognition of human affection in Smartphone perspective based on accelerometer and user's sitting position," in *2014 17th International Conference on Computer and Information Technology (ICCIT)*, 2014, pp. 87–91.

[S198] Z. Lian, Y. Li, J. Tao, J. Huang, and M. Niu, "Region Based Robust Facial Expression Analysis," in *2018 First Asian Conference on Affective Computing and Intelligent Interaction (ACII Asia)*, 2018, no. 61425017, pp. 1–5.

[S199] S. Ghosh, S. Goenka, N. Ganguly, B. Mitra, and P. De, "Representation Learning for Emotion Recognition from Smartphone Keyboard Interactions," in *2019 8th International Conference on Affective Computing and Intelligent Interaction (ACII)*, 2019, pp. 704–710.

[S200] J. Yuan, Z. Feng, D. Dong, X. Meng, J. Meng, and D. Kong, "Research on Multimodal Perceptual Navigational Virtual and Real Fusion Intelligent Experiment Equipment and Algorithm," *IEEE Access*, vol. 8, pp. 43375–43390, 2020.

[S201] G. Liqing, L. Wenwen, S. Yong, W. Yanyan, and L. Guoming, "Research on Portable Sign Language Translation System Based on Embedded System," in *2018 3rd International Conference on Smart City and Systems Engineering (ICSCSE)*, 2018, pp. 636–639.

[S202] J.-H. Sun, T.-T. Ji, S.-B. Zhang, J.-K. Yang, and G.-R. Ji, "Research on the Hand Gesture Recognition Based on Deep Learning," in *2018 12th International Symposium on Antennas, Propagation and EM Theory (ISAPE)*, 2018, pp. 1–4.

[S203] H. Liang, Y. Zhao, J. Wei, D. Quan, R. Cheng, and Y. Wei, "Robust Hand Detection and Tracking Based on Monocular Vision," in *2014 Sixth International Conference on Intelligent Human-Machine Systems and Cybernetics*, 2014, vol. 2, pp. 134–137.

[S204] H. Liu and X. Liu, "Robust hand tracking based on online learning and multi-cue flocks of features," in *2013 IEEE International Conference on Image Processing*, 2013, pp. 3725–3729.

[S205] P. Ithaya Rani and K. Muneeswaran, "Robust real time face detection automatically from video sequence based on Haar features," in *2014 International Conference on Communication and Network Technologies*, 2014, vol. 2015-March, pp. 276–280.

[S206] H. S. Chudgar, S. Mukherjee, and K. Sharma, "S control: Accelerometer-based gesture recognition for media control," in *2014 International Conference on Advances in Electronics Computers and Communications*, 2014, pp. 1–6.

[S207] M. He and J. Cheng, "Self-adaptive coding-based touch detection for interactive projector system," in *2014 4th IEEE International Conference on Information Science and Technology*, 2014, pp. 656–659.

[S208] E. Sopov and I. Ivanov, "Self-Configuring Ensemble of Neural Network Classifiers for Emotion Recognition in the Intelligent Human-Machine Interaction," in *2015 IEEE Symposium Series on Computational Intelligence*, 2015, pp. 1808–1815.

[S209] V. Gatteschi, F. Lamberti, P. Montuschi, and A. Sanna, "Semantics-Based Intelligent Human-Computer Interaction," *IEEE Intell. Syst.*, vol. 31, no. 4, pp. 11–21, Jul. 2016.

[S210] P. Pirzada, N. White, and A. Wilde, "Sensors in Smart Homes for Independent Living of the Elderly," in *2018 5th International Multi-Topic ICT Conference (IMTIC)*, 2018, pp. 1–8.

[S211] L. T. D'Angelo, J. Neuhaeuser, Yan Zhao, and T. C. Lueth, "SIMPLE-Use—Sensor Set for Wearable Movement and Interaction Research," *IEEE Sens. J.*, vol. 14, no. 4, pp. 1207–1215, Apr. 2014.

[S212] K. Haratiannejadi and R. R. Selmic, "Smart Glove and Hand Gesture-Based Control Interface for Multi-Rotor Aerial Vehicles in a Multi-Subject Environment," *IEEE Access*, vol. 8, pp. 227667–227677, 2020.

[S213] J. Berrezueta-Guzman, I. Pau, M.-L. Martin-Ruiz, and N. Maximo-Bocanegra, "Smart-Home Environment to Support Homework Activities for Children," *IEEE Access*, vol. 8, pp. 160251–160267, 2020.

[S214] A. Bastes, S. Alhat, and M. S. Panse, "Speech Assistive Communication System Using EOG," in *2018 Second International Conference on Intelligent Computing and Control Systems (ICICCS)*, 2018, no. Iciccs, pp. 504–510.

[S215] D. Muttaqin and S. Suyanto, "Speech Emotion Detection Using Mel-Frequency Cepstral Coefficient and Hidden Markov Model," in *2020 3rd International Seminar on Research of Information Technology and Intelligent Systems (ISRITI)*, 2020, pp. 463–466.

[S216] A. Sano and R. W. Picard, "Stress recognition using wearable sensors and mobile phones," in *Proceedings - 2013 Humaine Association Conference on Affective Computing and Intelligent Interaction, ACII 2013*, 2013, pp. 671–676.

[S217] Y. Wang, Y. Li, Y. Song, and X. Rong, "The Application of a Hybrid Transfer Algorithm Based on a Convolutional Neural Network Model and an Improved Convolution Restricted Boltzmann Machine Model in Facial Expression

Recognition," *IEEE Access*, vol. 7, pp. 184599–184610, 2019.

[S218] L. Alam and M. M. Hoque, "The design of expressive intelligent agent for human-computer interaction," in *2015 International Conference on Electrical Engineering and Information Communication Technology (ICEEICT)*, 2015, no. May, pp. 1–6.

[S219] R. B. Widodo *et al.*, "The IMU and Bend Sensor as a Pointing Device and Click Method," in *2019 International Seminar on Intelligent Technology and Its Applications (ISITIA)*, 2019, pp. 294–297.

[S220] Y. Chen, H. Zhao, and J. Chen, "The Integration Method of Multimodal Human-Computer Interaction Framework," in *2016 8th International Conference on Intelligent Human-Machine Systems and Cybernetics (IHMSC)*, 2016, vol. 2, pp. 545–550.

[S221] J. Yu, S. An, X. Ruan, N. Yu, and D. Gong, "The Realization and Optimization of Static Gesture Recognition System Based on Representation Learning," in *2019 Chinese Control And Decision Conference (CCDC)*, 2019, pp. 3250–3256.

[S222] L. Chang, "The research of face detection method based on adaboost algorithm and skin color segmentation," in *IST 2017 - IEEE International Conference on Imaging Systems and Techniques, Proceedings*, 2017, vol. 2018-Janua, no. 1, pp. 1–5.

[S223] D. Wang, J. Qu, W. Wang, G. Zhang, and S. Zhu, "The verification system for interface intelligent perception of human-computer interaction," in *2020 International Conference on Intelligent Computing and Human-Computer Interaction (ICHCI)*, 2020, pp. 43–48.

[S224] I.-C. Severin, "Time Series Feature Extraction For Head Gesture Recognition: Considerations Toward HCI Applications," in *2020 24th International Conference on System Theory, Control and Computing (ICSTCC)*, 2020, pp. 232–237.

[S225] Dongseok Ryu, Dugan Um, P. Tanofsky, Do Hyong Koh, Young Sam Ryu, and Sungchul Kang, "T-less : A novel touchless human-machine interface based on infrared proximity sensing," in *2010 IEEE/RSJ International Conference on Intelligent Robots and Systems*, 2010, pp. 5220–5225.

[S226] J. Dai and C.-K. R. Chung, "Touchscreen Everywhere: On Transferring a Normal Planar Surface to a Touch-Sensitive Display," *IEEE Trans. Cybern.*, vol. 44, no. 8, pp. 1383–1396, Aug. 2014.

[S227] R. A. H. Acuna, C. Fidas, V. Argyriou, and S. A. Velastin, "Toward a Two-Handed Gesture-Based Visual 3D Interactive Object-Oriented Environment for Software Development," in *2012 Eighth International Conference on Intelligent Environments*, 2012, pp. 359–362.

[S228] Y. K. Meena, H. Cecotti, K. Wong-Lin, A. Dutta, and G. Prasad, "Toward Optimization of Gaze-Controlled Human–Computer Interaction: Application to Hindi Virtual Keyboard for Stroke Patients," *IEEE Trans. Neural Syst. Rehabil. Eng.*, vol. 26, no. 4, pp. 911–922, Apr. 2018.

[S229] G. Leslie, A. Ojeda, and S. Makeig, "Towards an Affective Brain-Computer Interface Monitoring Musical Engagement," in *2013 Humaine Association Conference on Affective Computing and Intelligent Interaction*, 2013, pp. 871–875.

[S230] A. Ghandeharioun, D. McDuff, M. Czerwinski, and K. Rowan, "Towards Understanding Emotional Intelligence for Behavior Change Chatbots," in *2019 8th International Conference on Affective Computing and Intelligent Interaction (ACII)*, 2019, pp. 8–14.

[S231] S. Park, W. K. Chung, and K. Kim, "Training-Free Bayesian Self-Adaptive Classification for sEMG Pattern Recognition Including Motion Transition," *IEEE Trans. Biomed. Eng.*, vol. 67, no. 6, pp. 1775–1786, Jun. 2020.

[S232] Z. Chen, Y. Wang, and H. Liu, "Unobtrusive Sensor-Based Occupancy Facing Direction Detection and Tracking Using Advanced Machine Learning Algorithms," *IEEE Sens. J.*, vol. 18, no. 15, pp. 6360–6368, Aug. 2018.

[S233] R. Murugappan, J. J. Bosco, K. Eswaran, P. Vijay, and V. Vijayaraghavan, "User Independent Human Stress Detection," in *2020 IEEE 10th International Conference on Intelligent Systems (IS)*, 2020, pp. 490–497.

[S234] J. Tian, Z. Tu, Z. Wang, X. Xu, and M. Liu, "User Intention Recognition and Requirement Elicitation Method for Conversational AI Services," in *2020 IEEE International Conference on Web Services (ICWS)*, 2020, pp. 273–280.

[S235] X. Su, Y. Zhang, Q. Zhao, and L. Gao, "Virtual keyboard: A human-computer interaction device based on laser and image processing," in *2015 IEEE International Conference on Cyber Technology in Automation, Control, and Intelligent Systems (CYBER)*, 2015, no. 61471110, pp. 321–325.

[S236] P. Szczesny, J. Nikodem, and K. Kluwak, "Visual communication in expanding of human-computer interactions," in *2016 7th IEEE International Conference on Cognitive Infocommunications (CogInfoCom)*, 2016, no. CogInfoCom, pp. 000391–000396.

[S237] V. Joseph, A. Talpade, N. Suvarna, and Z. Mendonca, "Visual Gesture Recognition for Text Writing in Air," in *2018 Second International Conference on Intelligent Computing and Control Systems (ICICCS)*, 2018, no. Iciccs, pp. 23–26.

[S238] Z. Guo, F. Xiao, B. Sheng, H. Fei, and S. Yu, "WiReader: Adaptive Air Handwriting Recognition Based on Commercial WiFi Signal," *IEEE Internet Things J.*, vol. 7, no. 10, pp. 10483–10494, Oct. 2020.

[S239] E. Machado *et al.*, "A Conceptual framework for Adaptive User Interfaces for older adults," in *2018 IEEE International Conference on Pervasive Computing and Communications Workshops (PerCom Workshops)*, 2018, pp. 782–787.

[S240] N. Rathnayake, D. Meedeniya, I. Perera, and A. Welivita, "A Framework for Adaptive User Interface Generation based on User Behavioural Patterns," in *2019 Moratuwa Engineering Research Conference (MERCon)*, 2019, pp. 698–703.

[S241] G. Taylor, R. Frederiksen, J. Crossman, M. Quist, and P. Theisen, "A multi-modal intelligent user interface for supervisory control of unmanned platforms," in *2012 International Conference on Collaboration Technologies and Systems (CTS)*, 2012, pp. 117–124.

[S242] Ma Huijun and Xie Chao, "A system framework of adaptive user interface development for cartographic visualization system," in *2010 The 2nd Conference on Environmental Science and Information Application Technology*, 2010, pp. 333–336.

[S243] C. Inibhunu *et al.*, "Adapting level of detail in user interfaces for Cybersecurity operations," in *2016 Resilience Week*

(RWS), 2016, pp. 13–16.
[S244] I. B. Wijayasinghe, M. N. Saadatzi, S. Peetha, D. O. Popa, and S. Cremer, "Adaptive Interface for Robot Teleoperation using a Genetic Algorithm," in *2018 IEEE 14th International Conference on Automation Science and Engineering (CASE)*, 2018, vol. 2018-Augus, pp. 50–56.
[S245] I. A. Awada, I. Mocanu, D.-I. Nastac, D. Benta, and S. Radu, "Adaptive User Interface for Healthcare Application for People with Dementia," in *2018 17th RoEduNet Conference: Networking in Education and Research (RoEduNet)*, 2018, pp. 1–5.
[S246] H. Lee, Y. S. Choi, and Y.-J. Kim, "An adaptive user interface based on spatiotemporal structure learning," in *IEEE Communications Magazine*, 2011, vol. 49, no. 6, pp. 118–124.
[S247] K. Dalvand and M. Kazemifard, "An Adaptive User-Interface Based on User's Emotion," in *2012 2nd International eConference on Computer and Knowledge Engineering (ICCKE)*, 2012, pp. 161–166.
[S248] H.-Z. Tan, W. Zhao, and H.-H. Shen, "Adaptive user interface optimization for multi-screen based on machine learning," in *2018 IEEE 22nd International Conference on Computer Supported Cooperative Work in Design ((CSCWD))*, 2018, pp. 743–748.
[S249] R. Jain, J. Bose, and T. Arif, "Context based adaptation of application icons in mobile computing devices," in *2013 Third World Congress on Information and Communication Technologies (WICT 2013)*, 2013, pp. 31–36.
[S250] R. Jain, J. Bose, and T. Arif, "Contextual adaptive user interface for Android devices," in *2013 Annual IEEE India Conference (INDICON)*, 2013, pp. 1–5.
[S251] D. Reguera-Bakhache, I. Garitano, R. Uribeetxeberria, C. Cernuda, and U. Zurutuza, "Data-Driven Industrial Human-Machine Interface Temporal Adaptation for Process Optimization," in *2020 25th IEEE International Conference on Emerging Technologies and Factory Automation (ETFA)*, 2020, vol. 2020-Septe, pp. 518–525.
[S252] L. Zhang, "Developments in metaphor inference and context-aware affect sensing," in *2011 IEEE Conference on Computational Intelligence and Games (CIG'11)*, 2011, pp. 150–157.
[S253] C. Sun, Y. Wang, J. Zheng, and D. F. Hsu, "Feature fusion for mobile usage prediction using rank-score characteristics," in *2013 IEEE 12th International Conference on Cognitive Informatics and Cognitive Computing*, 2013, no. 2, pp. 212–217.
[S254] A. M. Syskov, V. I. Borisov, and V. S. Kublanov, "Intelligent multimodal user interface for telemedicine application," in *2017 25th Telecommunication Forum (TELFOR)*, 2017, vol. 2017-Janua, pp. 1–4.
[S255] K. Nazemi, C. Stab, and D. W. Fellner, "Interaction analysis: An algorithm for interaction prediction and activity recognition in adaptive systems," in *2010 IEEE International Conference on Intelligent Computing and Intelligent Systems*, 2010, pp. 607–612.
[S256] S. V. Kolekar, S. G. Sanjeevi, and D. S. Bormane, "Learning style recognition using Artificial Neural Network for adaptive user interface in e-learning," in *2010 IEEE International Conference on Computational Intelligence and Computing Research*, 2010, pp. 1–5.
[S257] L. Frieoteaux and I. Thouvenin, "OSE: An adaptive user interface for fluvial navigation training," in *2013 IEEE Virtual Reality (VR)*, 2013, pp. 111–112.
[S258] M. Nivethika, I. Vithiya, S. Anntharshika, and S. Deegalla, "Personalized and adaptive user interface framework for mobile application," in *2013 International Conference on Advances in Computing, Communications and Informatics (ICACCI)*, 2013, pp. 1913–1918.
[S259] A. Mejia-Figueroa and R. Juarez-Ramirez, "Towards a User Model for the Design of Adaptive Interfaces for Autistic Users," in *2014 IEEE 38th International Computer Software and Applications Conference Workshops*, 2014, pp. 264–269.
[S260] R. Hervás and J. Bravo, "Towards the ubiquitous visualization: Adaptive user-interfaces based on the Semantic Web," *Interact. Comput.*, vol. 23, no. 1, pp. 40–56, Jan. 2011.
[S261] E. Stefanidi, M. Foukarakis, D. Arampatzis, M. Korozi, A. Leonidis, and M. Antona, "ParlAmI: A Multimodal Approach for Programming Intelligent Environments," *Technologies*, vol. 7, no. 1, p. 11, Jan. 2019.
[S262] L. Gomes, C. Ramos, A. Jozi, B. Serra, L. Paiva, and Z. Vale, "IoH: A Platform for the Intelligence of Home with a Context Awareness and Ambient Intelligence Approach," *Futur. Internet*, vol. 11, no. 3, p. 58, Mar. 2019.
[S263] H. Ro, J.-H. Byun, Y. J. Park, N. K. Lee, and T.-D. Han, "AR Pointer: Advanced Ray-Casting Interface Using Laser Pointer Metaphor for Object Manipulation in 3D Augmented Reality Environment," *Appl. Sci.*, vol. 9, no. 15, p. 3078, Jul. 2019.
[S264] R.-C. Mihailescu, G. Kyriakou, and A. Papangelis, "Natural Language Understanding for Multi-Level Distributed Intelligent Virtual Sensors," *IoT*, vol. 1, no. 2, pp. 494–505, Dec. 2020.
[S265] K. Xia, X. Xie, H. Fan, and H. Liu, "An Intelligent Hybrid–Integrated System Using Speech Recognition and a 3D Display for Early Childhood Education," *Electronics*, vol. 10, no. 15, p. 1862, Aug. 2021.
[S266] S. Nayak, B. Nagesh, A. Routray, and M. Sarma, "A Human–Computer Interaction framework for emotion recognition through time-series thermal video sequences," *Comput. Electr. Eng.*, vol. 93, no. July 2020, p. 107280, Jul. 2021.
[S267] G. Xiao, Y. Ma, C. Liu, and D. Jiang, "A machine emotion transfer model for intelligent human-machine interaction based on group division," *Mech. Syst. Signal Process.*, vol. 142, p. 106736, Aug. 2020.
[S268] Q. Jin, C. Mu, L. Tian, and F. Ran, "A Region Generation based Model for Occluded Face Detection," *Procedia Comput. Sci.*, vol. 174, no. 2019, pp. 454–462, 2020.
[S269] K. R. Wang, B. J. Xiao, J. Y. Xia, D. Li, and W. L. Luo, "A real-time vision-based hand gesture interaction system for virtual EAST," *Fusion Eng. Des.*, vol. 112, pp. 829–834, Nov. 2016.
[S270] J. Hoey, T. Schröder, and A. Alhothali, "Affect control processes: Intelligent affective interaction using a partially observable Markov decision process," *Artif. Intell.*, vol. 230, pp. 134–172, Jan. 2016.
[S271] J. Kumar and J. Kumar, "Affective Modelling of Users in HCI Using EEG," *Procedia Comput. Sci.*, vol. 84, pp. 107–114, 2016.

[S272] M. Caon, L. Angelini, O. A. Khaled, D. Lalanne, Y. Yue, and E. Mugellini, "Affective Interaction in Smart Environments," *Procedia Comput. Sci.*, vol. 32, pp. 1016–1021, Mar. 2014.

[S273] X. Wang, A. Lu, J. Liu, Z. Kang, and C. Pan, "Intelligent interaction model for battleship control based on the fusion of target intention and operator emotion," *Comput. Electr. Eng.*, vol. 92, no. December 2020, p. 107196, Jun. 2021.

[S274] A. Elboushaki, R. Hannane, K. Afdel, and L. Koutti, "MultiD-CNN: A multi-dimensional feature learning approach based on deep convolutional networks for gesture recognition in RGB-D image sequences," *Expert Syst. Appl.*, vol. 139, p. 112829, Jan. 2020.

[S275] E. A. Suma, D. M. Krum, B. Lange, S. Koenig, A. Rizzo, and M. Bolas, "Adapting user interfaces for gestural interaction with the flexible action and articulated skeleton toolkit," *Comput. Graph.*, vol. 37, no. 3, pp. 193–201, May 2013.

[S276] C.-Y. Lu, S.-H. Lin, J.-C. Liu, S. Cruz-Lara, and J.-S. Hong, "Automatic event-level textual emotion sensing using mutual action histogram between entities," *Expert Syst. Appl.*, vol. 37, no. 2, pp. 1643–1653, Mar. 2010.

[S277] I.-J. Song and S.-B. Cho, "Bayesian and behavior networks for context-adaptive user interface in a ubiquitous home environment," *Expert Syst. Appl.*, vol. 40, no. 5, pp. 1827–1838, Apr. 2013.

[S278] S. Medjden, N. Ahmed, and M. Lataifeh, "Design and Analysis of an Automatic UI Adaptation Framework from Multimodal Emotion Recognition using an RGB-D Sensor," *Procedia Comput. Sci.*, vol. 170, pp. 82–89, 2020.

[S279] Ç. Çığ and T. Metin Sezgin, "Gaze-based prediction of pen-based virtual interaction tasks," *Int. J. Hum. Comput. Stud.*, vol. 73, pp. 91–106, Jan. 2015.

[S280] Ç. Çığ Karaman and T. M. Sezgin, "Gaze-based predictive user interfaces: Visualizing user intentions in the presence of uncertainty," *Int. J. Hum. Comput. Stud.*, vol. 111, no. December 2017, pp. 78–91, Mar. 2018.

[S281] E. Miller, Z. Li, H. Mentis, A. Park, T. Zhu, and N. Banerjee, "RadSense: Enabling one hand and no hands interaction for sterile manipulation of medical images using Doppler radar," *Smart Heal.*, vol. 15, no. November 2019, p. 100089, Mar. 2020.

[S282] C. Conati, O. Barral, V. Putnam, and L. Rieger, "Toward personalized XAI: A case study in intelligent tutoring systems," *Artif. Intell.*, vol. 298, p. 103503, Sep. 2021.

[S283] J. Li, J. Wang, and Z. Ju, "A Novel Hand Gesture Recognition Based on High-Level Features," *Int. J. Humanoid Robot.*, vol. 15, no. 02, p. 1750022, Apr. 2018.

[S284] R. Francese, M. Risi, and G. Tortora, "A user-centered approach for detecting emotions with low-cost sensors," *Multimed. Tools Appl.*, vol. 79, no. 47–48, pp. 35885–35907, Dec. 2020.

[S285] M. R. Mowla, R. I. Cano, K. J. Dhuyvetter, and D. E. Thompson, "Affective brain-computer interfaces: Choosing a meaningful performance measuring metric," *Comput. Biol. Med.*, vol. 126, no. August, p. 104001, Nov. 2020.

[S286] J. Chen, Y. Lv, R. Xu, and C. Xu, "Automatic social signal analysis: Facial expression recognition using difference convolution neural network," *J. Parallel Distrib. Comput.*, vol. 131, pp. 97–102, Sep. 2019.

[S287] Y. Huang, J. Yang, S. Liu, and J. Pan, "Combining facial expressions and electroencephalography to enhance emotion recognition," *Futur. Internet*, vol. 11, no. 5, pp. 1–17, 2019.

[S288] E. Kanjo, E. M. G. Younis, and C. S. Ang, "Deep learning analysis of mobile physiological, environmental and location sensor data for emotion detection," *Inf. Fusion*, vol. 49, no. September 2018, pp. 46–56, Sep. 2019.

[S289] J. Chen, R. Xu, and L. Liu, "Deep peak-neutral difference feature for facial expression recognition," *Multimed. Tools Appl.*, vol. 77, no. 22, pp. 29871–29887, Nov. 2018.

[S290] Y. Liu *et al.*, "Dynamic multi-channel metric network for joint pose-aware and identity-invariant facial expression recognition," *Inf. Sci. (Ny).*, vol. 578, pp. 195–213, Nov. 2021.

[S291] Akmm. Rahman, A. I. Anam, and M. Yeasin, "E m o A s s i s t: emotion enabled assistive tool to enhance dyadic conversation for the blind," *Multimed. Tools Appl.*, vol. 76, no. 6, pp. 7699–7730, Mar. 2017.

[S292] M. R. Islam *et al.*, "EEG Channel Correlation Based Model for Emotion Recognition," *Comput. Biol. Med.*, vol. 136, no. August, p. 104757, Sep. 2021.

[S293] N. Jain, S. Kumar, and A. Kumar, "Effective approach for facial expression recognition using hybrid square-based diagonal pattern geometric model," *Multimed. Tools Appl.*, vol. 78, no. 20, pp. 29555–29571, Oct. 2019.

[S294] S. Ghosh, K. Hiware, N. Ganguly, B. Mitra, and P. De, "Emotion detection from touch interactions during text entry on smartphones," *Int. J. Hum. Comput. Stud.*, vol. 130, no. April, pp. 47–57, Oct. 2019.

[S295] G. Yoo, S. Seo, S. Hong, and H. Kim, "Emotion extraction based on multi bio-signal using back-propagation neural network," *Multimed. Tools Appl.*, vol. 77, no. 4, pp. 4925–4937, Feb. 2018.

[S296] X. Xu, Y. Zhang, M. Tang, H. Gu, S. Yan, and J. Yang, "Emotion Recognition Based on Double Tree Complex Wavelet Transform and Machine Learning in Internet of Things," *IEEE Access*, vol. 7, pp. 154114–154120, 2019.

[S297] T. Chen, H. Yin, X. Yuan, Y. Gu, F. Ren, and X. Sun, "Emotion recognition based on fusion of long short-term memory networks and SVMs," *Digit. Signal Process.*, vol. 117, p. 103153, Oct. 2021.

[S298] E. Cerezo, I. Hupont, S. Baldassarri, and S. Ballano, "Emotional facial sensing and multimodal fusion in a continuous 2D affective space," *J. Ambient Intell. Humaniz. Comput.*, vol. 3, no. 1, pp. 31–46, Mar. 2012.

[S299] W.-L. Zheng, W. Liu, Y. Lu, B.-L. Lu, and A. Cichocki, "EmotionMeter: A Multimodal Framework for Recognizing Human Emotions," *IEEE Trans. Cybern.*, vol. 49, no. 3, pp. 1110–1122, Mar. 2019.

[S300] C. F. Liew and T. Yairi, "Facial Expression Recognition and Analysis: A Comparison Study of Feature Descriptors," *IPSJ Trans. Comput. Vis. Appl.*, vol. 7, pp. 104–120, 2015.

[S301] M. Yu, H. Zheng, Z. Peng, J. Dong, and H. Du, "Facial expression recognition based on a multi-task global-local network," *Pattern Recognit. Lett.*, vol. 131, pp. 166–171, Mar. 2020.

[S302] K. Mohan, A. Seal, O. Krejcar, and A. Yazidi, "FER-net: facial expression recognition using deep neural net," *Neural Comput. Appl.*, vol. 33, no. 15, pp. 9125–9136, Aug. 2021.

[S303] S. Lee, D. K. Han, and H. Ko, "Fusion-ConvBERT: Parallel Convolution and BERT Fusion for Speech Emotion

Recognition," *Sensors*, vol. 20, no. 22, p. 6688, Nov. 2020.
[S304] Y.-L. Wu, C.-T. Yeh, W.-C. Hung, and C.-Y. Tang, "Gaze direction estimation using support vector machine with active appearance model," *Multimed. Tools Appl.*, vol. 70, no. 3, pp. 2037–2062, Jun. 2014.
[S305] A. Wosiak and A. Dura, "Hybrid Method of Automated EEG Signals' Selection Using Reversed Correlation Algorithm for Improved Classification of Emotions," *Sensors*, vol. 20, no. 24, p. 7083, Dec. 2020.
[S306] T. Wang, "Intelligent employment rate prediction model based on a neural computing framework and human–computer interaction platform," *Neural Comput. Appl.*, vol. 32, no. 21, pp. 16413–16426, Nov. 2020.
[S307] D. Griol, J. M. Molina, and Z. Callejas, "Modeling the user state for context-aware spoken interaction in ambient assisted living," *Appl. Intell.*, vol. 40, no. 4, pp. 749–771, Jun. 2014.
[S308] N.-H. Ho, H.-J. Yang, S.-H. Kim, and G. Lee, "Multimodal Approach of Speech Emotion Recognition Using Multi-Level Multi-Head Fusion Attention-Based Recurrent Neural Network," *IEEE Access*, vol. 8, pp. 61672–61686, 2020.
[S309] H. Lai, K. Wu, and L. Li, "Multimodal emotion recognition with hierarchical memory networks," *Intell. Data Anal.*, vol. 25, no. 4, pp. 1031–1045, Jul. 2021.
[S310] X. Chang and W. Skarbek, "Multi-Modal Residual Perceptron Network for Audio–Video Emotion Recognition," *Sensors*, vol. 21, no. 16, p. 5452, Aug. 2021.
[S311] H. Bo, L. Ma, Q. Liu, R. Xu, and H. Li, "Music-evoked emotion recognition based on cognitive principles inspired EEG temporal and spectral features," *Int. J. Mach. Learn. Cybern.*, vol. 10, no. 9, pp. 2439–2448, Sep. 2019.
[S312] P. Chhikara, P. Singh, R. Tekchandani, N. Kumar, and M. Guizani, "Federated Learning Meets Human Emotions: A Decentralized Framework for Human–Computer Interaction for IoT Applications," *IEEE Internet Things J.*, vol. 8, no. 8, pp. 6949–6962, Apr. 2021.
[S313] C. Tan, M. Šarlija, and N. Kasabov, "NeuroSense: Short-term emotion recognition and understanding based on spiking neural network modelling of spatio-temporal EEG patterns," *Neurocomputing*, vol. 434, pp. 137–148, 2021.
[S314] S. M. Ghosh, S. Bandyopadhyay, and D. Mitra, "Nonlinear classification of emotion from EEG signal based on maximized mutual information," *Expert Syst. Appl.*, vol. 185, no. 5, p. 115605, Dec. 2021.
[S315] G. Zhang, T. Luo, W. Pedrycz, M. A. El-Meligy, M. A. F. Sharaf, and Z. Li, "Outlier Processing in Multimodal Emotion Recognition," *IEEE Access*, vol. 8, pp. 55688–55701, 2020.
[S316] P. M. Ferreira, F. Marques, J. S. Cardoso, and A. Rebelo, "Physiological Inspired Deep Neural Networks for Emotion Recognition," *IEEE Access*, vol. 6, pp. 53930–53943, 2018.
[S317] Y. Fu, H. V. Leong, G. Ngai, M. X. Huang, and S. C. F. Chan, "Physiological mouse: toward an emotion-aware mouse," *Univers. Access Inf. Soc.*, vol. 16, no. 2, pp. 365–379, Jun. 2017.
[S318] K. Zvarevashe and O. O. Olugbara, "Recognition of speech emotion using custom 2D-convolution neural network deep learning algorithm," *Intell. Data Anal.*, vol. 24, no. 5, pp. 1065–1086, Sep. 2020.
[S319] P. I. Rani and K. Muneeswaran, "Recognize the facial emotion in video sequences using eye and mouth temporal Gabor features," *Multimed. Tools Appl.*, vol. 76, no. 7, pp. 10017–10040, 2017.
[S320] H. Li and G. Wen, "Sample awareness-based personalized facial expression recognition," *Appl. Intell.*, vol. 49, no. 8, pp. 2956–2969, 2019.
[S321] J. Hu, C. Wang, Q. Jia, Q. Bu, R. Sutcliffe, and J. Feng, "ScalingNet: Extracting features from raw EEG data for emotion recognition," *Neurocomputing*, vol. 463, pp. 177–184, Nov. 2021.
[S322] Q. Chen, I. Chaturvedi, S. Ji, and E. Cambria, "Sequential fusion of facial appearance and dynamics for depression recognition," *Pattern Recognit. Lett.*, vol. 150, pp. 115–121, Oct. 2021.
[S323] V. M. Praseetha and P. P. Joby, "Speech emotion recognition using data augmentation," *Int. J. Speech Technol.*, no. January, Aug. 2021.
[S324] D. Li, J. Liu, Z. Yang, L. Sun, and Z. Wang, "Speech emotion recognition using recurrent neural networks with directional self-attention," *Expert Syst. Appl.*, vol. 173, no. February, p. 114683, Jul. 2021.
[S325] O. Starostenko, X. Cortés, J. A. Sánchez, and V. Alarcon-Aquino, "Unobtrusive emotion sensing and interpretation in smart environment," *J. Ambient Intell. Smart Environ.*, vol. 7, no. 1, pp. 59–83, 2015.
[S326] S. N. Fatima and E. Erzin, "Use of affect context in dyadic interactions for continuous emotion recognition," *Speech Commun.*, vol. 132, no. December 2019, pp. 70–82, Sep. 2021.
[S327] M. R. Dias da Silva and M. Postma, "Wandering minds, wandering mice: Computer mouse tracking as a method to detect mind wandering," *Comput. Human Behav.*, vol. 112, p. 106453, Nov. 2020.
[S328] A. Dingli and A. Giordimaina, "Webcam-based detection of emotional states," *Vis. Comput.*, vol. 33, no. 4, pp. 459–469, Apr. 2017.
[S329] F. Gullà, S. Ceccacci, R. Menghi, and M. Germani, "An adaptive smart system to foster disabled and elderly people in kitchen-related task," in *Proceedings of the 9th ACM International Conference on PErvasive Technologies Related to Assistive Environments*, 2016, vol. 29-June-20, no. June 2016, pp. 1–4.
[S330] F. M. Cau, M. S. Mancosu, F. Mulas, P. Pilloni, and L. D. Spano, "An intelligent interface for supporting coaches in providing running feedback," in *Proceedings of the 13th Biannual Conference of the Italian SIGCHI Chapter on Designing the next interaction - CHItaly '19*, 2019, no. May 2020, pp. 1–5.
[S331] S. Luo, J. Zhou, H. B.-L. Duh, and F. Chen, "BVP Feature Signal Analysis for Intelligent User Interface," in *Proceedings of the 2017 CHI Conference Extended Abstracts on Human Factors in Computing Systems*, 2017, vol. Part F1276, no. May, pp. 1861–1868.
[S332] R. Alnanih, O. Ormandjieva, and T. Radhakrishnan, "Context-based and Rule-based Adaptation of Mobile User Interfaces in mHealth," *Procedia Comput. Sci.*, vol. 21, no. 0, pp. 390–397, 2013.
[S333] K. Kurane, M. Takimoto, and Y. Kambayashi, "Design of an Intelligent Interface for the Software Mobile Agents using Augmented Reality," in *Proceedings of the 5th International Conference on Agents and Artificial Intelligence*, 2013, vol. 1, pp. 439–442.

[S334] V. Gandhi, G. Prasad, D. Coyle, L. Behera, and T. M. McGinnity, "EEG-Based Mobile Robot Control Through an Adaptive Brain–Robot Interface," *IEEE Trans. Syst. Man, Cybern. Syst.*, vol. 44, no. 9, pp. 1278–1285, Sep. 2014.

[S335] I. R. Keck and R. J. Ross, "Exploring customer specific KPI selection strategies for an adaptive time critical user interface," in *Proceedings of the 19th international conference on Intelligent User Interfaces*, 2014, no. February 2015, pp. 341–346.

[S336] S. Cheng and Y. Liu, "Eye-tracking based adaptive user interface: implicit human-computer interaction for preference indication," *J. Multimodal User Interfaces*, vol. 5, no. 1–2, pp. 77–84, Mar. 2012.

[S337] R. Z. Abidin, H. Arshad, S. A. isya. A. Shukri, and M. F. Ling, "Leveraging multimodal interaction and adaptive interfaces for location-based augmented reality Islamic tourism application," *Int. J. Adv. Sci. Eng. Inf. Technol.*, vol. 8, no. 4–2, pp. 1784–1791, 2018.

[S338] A. Holzinger, M. Geier, and P. Germanakos, "On the Development of Smart Adaptive User Interfaces for Mobile e-Business Applications - Towards Enhancing User Experience – Some Lessons Learned," in *Proceedings of the International Conference on Data Communication Networking, e-Business and Optical Communication Systems*, 2012, pp. 205–214.

[S339] E. Chae, J. Lee, J. Hwang, and H. Pak, "THE INTELLIGENT USER INTERFACE SYSTEM BASED ON 3D DIGITAL ACTOR AND UTILIZATION," *J. Theor. Appl. Inf. Technol.*, vol. 97, no. 18, pp. 4825–4836, 2019.

[S340] H. Koesling, A. Kenny, A. Finke, H. Ritter, S. McLoone, and T. Ward, "Towards intelligent user interfaces: Anticipating actions in computer games," *ACM Int. Conf. Proceeding Ser.*, no. January, 2011.

[S341] M. Mahmoud, T. Baltrušaitis, P. Robinson, and L. D. Riek, "3D Corpus of Spontaneous Complex Mental States," in *Lecture Notes in Computer Science (including subseries Lecture Notes in Artificial Intelligence and Lecture Notes in Bioinformatics)*, vol. 6974 LNCS, no. PART 1, 2011, pp. 205–214.

[S342] C.-M. Chang, B.-H. Su, S.-C. Lin, J.-L. Li, and C.-C. Lee, "A bootstrapped multi-view weighted Kernel fusion framework for cross-corpus integration of multimodal emotion recognition," in *2017 Seventh International Conference on Affective Computing and Intelligent Interaction (ACII)*, 2017, vol. 2018-Janua, pp. 377–382.

[S343] A. Sepas-Moghaddam, A. Etemad, P. L. Correia, and F. Pereira, "A Deep Framework for Facial Emotion Recognition using Light Field Images," in *2019 8th International Conference on Affective Computing and Intelligent Interaction (ACII)*, 2019, pp. 1–7.

[S344] S. Bhattacharya, "A linear regression model to detect user emotion for touch input interactive systems," in *2015 International Conference on Affective Computing and Intelligent Interaction, ACII 2015*, 2015, pp. 970–975.

[S345] H. Lee, S. Lee, Y. S. Choi, Youngwan Seo, and Eunsoo Shim, "A new posture monitoring system for preventing physical illness of smartphone users," in *2013 IEEE 10th Consumer Communications and Networking Conference (CCNC)*, 2013, pp. 713–716.

[S346] Y. Lin, T. Dai, T. Wang, and H. Wu, "A Robust Real-Time System for Multi-Intensity AU Detection in Facial Expression Recognition," in *2018 1st Asian Conference on Affective Computing and Intelligent Interaction, ACII Asia 2018*, 2018, pp. 6–11.

[S347] A. Dhall and R. Goecke, "A temporally piece-wise fisher vector approach for depression analysis," in *2015 International Conference on Affective Computing and Intelligent Interaction, ACII 2015*, 2015, pp. 255–259.

[S348] R. Henriques, A. Paiva, and C. Antunes, "Accessing Emotion Patterns from Affective Interactions Using Electrodermal Activity," in *2013 Humaine Association Conference on Affective Computing and Intelligent Interaction*, 2013, pp. 43–48.

[S349] M. Shah, D. G. Cooper, H. Cao, R. C. Gur, A. Nenkova, and R. Verma, "Action Unit Models of Facial Expression of Emotion in the Presence of Speech," in *2013 Humaine Association Conference on Affective Computing and Intelligent Interaction*, 2013, pp. 49–54.

[S350] J. Xu and Q. Zhao, "Adaptive calibration method based on state space model for eye gaze HCI system," in *2017 4th International Conference on Systems and Informatics (ICSAI)*, 2017, vol. 2018-Janua, no. Icsai, pp. 127–131.

[S351] I. Lefter and C. M. Jonker, "Aggression recognition using overlapping speech," in *2017 7th International Conference on Affective Computing and Intelligent Interaction, ACII 2017*, vol. 2018-Janua, 2017, pp. 299–304.

[S352] J. Joshi, "An Automated Framework for Depression Analysis," in *2013 Humaine Association Conference on Affective Computing and Intelligent Interaction*, 2013, pp. 630–635.

[S353] G. Wen, H. Li, and D. Li, "An ensemble convolutional echo state networks for facial expression recognition," in *2015 International Conference on Affective Computing and Intelligent Interaction, ACII 2015*, 2015, pp. 873–878.

[S354] F. Tao, G. Liu, and Q. Zhao, "An Ensemble Framework of Voice-Based Emotion Recognition System for Films and TV Programs," in *Icassp 2018*, 2018, pp. 6209–6213.

[S355] W. Q. Zheng, J. S. Yu, and Y. X. Zou, "An experimental study of speech emotion recognition based on deep convolutional neural networks," in *2015 International Conference on Affective Computing and Intelligent Interaction, ACII 2015*, 2015, pp. 827–831.

[S356] M. Soleymani, S. Asghari-Esfeden, Y. Fu, and M. Pantic, "Analysis of EEG Signals and Facial Expressions for Continuous Emotion Detection," *IEEE Trans. Affect. Comput.*, vol. 7, no. 1, pp. 17–28, Jan. 2016.

[S357] S. Carrino, E. Mugellini, O. Abou Khaled, and R. Ingold, "ARAMIS: Toward a Hybrid Approach for Human-Environment Interaction," in *Lecture Notes in Computer Science (including subseries Lecture Notes in Artificial Intelligence and Lecture Notes in Bioinformatics)*, vol. 6763 LNCS, no. PART 3, 2011, pp. 165–174.

[S358] J. Wang, M. X. Huang, G. Ngai, and H. V. Leong, "Are you stressed? Your eyes and the mouse can tell," in *2017 7th International Conference on Affective Computing and Intelligent Interaction, ACII 2017*, 2018, vol. 2018-Janua, pp. 222–228.

[S359] T. Yamauchi, A. Leontyev, and M. Razavi, "Assessing Emotion by Mouse-cursor Tracking: Theoretical and Empirical Rationales," in *2019 8th International Conference on Affective Computing and Intelligent Interaction, ACII 2019*, 2019.

[S360] H. Wang and S. Marsella, "Assessing personality through objective behavioral sensing," in *2017 7th International*

Conference on Affective Computing and Intelligent Interaction, ACII 2017, 2018, vol. 2018-Janua, pp. 131–137.
[S361] J.-L. Li and C.-C. Lee, "Attention Learning with Retrievable Acoustic Embedding of Personality for Emotion Recognition," in *2019 8th International Conference on Affective Computing and Intelligent Interaction (ACII)*, 2019, pp. 171–177.
[S362] S. Petridis, M. Leveque, and M. Pantic, "Audiovisual Detection of Laughter in Human-Machine Interaction," in *2013 Humaine Association Conference on Affective Computing and Intelligent Interaction*, 2013, pp. 129–134.
[S363] X. Ouyang *et al.*, "Audio-Visual Emotion Recognition with Capsule-like Feature Representation and Model-Based Reinforcement Learning," *2018 1st Asian Conf. Affect. Comput. Intell. Interact. ACII Asia 2018*, no. Mec 2017, 2018.
[S364] E. Velloso, A. Bulling, and H. Gellersen, "AutoBAP: Automatic Coding of Body Action and Posture Units from Wearable Sensors," in *2013 Humaine Association Conference on Affective Computing and Intelligent Interaction*, 2013, pp. 135–140.
[S365] S. Dhamija and T. E. Boult, "Automated mood-aware engagement prediction," in *2017 7th International Conference on Affective Computing and Intelligent Interaction, ACII 2017*, 2018, vol. 2018-Janua, pp. 1–8.
[S366] A. Adams and P. Robinson, "Automated recognition of complex categorical emotions from facial expressions and head motions," in *2015 International Conference on Affective Computing and Intelligent Interaction (ACII)*, 2015, pp. 355–361.
[S367] J. F. Grafsgaard, J. B. Wiggins, K. E. Boyer, E. N. Wiebe, and J. C. Lester, "Automatically Recognizing Facial Indicators of Frustration: A Learning-centric Analysis," in *2013 Humaine Association Conference on Affective Computing and Intelligent Interaction*, 2013, pp. 159–165.
[S368] S. Gievska, K. Koroveshovski, and N. Tagasovska, "Bimodal feature-based fusion for real-time emotion recognition in a mobile context," in *2015 International Conference on Affective Computing and Intelligent Interaction, ACII 2015*, 2015, pp. 401–407.
[S369] H. Yang and L. Yin, "CNN based 3D facial expression recognition using masking and landmark features," in *2017 7th International Conference on Affective Computing and Intelligent Interaction, ACII 2017*, 2018, vol. 2018-Janua, pp. 556–560.
[S370] A. Parnandi and R. Gutierrez-Osuna, "Contactless Measurement of Heart Rate Variability from Pupillary Fluctuations," in *2013 Humaine Association Conference on Affective Computing and Intelligent Interaction*, 2013, pp. 191–196.
[S371] M. C. Sun, S. H. Hsu, M. C. Yang, and J. H. Chien, "Context-aware Cascade Attention-based RNN for Video Emotion Recognition," *2018 1st Asian Conf. Affect. Comput. Intell. Interact. ACII Asia 2018*, 2018.
[S372] E. Pei, D. Jiang, M. Alioscha-Perez, and H. Sahli, "Continuous affect recognition with weakly supervised learning," *Multimed. Tools Appl.*, vol. 78, no. 14, pp. 19387–19412, 2019.
[S373] N. Banda, A. Engelbrecht, and P. Robinson, "Continuous emotion recognition using a particle swarm optimized NARX neural network," in *2015 International Conference on Affective Computing and Intelligent Interaction, ACII 2015*, 2015, pp. 380–386.
[S374] M. Dahmane and J. Meunier, "Continuous Emotion Recognition Using Gabor Energy Filters," in *Affective Computing and Intelligent Interaction, Lecture Notes in Computer Science,* 2011, pp. 351–358.
[S375] D. Heger, R. Mutter, C. Herff, F. Putze, and T. Schultz, "Continuous Recognition of Affective States by Functional Near Infrared Spectroscopy Signals," in *2013 Humaine Association Conference on Affective Computing and Intelligent Interaction*, 2013, pp. 832–837.
[S376] J. Deng, F. Eyben, B. Schuller, and F. Burkhardt, "Deep neural networks for anger detection from real life speech data," in *2017 Seventh International Conference on Affective Computing and Intelligent Interaction Workshops and Demos (ACIIW)*, 2017, vol. 2018-Janua, pp. 1–6.
[S377] Y. Cho, N. Bianchi-Berthouze, and S. J. Julier, "DeepBreath: Deep learning of breathing patterns for automatic stress recognition using low-cost thermal imaging in unconstrained settings," *2017 7th Int. Conf. Affect. Comput. Intell. Interact. ACII 2017*, vol. 2018-Janua, pp. 456–463, 2017.
[S378] J. Shen *et al.*, "Depression Detection from Electroencephalogram Signals Induced by Affective Auditory Stimuli," in *2019 8th International Conference on Affective Computing and Intelligent Interaction (ACII)*, 2019, pp. 76–82.
[S379] F. B. Pokorny, F. Graf, F. Pernkopf, and B. W. Schuller, "Detection of negative emotions in speech signals using bags-of-audio-words," *2015 Int. Conf. Affect. Comput. Intell. Interact. ACII 2015*, pp. 879–884, 2015.
[S380] N. Tateyama, K. Ueda, and M. Nakao, "Development of an active sensing system for distress detection using skin conductance response," in *2019 8th International Conference on Affective Computing and Intelligent Interaction (ACII)*, 2019, pp. 1–6.
[S381] W. Zhao, Z. Zhao, and C. Li, "Discriminative-CCA Promoted by EEG signals for Physiological-based Emotion Recognition," *2018 1st Asian Conf. Affect. Comput. Intell. Interact. ACII Asia 2018*, 2018.
[S382] E. Kroupi, A. Yazdani, and T. Ebrahimi, "EEG correlates of different emotional states elicited during watching music videos," in *Lecture Notes in Computer Science (including subseries Lecture Notes in Artificial Intelligence and Lecture Notes in Bioinformatics)*, 2011, vol. 6975 LNCS, no. PART 2, pp. 457–466.
[S383] Y. Liu, O. Sourina, and M. R. Hafiyyandi, "EEG-Based Emotion-Adaptive Advertising," in *2013 Humaine Association Conference on Affective Computing and Intelligent Interaction*, 2013, pp. 843–848.
[S384] W. Liu, X. Yu, B. Raj, L. Yi, X. Zou, and M. Li, "Efficient autism spectrum disorder prediction with eye movement: A machine learning framework," in *2015 International Conference on Affective Computing and Intelligent Interaction (ACII)*, 2015, pp. 649–655.
[S385] J. Huang, J. Tao, B. Liu, Z. Lian, and M. Niu, "Efficient Modeling of Long Temporal Contexts for Continuous Emotion Recognition," in *2019 8th International Conference on Affective Computing and Intelligent Interaction (ACII)*, 2019, vol. 8, pp. 185–191.

[S386] J.-H. Choi and J.-S. Lee, "EmbraceNet for Activity: A Deep Multimodal Fusion Architecture for Activity Recognition," in *Adjunct Proceedings of the 2019 ACM International Joint Conference on Pervasive and Ubiquitous Computing and Proceedings of the 2019 ACM International Symposium on Wearable Computers*, 2019, vol. 7, pp. 693–698.

[S387] S. Jerritta, M. Murugappan, K. Wan, and S. Yaacob, "Emotion detection from QRS complex of ECG signals using hurst exponent for different age groups," in *Proceedings - 2013 Humaine Association Conference on Affective Computing and Intelligent Interaction, ACII 2013*, 2013, pp. 849–854.

[S388] D. Girardi, F. Lanubile, and N. Novielli, "Emotion detection using noninvasive low cost sensors," in *2017 7th International Conference on Affective Computing and Intelligent Interaction, ACII 2017*, 2018, vol. 2018-Janua, pp. 125–130.

[S389] L. Tian, J. D. Moore, and C. Lai, "Emotion recognition in spontaneous and acted dialogues," in *2015 International Conference on Affective Computing and Intelligent Interaction (ACII)*, 2015, pp. 698–704.

[S390] M. Sharma and R. Mathew, "Emotion Recognition Using Physiological Signals," in *Lecture Notes on Data Engineering and Communications Technologies*, 2020, vol. 49, pp. 389–396.

[S391] Y. Zhu, X. Tian, G. Wu, G. Gasso, S. Wang, and S. Canu, "Emotional influence on SSVEP based BCI," in *Proceedings - 2013 Humaine Association Conference on Affective Computing and Intelligent Interaction, ACII 2013*, 2013, pp. 859–864.

[S392] F. Calefato, F. Lanubile, and N. Novielli, "EmoTxt: A toolkit for emotion recognition from text," in *2017 7th International Conference on Affective Computing and Intelligent Interaction Workshops and Demos, ACIIW 2017*, 2018, vol. 2018-Janua, no. July, pp. 79–80.

[S393] R. Harper and J. Southern, "End-To-End Prediction of Emotion from Heartbeat Data Collected by a Consumer Fitness Tracker," in *2019 8th International Conference on Affective Computing and Intelligent Interaction (ACII)*, 2019, pp. 1–7.

[S394] J. C. Lin, C. H. Wu, and W. L. Wei, "Error weighted semi-coupled hidden markov model for audio-visual emotion recognition," *IEEE Trans. Multimed.*, vol. 14, no. 1, pp. 142–156, 2012.

[S395] S. Ghosh, N. Ganguly, B. Mitra, and P. De, "Evaluating effectiveness of smartphone typing as an indicator of user emotion," in *2017 7th International Conference on Affective Computing and Intelligent Interaction, ACII 2017*, 2018, vol. 2018-Janua, pp. 146–151.

[S396] L. Chittaro and R. Sioni, "Exploring Eye-Blink Startle Response as a Physiological Measure for Affective Computing," in *2013 Humaine Association Conference on Affective Computing and Intelligent Interaction*, 2013, pp. 227–232.

[S397] J. O'Dwyer, N. Murray, and R. Flynn, "Eye-based Continuous Affect Prediction," in *2019 8th International Conference on Affective Computing and Intelligent Interaction (ACII)*, 2019, pp. 137–143.

[S398] A. M. Ali, I. Alkabbany, A. Farag, I. Bennett, and A. Farag, "Facial action units detection under pose variations using deep regions learning," in *2017 7th International Conference on Affective Computing and Intelligent Interaction, ACII 2017*, 2017, vol. 2018-Janua, pp. 395–400.

[S399] J. Chen, T. Takiguchi, and Y. Ariki, "Facial expression recognition with multithreaded cascade of rotation-invariant HOG," *2015 Int. Conf. Affect. Comput. Intell. Interact. ACII 2015*, pp. 636–642, 2015.

[S400] S. Makeig, G. Leslie, T. Mullen, D. Sarma, N. Bigdely-Shamlo, and C. Kothe, "First Demonstration of a Musical Emotion BCI," in *Lecture Notes in Computer Science*, vol. 3671, 2011, pp. 487–496.

[S401] S. Alghowinem, "From Joyous to Clinically Depressed: Mood Detection Using Multimodal Analysis of a Person's Appearance and Speech," in *2013 Humaine Association Conference on Affective Computing and Intelligent Interaction*, 2013, pp. 648–654.

[S402] P. Mishra and R. Sharma, "Gender Differentiated Convolutional Neural Networks for Speech Emotion Recognition," in *2020 12th International Congress on Ultra Modern Telecommunications and Control Systems and Workshops (ICUMT)*, 2020, vol. 2020-Octob, pp. 142–148.

[S403] B. Nojavanasghari, C. E. Hughes, T. Baltrusaitis, and L.-P. Morency, "Hand2Face: Automatic synthesis and recognition of hand over face occlusions," in *2017 Seventh International Conference on Affective Computing and Intelligent Interaction (ACII)*, 2017, vol. 2018-Janua, pp. 209–215.

[S404] S. Alghowinem, R. Goecke, M. Wagner, G. Parkerx, and M. Breakspear, "Head Pose and Movement Analysis as an Indicator of Depression," in *2013 Humaine Association Conference on Affective Computing and Intelligent Interaction*, 2013, pp. 283–288.

[S405] H. Lopez-Carral, D. Santos-Pata, R. Zucca, and P. F. M. J. Verschure, "How you type is what you type: Keystroke dynamics correlate with affective content," in *2019 8th International Conference on Affective Computing and Intelligent Interaction (ACII)*, 2019, pp. 1–5.

[S406] L. Li *et al.*, "Hybrid Deep Neural Network--Hidden Markov Model (DNN-HMM) Based Speech Emotion Recognition," in *2013 Humaine Association Conference on Affective Computing and Intelligent Interaction*, 2013, pp. 312–317.

[S407] I. Daly *et al.*, "Identifying music-induced emotions from EEG for use in brain-computer music interfacing," in *2015 International Conference on Affective Computing and Intelligent Interaction, ACII 2015*, 2015, vol. 22, pp. 923–929.

[S408] X. Sun, M. Lv, C. Quan, and F. Ren, "Improved facial expression recognition method based on ROI deep convolutional neutral network," in *2017 7th International Conference on Affective Computing and Intelligent Interaction, ACII 2017*, 2018, vol. 2018-Janua, pp. 256–261.

[S409] M. Hashemian, R. Prada, P. A. Santos, J. Dias, and S. Mascarenhas, "Inferring Emotions from Touching Patterns," in *2019 8th International Conference on Affective Computing and Intelligent Interaction (ACII)*, 2019, pp. 1–7.

[S410] K. Nazemi, C. Stab, and D. W. Fellner, "Interaction Analysis for Adaptive User Interfaces," in *Lecture Notes in Computer Science*, 2010, vol. 3671, pp. 362–371.

[S411] P. Fan, I. Gonzalez, V. Enescu, H. Sahli, and D. Jiang, "Kalman filter-based facial emotional expression recognition," in *Lecture Notes in Computer Science (including subseries Lecture Notes in Artificial Intelligence and Lecture Notes in Bioinformatics)*, 2011, vol. 6974 LNCS, no. PART 1, pp. 497–506.

[S412] J. Liu, W. Han, H. Ruan, X. Chen, D. Jiang, and H. Li, "Learning Salient Features for Speech Emotion Recognition Using CNN," in *2018 1st Asian Conference on Affective Computing and Intelligent Interaction, ACII Asia 2018*, 2018, pp. 0–

4.
[S413] Y. Kim and E. M. Provost, "Leveraging inter-rater agreement for audio-visual emotion recognition," in *2015 International Conference on Affective Computing and Intelligent Interaction, ACII 2015*, 2015, pp. 553–559.
[S414] M. C. Oveneke, I. Gonzalez, W. Wang, D. Jiang, and H. Sahli, "Monocular 3D facial information retrieval for automated facial expression analysis," in *2015 International Conference on Affective Computing and Intelligent Interaction, ACII 2015*, 2015, pp. 623–629.
[S415] P. Soleimaninejadian, M. Zhang, Y. Liu, and S. Ma, "Mood Detection and Prediction Based on User Daily Activities," in *2018 First Asian Conference on Affective Computing and Intelligent Interaction (ACII Asia)*, 2018, no. 61672311, pp. 1–6.
[S416] T. Yamauchi, "Mouse Trajectories and State Anxiety: Feature Selection with Random Forest," in *2013 Humaine Association Conference on Affective Computing and Intelligent Interaction*, 2013, pp. 399–404.
[S417] L. He, D. Jiang, and H. Sahli, "Multimodal depression recognition with dynamic visual and audio cues," in *2015 International Conference on Affective Computing and Intelligent Interaction (ACII)*, 2015, no. Au 14, pp. 260–266.
[S418] M. Soleymani, M. Pantic, and T. Pun, "Multimodal emotion recognition in response to videos (Extended abstract)," in *2015 International Conference on Affective Computing and Intelligent Interaction (ACII)*, 2015, pp. 491–497.
[S419] Q. Xu, B. Sun, J. He, B. Rong, L. Yu, and P. Rao, "Multimodal Facial Expression Recognition Based on Dempster-Shafer Theory Fusion Strategy," in *2018 1st Asian Conference on Affective Computing and Intelligent Interaction, ACII Asia 2018*, 2018, no. 61501035.
[S420] N. Thammasan, J. L. Hagad, K. I. Fukui, and M. Numao, "Multimodal stability-sensitive emotion recognition based on brainwave and physiological signals," *2017 7th Int. Conf. Affect. Comput. Intell. Interact. Work. Demos, ACIIW 2017*, vol. 2018-Janua, pp. 44–49, 2018.
[S421] G. Chanel, S. Avry, G. Molinari, M. Betrancourt, and T. Pun, "Multiple users' emotion recognition: Improving performance by joint modeling of affective reactions," in *2017 7th International Conference on Affective Computing and Intelligent Interaction, ACII 2017*, 2018, vol. 2018-Janua, pp. 92–97.
[S422] Q. Rao, X. Qu, Q. Mao, and Y. Zhan, "Multi-pose facial expression recognition based on SURF boosting," in *2015 International Conference on Affective Computing and Intelligent Interaction (ACII)*, 2015, pp. 630–635.
[S423] J. Seo and H. Park, "Object Recognition in Very Low Resolution Images Using Deep Collaborative Learning," *IEEE Access*, vol. 7, pp. 134071–134082, 2019.
[S424] X. Zhang, Z. Zhang, L. Yin, D. Hipp, and P. Gerhardstein, "Perception driven 3D facial expression analysis based on reverse correlation and normal component," in *2015 International Conference on Affective Computing and Intelligent Interaction, ACII 2015*, 2015, pp. 616–622.
[S425] Y. Ding, L. Shi, and Z. Deng, "Perceptual enhancement of emotional mocap head motion: An experimental study," in *2017 Seventh International Conference on Affective Computing and Intelligent Interaction (ACII)*, 2017, vol. 2018-Janua, pp. 242–247.
[S426] E. Kroupi, J.-M. Vesin, and T. Ebrahimi, "Phase-Amplitude Coupling between EEG and EDA While Experiencing Multimedia Content," in *2013 Humaine Association Conference on Affective Computing and Intelligent Interaction*, 2013, pp. 865–870.
[S427] Q. Gan, C. Wu, S. Wang, and Q. Ji, "Posed and spontaneous facial expression differentiation using deep Boltzmann machines," in *2015 International Conference on Affective Computing and Intelligent Interaction, ACII 2015*, 2015, pp. 643–648.
[S428] B. Ma, T. Greer, M. Sachs, A. Habibi, J. Kaplan, and S. Narayanan, "Predicting Human-Reported Enjoyment Responses in Happy and Sad Music," in *2019 8th International Conference on Affective Computing and Intelligent Interaction, ACII 2019*, 2019, pp. 607–613.
[S429] F. Eyben, B. Huber, E. Marchi, D. Schuller, and B. Schuller, "Real-time robust recognition of speakers' emotions and characteristics on mobile platforms," in *2015 International Conference on Affective Computing and Intelligent Interaction (ACII)*, 2015, pp. 778–780.
[S430] J. Joshi, A. Dhall, R. Goecke, and J. F. Cohn, "Relative Body Parts Movement for Automatic Depression Analysis," in *2013 Humaine Association Conference on Affective Computing and Intelligent Interaction*, 2013, pp. 492–497.
[S431] Y. Shan, T. Chen, L. Yao, Z. Wu, W. Wen, and G. Liu, "Remote Detection and Classification of Human Stress Using a Depth Sensing Technique," in *2018 First Asian Conference on Affective Computing and Intelligent Interaction (ACII Asia)*, 2018, no. 61301297, pp. 1–6.
[S432] B. Cheng *et al.*, "Robust emotion recognition from low quality and low bit rate video: A deep learning approach," in *2017 7th International Conference on Affective Computing and Intelligent Interaction, ACII 2017*, 2018, vol. 2018-Janua, pp. 65–70.
[S433] E. Tzinis and A. Potamianos, "Segment-based speech emotion recognition using recurrent neural networks," in *2017 Seventh International Conference on Affective Computing and Intelligent Interaction (ACII)*, 2017, vol. 2018-Janua, pp. 190–195.
[S434] A. Jalali, R. Mallipeddi, and M. Lee, "Sensitive deep convolutional neural network for face recognition at large standoffs with small dataset," *Expert Syst. Appl.*, vol. 87, pp. 304–315, Nov. 2017.
[S435] X. Chen, W. Han, H. Ruan, J. Liu, H. Li, and D. Jiang, "Sequence-to-sequence Modelling for Categorical Speech Emotion Recognition Using Recurrent Neural Network," in *2018 1st Asian Conference on Affective Computing and Intelligent Interaction, ACII Asia 2018*, 2018.
[S436] J. Deng, Z. Zhang, E. Marchi, and B. Schuller, "Sparse Autoencoder-Based Feature Transfer Learning for Speech Emotion Recognition," in *2013 Humaine Association Conference on Affective Computing and Intelligent Interaction*, 2013, pp. 511–516.
[S437] M. Perusquia-Hernandez, M. Hirokawa, and K. Suzuki, "Spontaneous and posed smile recognition based on spatial and temporal patterns of facial EMG," in *2017 Seventh International Conference on Affective Computing and Intelligent*

Interaction (ACII), 2017, vol. 2018-Janua, pp. 537–541.

[S438] M. Soury and L. Devillers, "Stress Detection from Audio on Multiple Window Analysis Size in a Public Speaking Task," in *2013 Humaine Association Conference on Affective Computing and Intelligent Interaction*, 2013, pp. 529–533.

[S439] H.-J. Lee and D. Lee, "Study of Process-Focused Assessment Using an Algorithm for Facial Expression Recognition Based on a Deep Neural Network Model," *Electronics*, vol. 10, no. 1, p. 54, Dec. 2020.

[S440] I. Hupont, S. Baldassarri, E. Cerezo, and R. Del-Hoyo, "The Emotracker: Visualizing Contents, Gaze and Emotions at a Glance," in *2013 Humaine Association Conference on Affective Computing and Intelligent Interaction*, 2013, pp. 751–756.

[S441] J. Orlosky, T. Toyama, D. Sonntag, and K. Kiyokawa, "The Role of Focus in Advanced Visual Interfaces," *KI - Künstliche Intelligenz*, vol. 30, no. 3–4, pp. 301–310, Oct. 2016.

[S442] S. Latif, J. Qadir, and M. Bilal, "Unsupervised Adversarial Domain Adaptation for Cross-Lingual Speech Emotion Recognition," in *2019 8th International Conference on Affective Computing and Intelligent Interaction (ACII)*, 2019, pp. 732–737.

[S443] M. Atcheson, V. Sethu, and J. Epps, "Using Gaussian Processes with LSTM Neural Networks to Predict Continuous-Time, Dimensional Emotion in Ambiguous Speech," in *2019 8th International Conference on Affective Computing and Intelligent Interaction, ACII 2019*, 2019.

[S444] X. Xia, J. Liu, T. Yang, D. Jiang, W. Han, and H. Sahli, "Video Emotion Recognition using Hand-Crafted and Deep Learning Features," in *2018 1st Asian Conference on Affective Computing and Intelligent Interaction, ACII Asia 2018*, 2018.

[S445] T. Ren, H. Ruan, W. Han, T. Yang, and D. Jiang, "Video-based Emotion Recognition Using Multi-dichotomy RNN-DNN," in *2018 First Asian Conference on Affective Computing and Intelligent Interaction (ACII Asia)*, 2018, pp. 1–6.

[S446] J. Wijsman, B. Grundlehner, H. Liu, J. Penders, and H. Hermens, "Wearable Physiological Sensors Reflect Mental Stress State in Office-Like Situations," in *2013 Humaine Association Conference on Affective Computing and Intelligent Interaction*, 2013, pp. 600–605.

[S447] J. Xie, X. Xu, and L. Shu, "WT Feature Based Emotion Recognition from Multi-channel Physiological Signals with Decision Fusion," *2018 1st Asian Conf. Affect. Comput. Intell. Interact. ACII Asia 2018*, 2018.

[S448] S. Medjden, N. Ahmed, and M. Lataifeh, "Adaptive user interface design and analysis using emotion recognition through facial expressions and body posture from an RGB-D sensor," *PLoS One*, vol. 15, no. 7, p. e0235908, Jul. 2020.

[S449] M. H. Miraz, M. Ali, P. S. Excell, and S. Khan, "AI-Based Culture Independent Pervasive M-Learning Prototype Using UI Plasticity Design," *Comput. Mater. Contin.*, vol. 68, no. 1, pp. 1021–1039, 2021.

[S450] A. Ghazarian and S. M. Noorhosseini, "Automatic detection of users' skill levels using high-frequency user interface events," *User Model. User-adapt. Interact.*, vol. 20, no. 2, pp. 109–146, Jun. 2010.

[S451] S. W. Kortschot and G. A. Jamieson, "Classification of Attentional Tunneling Through Behavioral Indices," *Hum. Factors J. Hum. Factors Ergon. Soc.*, vol. 62, no. 6, pp. 973–986, Sep. 2020.

[S452] S. W. Kortschot, G. A. Jamieson, and A. Prasad, "Detecting and Responding to Information Overload With an Adaptive User Interface," *Hum. Factors J. Hum. Factors Ergon. Soc.*, p. 001872082096434, Oct. 2020.

[S453] X. Lou, X. A. Li, P. Hansen, and P. Du, "Hand-adaptive user interface: improved gestural interaction in virtual reality," *Virtual Real.*, vol. 25, no. 2, pp. 367–382, Jun. 2021.

[S454] K. Todi, J. Jokinen, K. Luyten, and A. Oulasvirta, "Individualising Graphical Layouts with Predictive Visual Search Models," *ACM Trans. Interact. Intell. Syst.*, vol. 10, no. 1, pp. 1–24, Jan. 2020.
